# METEOR: a data-adaptive Mendelian randomization method for powerful detection of shared and specific exposures underlying multiple outcomes

**DOI:** 10.1093/bib/bbag364

**Published:** 2026-07-06

**Authors:** Liye Zhang, Ran Yan, Weiming Gong, Xiang Zhou, Lu Liu, Zhongshang Yuan

**Affiliations:** Department of Biostatistics, School of Public Health, Cheeloo College of Medicine, Shandong University, Jinan, Shandong 250012, China; Institute for Medical Dataology, Cheeloo College of Medicine, Shandong University, Jinan, Shandong 250012, China; Department of Biostatistics, School of Public Health, Cheeloo College of Medicine, Shandong University, Jinan, Shandong 250012, China; Institute for Medical Dataology, Cheeloo College of Medicine, Shandong University, Jinan, Shandong 250012, China; Department of Biostatistics, School of Public Health, Cheeloo College of Medicine, Shandong University, Jinan, Shandong 250012, China; Institute for Medical Dataology, Cheeloo College of Medicine, Shandong University, Jinan, Shandong 250012, China; Department of Biostatistics, University of Michigan, Ann Arbor, MI 48109, United States; Center for Statistical Genetics, University of Michigan, Ann Arbor, MI 48109, United States; Department of Biostatistics, School of Public Health, Cheeloo College of Medicine, Shandong University, Jinan, Shandong 250012, China; Institute for Medical Dataology, Cheeloo College of Medicine, Shandong University, Jinan, Shandong 250012, China; Department of Biostatistics, School of Public Health, Cheeloo College of Medicine, Shandong University, Jinan, Shandong 250012, China; Institute for Medical Dataology, Cheeloo College of Medicine, Shandong University, Jinan, Shandong 250012, China

**Keywords:** multimorbidity, multiple-outcomes Mendelian randomization, self-adaptive determination of instrumental variable, likelihood

## Abstract

Accurate identification of causal exposures for multimorbidity can benefit the co-prevention and co-management of multiple-related outcomes. This goal can be conceptually addressed within a multi-outcome Mendelian randomization (MR) framework. However, existing multi-outcome MR methods suffer from restrictions on format and availability of data inputs, fail to account for the potential sample overlap, rely on pre-selected independent instrumental variables (IVs), and are unable to account for horizontal pleiotropy. Here, we propose METEOR, a novel MR method that jointly models one exposure and multiple outcomes to identify both shared and outcome-specific causal exposures. METEOR accounts for sample overlap between exposure and outcomes, allows outcomes from different genome-wide association studies (GWAS) datasets, self-adaptively determines IVs from correlated single-nucleotide polymorphisms, and explicitly models horizontal pleiotropy. Using summary statistics, METEOR infers causal effects under a joint-likelihood framework with a scalable, sampling-based algorithm. Simulations show that METEOR presents well-calibrated $P$-values for both global and single-outcome tests, and achieves average power improvements of 55.33% and 56.50% over five existing MR methods in the global and single tests, respectively. In real data applications, METEOR produces the most accurate causal effect estimates in positive control analyses, reduces false positives by 18.75% in negative control analyses, and highlights that controlling BMI could benefit the co-management of multiple cardiovascular diseases (CVDs) and multiple gastrointestinal (GI) diseases, while controlling blood pressure could benefit the co-management of multimorbidity across CVDs and mental disorders (MDs), as well as across GI diseases and MDs.

## Introduction

Multimorbidity refers to the simultaneous presence of two or more chronic conditions in an individual [1]. For example, it is commonly observed within cardiovascular diseases (CVDs), such as the co-occurrence of atrial fibrillation (AF) and coronary heart disease (CHD) [2, 3] or of heart failure (HF) and myocardial infarction (MI) [4, 5]. Similarly, patterns exist within mental disorders (MDs), such as the co-occurrence of depression (DP) and anxiety disorders (AN) [6–9]. Moreover, multimorbidity often spans across CVDs and MDs, such as the co-occurrence of DP with CHD, HF, and MI [10–12], where the prevalence of DP is 15%-30% in patients with CHD [13]. Patients with multimorbidity often experience worse treatment outcomes compared to those with a single disease. For example, DP could reduce the chances of successful modifications of cardiac risk factors [14] and participation in cardiac rehabilitation [15, 16]. Accurate identification of causal exposures contributing to multimorbidity could benefit co-prevention and co-management for multiple related outcomes. This can be conceptually achieved by using a multi-outcome Mendelian randomization (MR) framework.

MR uses only summary statistics from genome-wide association studies (GWASs) and serves as an efficiently statistical tool to investigate the causal associations between exposures and outcomes in observational studies, leveraging genetic variants, typically single-nucleotide polymorphisms (SNPs), as instrumental variables (IVs) [17, 18]. For an IV to be valid, it must be associated with the exposure, independent of confounders of the exposure-outcome pair, and affect the outcome only through the exposure. Many MR methods have been recently developed [19–33]. However, all these methods are designed for single-outcome analysis and cannot be readily extended to handle multiple related outcomes. Analysing one outcome at a time inevitably ignores correlations among them, potentially leading to a loss of power in multi-outcome MR settings. To the best of our knowledge, only five multi-outcome MR methods, including MRMO [34], BMRMO [35], MR-AHC [36], MR^2^ [37], and MrDAG [38], have been proposed to model multiple outcomes together (Supplementary Table S1). However, all of these methods face three important modeling challenges that have so far limited their effectiveness in identifying both shared and outcome-specific causal exposures.

First, a fundamental challenge in MR analyses is the potential bias induced by sample overlap between exposure GWAS and outcome GWAS. While some single-outcome MR methods have been developed to address this issue (e.g. MR-APSS [30]), most existing multi-outcome MR methods fail to simultaneously account for the potential sample overlap between the exposure GWAS and any of the outcome GWASs, and some even require that all outcomes come from the same GWAS dataset. For example, MR^2^ employs a sparse Bayesian Gaussian copula regression framework and is unable to account for sample overlap between the exposure and outcome GWASs, which, as we will show here, can lead to false causal discoveries. Similarly, MR-AHC, MRMO, and BMRMO also assume no sample overlap between exposure and outcome samples. These limitations in data input requirements hinder the generalization and broader application of these methods. Indeed, GWASs for both exposure and multiple outcomes are often obtained from large-scale meta-analysis, which typically combine the associations from multiple studies, consequently increasing the likelihood of sample overlap between exposure and outcome datasets. In addition, the multiple outcomes of interest may be from different GWASs rather than from the same GWAS dataset, making overlap induced bias more pronounced in the multi-outcome setting.

Second, all five existing multi-outcome MR methods, including MR^2^, MrDAG, MRMO, BMRMO, MR-AHC, rely on a pre-selected set of independent or weakly correlated SNPs as IVs, typically obtained through linkage disequilibrium (LD) clumping of genome-wide significant SNPs in the exposure GWAS. However, using only a small number of independent SNPs may capture a limited proportion of the exposure variance, thereby reducing statistical power [27, 31, 39], especially given that complex traits are often influenced by many SNPs in LD. Furthermore, this pre-selection approach of IVs fails to account for the uncertainty that SNPs may influence the outcomes through either the exposure or exposure-independent paths. Previous studies have demonstrated that incorporating IV selection process within univariate MR analysis can improve the accuracy of causal effect estimates [27, 31]. However, selecting the IVs from a set of correlated SNPs becomes more challenging in a multi-outcome MR setting, where a SNP may serve as a valid IV in inferring the exposure effect on one outcome while simultaneously exhibiting horizontal pleiotropic effects when inferring the exposure effect on other outcomes. Therefore, it is desirable to develop multi-outcome MR methods that can account for the uncertainty of different roles of SNPs in investigating the causal association between an exposure and multiple related outcomes.

Third, most existing multi-outcome MR methods fail to account for horizontal pleiotropic effects, which occurs when SNPs influence the outcomes through pathways independent of the exposure. The presence of horizontal pleiotropy has been well documented to bias the causal effect estimates and increase false discoveries in MR analysis. While several single-outcome MR methods [20, 26–31] have explicitly account for pleiotropic effects, this remains largely unaddressed in current multi-outcome MR methods. Among existing methods, MR^2^ computes the non-zero residual correlation between summary-level outcomes using all SNPs, assumes the unmeasured pleiotropic pathway is shared across at least two outcomes and reflects global unmeasured shared pleiotropy. Although MR^2^ is also able to detect potential outlier SNPs with local pleiotropic effects, it fails to explicitly model the outcome-specific pleiotropy. MrDAG is a Bayesian graphical modeling method aiming to infer dependency structures among exposures and outcomes, and accounts for the unmeasured shared pleiotropy similar to MR^2^. MR-AHC focuses on heterogeneity across SNPs and acknowledges that one source of heterogeneity in the variant-specific estimates is horizontal pleiotropy, which is also not directly adjusted in MR-AHC. In addition, both MRMO and BMRMO do not account for pleiotropic effects.

In this study, we develop METEOR, a novel Multi-outcomE MR method for the idenTification of sharEd and Outcome-specific exposuRes. METEOR jointly models one exposure and multiple outcomes, accounts for sample overlap between the exposure and outcomes as well as among outcomes, allows the outcomes to be from different GWAS datasets, self-adaptively selects IVs from a set of correlated SNPs, and efficiently models the horizontal pleiotropy. METEOR estimates the correlation matrix among the exposure and multiple outcomes using genome-wide summary statistics, conducts causal inference within a joint likelihood framework using a scalable sampling-based algorithm to obtain well-calibrated $P$-values. We assess the advantages of METEOR through extensive simulations. In the real data analysis, we conduct a positive control analysis to examine the causal effect of each lipid trait on itself, a negative control analysis to investigate the causal effects of each lipid trait on hair color (HC) and skin color (SC), and two shared exposure detection analyses to identify metabolic risk factors for the multimorbidity across CVDs and MDs underlying the brain–heart axis, as well as across gastrointestinal (GI) diseases and MDs underlying the brain–gut axis.

## Materials and methods

### METEOR for individual-level data

Our goal is to simultaneously estimate and test the causal effects of an exposure on multiple outcomes, while self-adaptively accounting for horizontal pleiotropy and the correlations between the exposure and each outcome, as well as among outcomes, due to potential sample overlap. We consider a general model framework in which multiple outcomes may not necessarily come from the same dataset, and sample overlap may exist among these multiple outcomes as well as between the exposure and each outcome. We denote $\boldsymbol{x}$ as an ${n}_1$-vector of the exposure for ${n}_1$ individuals in the exposure GWAS and ${\boldsymbol{y}}_k$ as an ${n}_{2k}$-vector of the $k$-th outcome for ${n}_{2k}$ individuals in the $k$-th outcome GWAS, where $k=1,\cdots, K$. We initially select $p$ SNPs associated with the exposure with marginal $P$-values below $5\times{10}^{-8}$. These SNPs are likely in LD with each other and are utilized as correlated candidate IVs. We denote ${\boldsymbol{G}}_x$ as an ${n}_1\times p$ genotype matrix for these SNPs in the exposure dataset, and ${\boldsymbol{G}}_{y_k}$ as an ${n}_{2k}\times p$ genotype matrix for the same SNPs in the $k$-th outcome dataset. We scale $\boldsymbol{x}$,${\boldsymbol{y}}_k$ and each column of the genotype matrices (${\boldsymbol{G}}_x$ and ${\boldsymbol{G}}_{y_k}$). We develop METEOR as follows:


(1) \begin{equation*} \boldsymbol{x}={\boldsymbol{G}}_x\boldsymbol{\beta} +{\boldsymbol{\varepsilon}}_x, \end{equation*}

(2) \begin{equation*} {\boldsymbol{y}}_k={\alpha}_k{\boldsymbol{G}}_{y_k}\boldsymbol{\beta} +{\boldsymbol{G}}_{y_k}{\boldsymbol{\eta}}_k+{\boldsymbol{\varepsilon}}_{y_k},k=1,\cdots, K. \end{equation*}

where $\boldsymbol{\beta}$ is a $p$-vector of correlated SNP effect sizes on the exposure; ${\alpha}_k$ is a scalar that represents the causal effect of the exposure on the $k$-th outcome; ${\boldsymbol{\eta}}_k$ is a $p$-vector of horizontal pleiotropic effects on the $k$-th outcome; ${\boldsymbol{\varepsilon}}_x$ and ${\boldsymbol{\varepsilon}}_{y_k}$ are ${n}_1$-vector and ${n}_{2k}$-vector of residual errors, respectively. For any two outcomes, e.g. the ${k}_1$-th and ${k}_2$-th outcomes for ${k}_1,{k}_2\in \left[1,\cdots, K\right]$, each element of residual errors for those shared individuals follows $MVN\left(\left(\begin{array}{@{}c@{}}0\\{}0\end{array}\right),\left(\begin{array}{@{}cc@{}}{\sigma}_{y_{k_1}}^2& {\rho}_{y_{k_1},{y}_{k_2}}{\sigma}_{y_{k_1}}{\sigma}_{y_{k_2}}\\{}{\rho}_{y_{k_1},{y}_{k_2}}{\sigma}_{y_{k_1}}{\sigma}_{y_{k_2}}& {\sigma}_{y_{k_2}}^2\end{array}\right)\right)$, where ${\rho}_{y_{k_1},{y}_{k_2}}$ is a correlation coefficient, while the each element of residual errors for those non-overlapped individuals independently follows $N\left(0,{\sigma}_{y_{k_1}}^2\right)$ or $N\left(0,{\sigma}_{y_{k_2}}^2\right)$, respectively. The non-zero correlation between any two outcomes for the shared individuals can be leveraged to enhance the power of detecting causal associations. Similarly, we assume each element of residual errors for those individuals shared in exposure and the $k$-th outcome follows: $MVN\left(\left(\begin{array}{@{}c@{}}0\\{}0\end{array}\right),\left(\begin{array}{@{}cc@{}}{\sigma}_x^2& {\rho}_{x,{y}_k}{\sigma}_x{\sigma}_{y_k}\\{}{\rho}_{x,{y}_k}{\sigma}_x{\sigma}_{y_k}& {\sigma}_{y_k}^2\end{array}\right)\right)$, where ${\rho}_{x,{y}_k}$ is a correlation coefficient. Each element of the residual errors for those non-overlapped individuals from exposure or the $k$-th outcome independently follows $N\left(0,{\sigma}_x^2\right)$ or $N\left(0,{\sigma}_{y_k}^2\right)$, respectively. We derive the causal interpretation and identification of the causal effects under the decision-theoretic framework of causal inference [40–43] (details in Supplementary Notes 1–2).

### METEOR for summary-level data

Due to the privacy concerns, individual-level data from GWASs are often unavailable. Instead, most GWASs provide summary statistics in terms of marginal z-scores. Thus, we extend the above individual-level model to the version with GWAS summary statistics. Specifically, we denote the LD structure of the candidate instrumental SNPs as ${\boldsymbol{\Sigma}}_x$ in the exposure GWAS dataset, and ${\boldsymbol{\Sigma}}_{y_k}$ in the $k$-th outcome GWAS dataset. The proposed METEOR model for summary statistics can be constructed as follows:


(3) \begin{equation*} {\boldsymbol{z}}_x=\sqrt{n_1-1}{\boldsymbol{\Sigma}}_x\boldsymbol{\beta} +{\boldsymbol{\epsilon}}_x, \end{equation*}

(4) \begin{equation*} {\boldsymbol{z}}_{y_k}=\sqrt{n_{2k}-1}{\boldsymbol{\Sigma}}_{y_k}\boldsymbol{\beta} {\alpha}_k+\sqrt{n_{2k}-1}{\boldsymbol{\Sigma}}_{y_k}{\boldsymbol{\eta}}_k+{\boldsymbol{\epsilon}}_{y_k},k=1,\cdots, K. \end{equation*}

where ${\boldsymbol{z}}_x$ is a $p$-vector of marginal z-scores with ${n}_1$ individuals in the exposure GWAS; ${{\boldsymbol{z}}_y}_k$ is a $p$-vector of marginal z-scores with ${n}_{2k}$ individuals in the $k$-th outcome GWAS; both ${\boldsymbol{\epsilon}}_x$ and ${\boldsymbol{\epsilon}}_{y_k}$ are $p$-vector of residual errors for exposure and the $k$-th outcome, respectively. Often, ${\boldsymbol{\Sigma}}_x$ and ${\boldsymbol{\Sigma}}_{y_k}$ are from the same LD reference panel (e.g. 1000 Genomes project [44]), i.e. ${\boldsymbol{\Sigma}}_x={\boldsymbol{\Sigma}}_{y_1}=\cdots{\boldsymbol{\Sigma}}_{y_K}=\boldsymbol{\Sigma}$. In addition, we denote $\mathbf{E}=\left({\boldsymbol{\epsilon}}_x,{\boldsymbol{\epsilon}}_{y_1},\cdots, {\boldsymbol{\epsilon}}_{y_K}\right)$, which follows a matrix normal distribution $M{N}_{p,K+1}\left(\mathbf{0},\boldsymbol{\Sigma}, \boldsymbol{\Omega} \right)$. Equations (3) and (4) can be further combined as follows:


(5) \begin{equation*} \left({\boldsymbol{z}}_x,{\boldsymbol{z}}_{y_1},\cdots, {\boldsymbol{z}}_{y_K}\right)\sim MN\left(\left(E\left[{\boldsymbol{z}}_x\right],E\left[{\boldsymbol{z}}_{y_1}\right],\cdots, E\left[{\boldsymbol{z}}_{y_K}\right]\right),\boldsymbol{\Sigma}, \boldsymbol{\Omega} \right), \end{equation*}

(6) \begin{equation*} E\left[{\boldsymbol{z}}_x\right]=\sqrt{n_1-1}\boldsymbol{\Sigma} \boldsymbol{\beta}, \end{equation*}

(7) \begin{equation*} E\left[{\boldsymbol{z}}_{y_k}\right]=\sqrt{n_{2k}-1}\boldsymbol{\Sigma} \left(\boldsymbol{\beta} {\alpha}_k+{\boldsymbol{\eta}}_k\right),k=1,\cdots, K, \end{equation*}

(8) \begin{equation*} \boldsymbol{\Omega} =\left(\begin{array}{cccc}{\sigma}_x^2& {\rho}_{x,{y}_1}{\sigma}_x{\sigma}_{y_1}& \cdots & {\rho}_{x,{y}_K}{\sigma}_x{\sigma}_{y_K}\\{}{\rho}_{x,{y}_1}{\sigma}_{y_1}{\sigma}_x& {\sigma}_{y_1}^2& \cdots & {\rho}_{y_1,{y}_K}{\sigma}_{y_1}{\sigma}_{y_K}\\{}\cdots & \cdots & \cdots & \cdots \\{}{\rho}_{x,{y}_K}{\sigma}_{y_K}{\sigma}_x& {\rho}_{y_1,{y}_K}{\sigma}_{y_K}{\sigma}_{y_1}& \cdots & {\sigma}_{y_K}^2\end{array}\right). \end{equation*}

In formula (5), $MN$ is used to denote the matrix normal distribution. The $p\times p$ matrix $\boldsymbol{\Sigma}$ characterizes the covariance across the candidate instrumental SNPs due to LD. The $\left(K+1\right)\times \left(K+1\right)$ matrix $\boldsymbol{\Omega}$ characterizes the covariance across the exposure and $K$ outcomes, where the diagonal elements are used to quantify the inflation of test statistics due to confounding biases for each trait, and the off-diagonal elements are used to characterize the correlation of estimation errors induced by sample overlap between traits.

We denote $\boldsymbol{z}={\left({\boldsymbol{z}}_x^T,{\boldsymbol{z}}_{y_1}^T,\cdots, {\boldsymbol{z}}_{y_K}^T\right)}^T$, $\boldsymbol{\epsilon} ={\left({\boldsymbol{\epsilon}}_x^T,{\boldsymbol{\epsilon}}_{y_1}^T,\cdots, {\boldsymbol{\epsilon}}_{y_K}^T\right)}^T$,$\boldsymbol{\xi} ={\left(\sqrt{n_1-1},\sqrt{n_{21}-1},\cdots, \sqrt{n_{2K}-1}\right)}^T$, $\boldsymbol{\alpha} ={\left(1,{\alpha}_1,\cdots, {\alpha}_K\right)}^T$, $\boldsymbol{\eta} ={\left({\boldsymbol{\eta}}_0^T,{\boldsymbol{\eta}}_1^T,\cdots, {\boldsymbol{\eta}}_K^T\right)}^T$, where ${\boldsymbol{\eta}}_0$ is a $p$-vector with all elements being zero. Then,


(9) \begin{equation*} \boldsymbol{z}={\mathbf{I}}_{K+1}\otimes \left(\boldsymbol{\Sigma} \boldsymbol{\beta} \right)\cdotp \left(\boldsymbol{\xi} \circ \boldsymbol{\alpha} \right)+{\mathbf{I}}_{K+1}\otimes \boldsymbol{\Sigma} \cdotp \left(\left(\boldsymbol{\xi} \otimes{\mathbf{1}}_p\right)\circ \boldsymbol{\eta} \right)+\boldsymbol{\epsilon}, \end{equation*}

(10) \begin{equation*} \boldsymbol{z}\sim MVN\left(\boldsymbol{E}\left[\boldsymbol{z}\right],\boldsymbol{\Omega} \otimes \boldsymbol{\Sigma} \right)\kern-2pt, \end{equation*}

(11) \begin{equation*} \boldsymbol{E}\left[\boldsymbol{z}\right]={\left(E{\left[{\boldsymbol{z}}_x\right]}^T,E{\left[{\boldsymbol{z}}_{y_1}\right]}^T,\cdots, E{\left[{\boldsymbol{z}}_{y_K}\right]}^T\right)}^T. \end{equation*}

where ${\mathbf{I}}_{K+1}$ is a $K+1$ by $K+1$ identity matrix, ${\mathbf{1}}_p$ is a $p$-vector with all elements equal to one, $\otimes$ denotes Kronecker product, and the term $\left(\boldsymbol{\xi} \circ \boldsymbol{\alpha} \right)$ represents Hadamard product, also known as element-wise product, of the two vectors $\boldsymbol{\xi}$ and $\boldsymbol{\alpha}$.

### Model assumptions and inference

Some of the $P$ exposure-associated correlated candidate SNPs may be in LD with the true causal SNPs, or may be the false signals in the exposure GWAS. We make a sparse assumption that ${\beta}_j\sim{\pi}_{\beta }N\left(0,{\sigma}_{\beta}^2\right)+\left(1-{\pi}_{\beta}\right){\delta}_0$ for $j=1,\cdots, p$, where ${\delta}_0$ is Dirac function that represents a point mass at zero. That is, with probability $1-{\pi}_{\beta }$, the $j$-th SNP has zero effect on the exposure, while with probability ${\pi}_{\beta }$, the $j$-th SNP has a non-zero effect on the exposure and its effect size follows $N\left(0,{\sigma}_{\beta}^2\right)$. We assume ${\sigma}_{\beta}^2$ follows an inverse gamma distribution ${\sigma}_{\beta}^2\sim InvG\left({a}_{\beta },{b}_{\beta}\right)$, and set ${a}_{\beta }=\frac{p}{10}+1,{b}_{\beta }=0.2$ to ensure a prior mean of $\frac{2}{p}$.

The selected SNPs with non-zero effects on the exposure (${\beta}_j\ne 0$) can affect the $k$-th outcome either through causal effect ${\alpha}_k$ or through horizontal pleiotropic effects ${\boldsymbol{\eta}}_k$. For the $k$-th outcome, we assume each selected SNP has a probability of ${\pi}_{1k}$ to display horizontal pleiotropy, with the effect size follows $N\left(0,{\sigma}_k^2\right)$. The unselected SNPs with zero effects on the exposure (${\beta}_j=0$) can affect the $k$-th outcome only through the horizontal pleiotropy, we assume each unselected SNP has a probability of ${\pi}_{0k}$ to display horizontal pleiotropy, with the effect size follows $N\left(0,{\sigma}_k^2\right)$. We assume ${\sigma}_k^2\sim InvG\left({a}_k,{b}_k\right)$, with ${a}_k=\frac{p}{5}+1$ and ${b}_k=0.2$ to ensure a prior mean of $\frac{1}{p}$. Here, we use the same variance parameter ${\sigma}_k^2$ to represent the effect sizes of the horizontal pleiotropy for both selected and unselected SNPs, given that we may not have enough SNPs to accurately estimate two separate parameters. Additionally, the inclusion of unselected SNPs could reduce residual variance and improve the precision of the causal effect estimates (Supplementary Notes 3–5, 7).

We first adopt the LD score regression (LDSC) method to estimate the matrix parameter $\boldsymbol{\Omega}$,


$$ \hat{\boldsymbol{\Omega}}=\left(\begin{array}{@{}cccc@{}}{\omega}_0& {\omega}_{0,1}& \cdots & {\omega}_{0,K}\\{}{\omega}_{0,1}& {\omega}_1& \cdots & {\omega}_{1,K}\\{}\cdots & \cdots & \cdots & \cdots \\{}{\omega}_{0,K}& {\omega}_{1,K}& \cdots & {\omega}_K\end{array}\right), $$

where ${\omega}_0$ and ${\omega}_k$ ($k=1,\cdots K$) are the intercepts estimated from single-trait LDSC for the exposure and the $k$-th outcome, respectively, which are used to adjust the biases in estimation errors [45]. While ${\omega}_{0,k}$ is the intercept estimated from bivariate LDSC to account for the correlation of the estimation errors between the exposure and the $k$-th outcome [46, 47], and ${\omega}_{k_1,{k}_2}$ (${k}_1,{k}_2\in \left[1,\cdots, K\right]$) is also the intercept estimated from bivariate LDSC to account for the correlation of the estimation errors among different outcomes [46, 47]. Given the estimated $\hat{\boldsymbol{\Omega}}$, we develop an algorithm for numerical integration to derive the likelihood to infer causal effects ${\boldsymbol{\alpha}}^{\ast}={\left({\alpha}_1,\cdots, {\alpha}_K\right)}^T$. Our algorithm relies on a Gibbs sampling to obtain posterior samples of ${\boldsymbol{\alpha}}^{\ast}$, and further estimate the causal effects ${\hat{\boldsymbol{\alpha}}}^{\ast }$ and their covariance matrix $Cov\left({\hat{\boldsymbol{\alpha}}}^{\ast}\right)$ (Supplementary Note 6). Next, we derive Wald statistic for global test ${\mathrm{H}}_0:{\alpha}_1=\cdots ={\alpha}_K=0$, to examine whether the exposure would affect any outcome,


$$ {T}_{Wald}={\hat{\boldsymbol{\alpha}}}^{\ast T}{\left[ Cov\left({\hat{\boldsymbol{\alpha}}}^{\ast}\right)\right]}^{-1}{\hat{\boldsymbol{\alpha}}}^{\ast}, $$

where ${T}_{Wald}\sim{\chi}_{df=K}^2$ under the null. And Wald test was used to evaluate whether the exposure affects a specific outcome $k$ (single test), ${\mathrm{H}}_{0k}:{\alpha}_k=0$,


$$ {T}_{Wal{d}_k}=\frac{{\hat{\alpha}}_k^2}{Var\left({\hat{\alpha}}_k\right)}, $$

where ${T}_{Wal{d}_k}\sim{\chi}_{df=1}^2$. Of note, the single test here is not directly derived from the traditional single-outcome MR analysis, as we have incorporated correlations between the $k$-th outcome and the other outcomes.

### Simulations

We performed extensively realistic simulations to evaluate performance of METEOR. To illustrate the benefits of METEOR, we compared it with two classes of MR methods: (i) single-outcome MR methods and (ii) multi-outcome MR methods that leverage outcome correlations to improve statistical power using GWAS summary statistics. For single-outcome MR, we included the classical IVW-R, MRAID, and MR-APSS methods. MRAID can automatically select instruments from a set of correlated SNPs and has been shown to outperform many other single-outcome MR methods [27], including RAPS, CAUSE, MRMix, Robust, Weighted median and Weighted mode. Thus, these additional methods were not included in our comparisons. In addition, MR-APSS is the recently developed single-outcome MR methods aiming to alleviate the issue of sample overlap. For multi-outcome MR, we compared METEOR with MR^2^ and MrDAG, which are currently the only two MR methods that consider the correlations of multi-outcomes to enhance power using GWAS summary statistics (details in Supplementary Note 8). We did not include MRMO, BMRMO and MR-AHC in our comparisons, as both MRMO and BMRMO require individual-level data, which is often unavailable in practice, and MR-AHC, unlike typical MR methods, does not produce a single causal effect of an exposure-outcome pair, but instead outputs multiple causal effect estimates for different clusters of genetic variants. To simulate the exposure and the outcome datasets, we randomly selected $n$ individuals from the UK Biobank and divided them into $K+1$ equal-sized sets: one for exposure with ${n}_1$ individuals and the remaining $K$ sets for the $K$ outcomes with ${n}_{21}$ to ${n}_{2K}$ individuals. Due to sample overlap, ${n}_1+{n}_{21}+\cdots +{n}_{2K}$ may not be equal to $n$. For these individuals, we obtained their genotypes for a total of 708,827 SNPs on chromosome 1.

In the exposure dataset, we randomly selected $M$ (e.g. 100) SNPs to have non-zero effects on the exposure, standardized the genotype vector of each SNP, and denoted the genotype matrix of these SNPs as ${\tilde{\boldsymbol{G}}}_1$. We simulated these SNP effect sizes on the exposure ($\boldsymbol{\beta}$) from $N\left(0,\frac{PV{E}_{{\tilde{G}}_1}}{M}\right)$, where $PV{E}_{{\tilde{G}}_1}$ represents the proportion of exposure variance explained by these genetic effects. The total genetic effects across all $M$ SNPs is ${\tilde{\boldsymbol{G}}}_1\boldsymbol{\beta}$. In the $k$-th outcome dataset, we obtained and standardized the genotype vector for the same $M$ SNPs as ${\tilde{\boldsymbol{G}}}_{2k}$, and used the same $\boldsymbol{\beta}$ to obtain ${\tilde{\boldsymbol{G}}}_{2k}\boldsymbol{\beta}$. We set the causal effect ${\alpha}_k=\sqrt{\frac{PV{E}_{\alpha k}}{PV{E}_{{\tilde{G}}_1}}}$ so that the proportion of variance in the $k$-th outcome explained by the causal effect was $PV{E}_{\alpha k}$. In addition, we randomly selected ${\pi}_{1k}M$ SNPs (rounded to an integer) from these $M$ SNPs, and $100-{\pi}_{1k}M$ (rounded to an integer) SNPs from the remaining non-causal SNPs to exhibit horizontal pleiotropy, forming a total of 100 pleiotropic SNPs denoted as ${\tilde{\boldsymbol{G}}}_{hk}$ for the $k$-th outcome. Their effect sizes ${\boldsymbol{\eta}}_k={\left({\eta}_{k1},\cdots, {\eta}_{k100}\right)}^T$ were simulated with each element obtained from $N\left(0,\frac{PV{E}_{hk}}{100}\right)$, so that the proportion of variance in the $k$-th outcome explained by horizontal pleiotropy was $PV{E}_{hk}$.

When there were correlations among the exposure and multiple outcomes due to potential sample overlap, the residual errors for each shared individual were simulated from $MVN\left(\mathbf{0},\tilde{\boldsymbol{\Omega}}\right)$ with


$$ \tilde{\boldsymbol{\Omega}}=\left(\begin{array}{@{}cccc@{}}{\tilde{\sigma}}_x^2& {\tilde{\rho}}_{x,{y}_1}{\tilde{\sigma}}_x{\tilde{\sigma}}_{y_1}& \cdots & {\tilde{\rho}}_{x,{y}_k}{\tilde{\sigma}}_x{\tilde{\sigma}}_{y_K}\\{}{\tilde{\rho}}_{x,{y}_1}{\tilde{\sigma}}_x{\tilde{\sigma}}_{y_1}& {\tilde{\sigma}}_{y_1}^2& \cdots & {\tilde{\rho}}_{y_1,{y}_k}{\tilde{\sigma}}_{y_1}{\tilde{\sigma}}_{y_K}\\{}\cdots & \cdots & \cdots & \cdots \\{}{\tilde{\rho}}_{x,{y}_K}{\tilde{\sigma}}_x{\tilde{\sigma}}_{y_K}& {\tilde{\rho}}_{y_1,{y}_K}{\tilde{\sigma}}_{y_1}{\tilde{\sigma}}_{y_K}& \cdots & {\tilde{\sigma}}_{y_K}^2\end{array}\right)\kern-2pt, $$

where ${\tilde{\sigma}}_x^2=1- PV{E}_{{\tilde{G}}_1}$, ${\tilde{\sigma}}_{y_k}^2=1- PV{E}_{\alpha k}- PV{E}_{hk}$ and ${\tilde{\rho}}_{u,v}$ was the correlation coefficient between the exposure and outcome or between different outcomes, $u,v\in \left[x,{y}_1,\cdots, {y}_K\right]$. In absence of sample overlap, the residual errors were simulated independently from $N\left(0,{\tilde{\sigma}}_x^2\right)$ and $N\left(0,{\tilde{\sigma}}_{y_k}^2\right)$, respectively. We finally summed the genetic effects and residual errors to yield the simulated exposure, and summed the causal effects, horizontal pleiotropic effects and residual errors to yield the simulated outcomes via the following equations:


$$ \boldsymbol{x}={\tilde{\boldsymbol{G}}}_1\boldsymbol{\beta} +{\boldsymbol{e}}_x, $$

$$ {\boldsymbol{y}}_k={\tilde{\boldsymbol{G}}}_{2k}\boldsymbol{\beta} {\alpha}_k+{\tilde{\boldsymbol{G}}}_{hk}{\boldsymbol{\eta}}_k+{\boldsymbol{e}}_{y_k},k=1,\cdots, K. $$

The truly exposure-associated SNPs were known in simulations, but they remained unknown for any real dataset. To mimic the real MR analysis, we treated them as unknown and conducted GWAS analysis for the exposure using PLINK to select SNPs with $P<5\times{10}^{-8}$ as the candidate correlated SNPs. For these selected SNPs, we also performed association analysis in the $k$-th outcome dataset ($k=1,\dots K$), and obtained the $z$-scores for both exposure and outcomes as the summary statistics. We denoted the genotype matrices of these selected SNPs from exposure and outcomes as ${\tilde{\boldsymbol{Z}}}_1$ and ${\tilde{\boldsymbol{Z}}}_{2k}$, respectively. We can also obtain the in-sample LD matrices as ${\boldsymbol{\Sigma}}_1=\frac{\ {{\tilde{\boldsymbol{Z}}}_1}^T\ {\tilde{\boldsymbol{Z}}}_1}{n_1-1}$ and ${\boldsymbol{\Sigma}}_{2k}=\frac{{{\tilde{\boldsymbol{Z}}}_{2k}}^T\ {\tilde{\boldsymbol{Z}}}_{2k}}{n_{2k}-1}$. Given the common LD matrix (e.g. estimated from the same LD reference panel) was often used in real MR analysis, we combined these $K$ LD matrices of outcomes and used $\boldsymbol{\Sigma} =\frac{\sum_{k=1}^K{n}_{2k}{\boldsymbol{\Sigma}}_{2k}}{\sum_{k=1}^K{n}_{2k}}$ as the input, where we assigned relatively large weight to the LD matrix obtained from the outcome GWAS with large sample size.

In the baseline simulations, we set two outcomes ($K=2$), number of SNPs with non-zero effects on exposure $M=100$, proportion of exposure variance explained by genetic effects $PV{E}_{{\tilde{G}}_1}=10\%$, proportion of variance in the $k$-th outcome explained by horizontal pleiotropy $PV{E}_{hk}=5\%$, ${\pi}_{1k}=20\%$, ${n}_1={n}_{2k}=\mathrm{50,000}\ \left(k=1,2\right)$,, and assumed there was no sample overlap between the exposure and any outcome (${\tilde{\rho}}_{x,{y}_1}={\tilde{\rho}}_{x,{y}_2}=0$), but outcomes were drawn from the same dataset with ${\tilde{\rho}}_{y_1,{y}_2}=0.5$. We examined three settings with different causal effect sizes evaluated by $\boldsymbol{PV}{\boldsymbol{E}}_{\alpha }={\left( PV{E}_{\alpha 1}, PV{E}_{\alpha 2}\right)}^T$: [1] a setting where the exposure did not affect any outcome with $\boldsymbol{PV}{\boldsymbol{E}}_{\alpha }={\left(0,0\right)}^T$, [2] a setting where the exposure affected one outcome with $\boldsymbol{PV}{\boldsymbol{E}}_{\alpha }={\left(0.075\%,0\right)}^T$, and [3] a setting where the exposure affected both outcomes with $\boldsymbol{PV}{\boldsymbol{E}}_{\alpha }={\left(0.075\%,0.075\%\right)}^T$. The parameters were set based on real data and previous studies [27, 48] to reflect realistic genetic architectures as much as possible. Based on baseline setting, we varied one parameter at a time to evaluate the impact of different parameters on the methods’ performance (Supplementary Note 9 and Table  S2). We also designed three simulations to investigate the impact of varying combinations of sample overlap proportions and correlations (Supplementary Note 10). In addition, we conducted simulations in the baseline setting to assess the robustness of METEOR under violations of normal distribution and linear genetic effects (Supplementary Note 11). Finally, we conducted simulations to assess METEOR by violating specific model assumptions, including simulations evaluating the correction of horizontal pleiotropy, the adaptively determine instruments from correlated SNPs, and the role of the correlation matrix $\boldsymbol{\Omega}$ (Supplementary Note 12).

Type I error was assessed under the null hypothesis across 500 iterations, and power was evaluated over 100 simulations (Supplementary Note 13). We used $p=5\times{10}^{-8}$ for all methods to select candidate instrumental SNPs, and performed LD clumping to select independent SNPs with ${r}^2$ being 0.01 for both IVW-R, MR-APSS, MR^2^ and MrDAG per their requirement for independent SNPs. We also evaluated the accuracy of the causal effect estimates for each method.

### Real data applications

To assess the accuracy of the causal effect estimations and the type I error control of MR methods, we applied methods to lipid-centric (total cholesterol, TC; high density cholesterol, HDL; low density cholesterol, LDL; triglycerides, TG) analyses, including a positive control analysis where each lipid trait was tested for its causal effect on itself, and a negative control analysis to examine causal effects of four lipid traits on HC and SC. Both analyses were evaluated in two-sample and one-sample MR settings. To create the potential sample overlap, we utilized individual data from UK Biobank (Supplementary Note 14).

Furthermore, we conducted shared exposure detection analyses to identify the metabolic risk factors for the multimorbidity. For brain–heart axis, we used summary data from UK Biobank for metabolic exposures (body mass index, BMI; type 2 diabetes, T2D; systolic blood pressure, SBP; diastolic blood pressure, DBP; HDL; LDL; and TG; which were also used in MR^2^) and summary data from FinnGen [49] across five CVDs (atrial fibrillation, AF; angina pectoris, AP; coronary heart disease, CHD; heart failure, HF; myocardial infarction, MI) and two MDs (DP and AN; Supplementary Note 14 and Supplementary Tables S3-S4). For brain–gut axis, we similarly investigated the metabolic risk factors for multimorbidity across four GI diseases [50, 51] (inflammatory bowel disease, irritable bowel syndrome, gastroduodenal ulcer, and gastroesophageal reflux disease; details in Supplementary Table S4) and MDs. As IVW-R, MRAID, and MR^2^ require absence of sample overlap, we ran all methods under two-sample MR setting.

## Results

### Simulations: Type I error control

**Figure 1:**
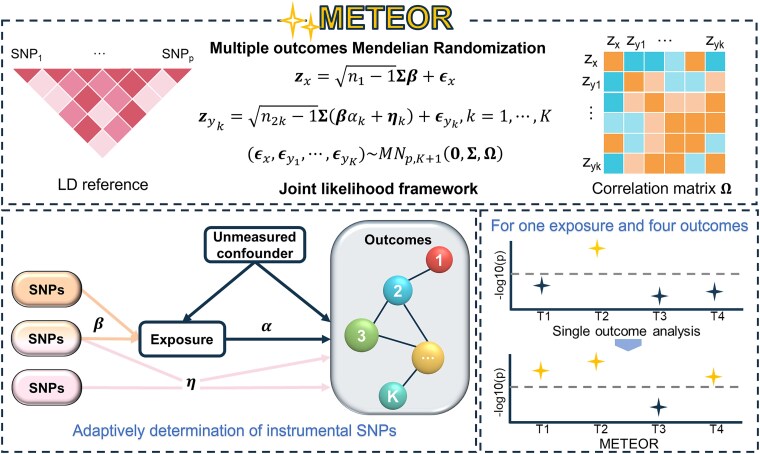
Schematic of METEOR. METEOR is a novel Mendelian randomization (MR) method for identifying the shared and specific exposures underlying multiple outcomes. METEOR utilizes an LD reference ($\boldsymbol{\Sigma}$) to account for correlated candidate SNPs significantly associated with the exposure (SNP_1_, $\dots$, SNP_p_) (top left). It uses LDSC to accurately estimate the correlation matrix ($\boldsymbol{\Omega}$) from genome-wide summary statistics (top right), then places causal inference into a joint likelihood framework (top middle, the detailed explanations of the parameters are provided in the Methods section) to ensure the statistical efficiency. METEOR leverages a set of correlated SNPs, automatically determines instrumental SNPs exhibiting vertical (orange) or horizontal (pink) pleiotropy, and self-adaptively accounts for the correlations either between the exposure and each outcome or among multiple outcomes (bottom left). In addition, METEOR tends to be more powerful than univariate outcome MR methods by utilizing correlations among multiple outcomes (T1, T2, T3 and T4) (bottom right).

METEOR was described in the Methods, with technical details provided in the Supplementary Notes and schematic shown in Fig. 1. Briefly, METEOR was a likelihood-based MR method for multiple outcomes that utilized a set of correlated SNPs, self-adaptively accounted for horizontal pleiotropy as well as the correlations among the exposure and multiple outcomes. METEOR was scalable to biobank datasets (Supplementary Table S5) and remained computationally efficient with larger number of outcomes (Supplementary Fig. S1). We compared it with IVW-R, MR-APSS, MRAID, MR^2^ and MrDAG (Supplementary Table S6) across 105 simulation settings (Supplementary Table S7).

**Figure 2:**
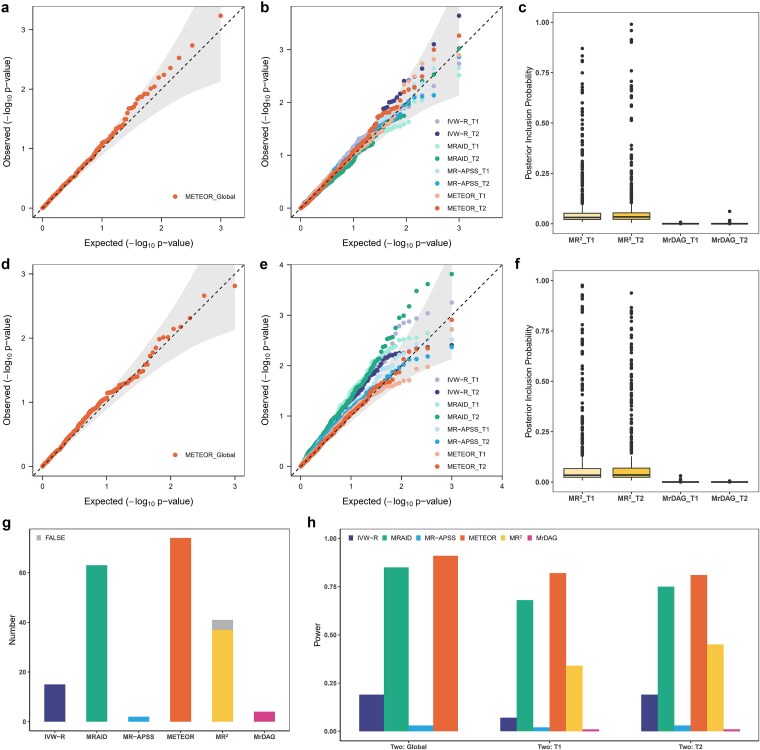
Type I error control and power from six MR methods. The type I error control is evaluated using quantile-quantile (QQ) plot of $-{\log}_{10}P$ values. (a) QQ plot from the global test of METEOR in the baseline setting. (b) QQ plots from IVW-R, MRAID, MR-APSS and METEOR in testing the causal effect of exposure on both outcomes (T1: The first outcome; T2: The second outcome) with $\boldsymbol{PV}{\boldsymbol{E}}_{\alpha }={\left(0,0\right)}^T$ in the baseline setting. (c) Posterior inclusion probabilities (PIPs) from MR^2^ and MrDAG for the two outcomes in the baseline setting. (d) QQ plot from the global test of METEOR in one-sample MR setting with sample sizes being ${n}_1={n}_{21}={n}_{22}=50000$. (e) QQ plots from IVW-R, MRAID, and METEOR in testing the causal effect of exposure on both outcomes in one-sample MR setting. (f) PIPs from MR^2^ and MrDAG for the two outcomes in one-sample MR setting. (g) Numbers of true discoveries and false discoveries (grey) for all methods in the baseline setting with $\boldsymbol{PV}{\boldsymbol{E}}_{\alpha }={\left(0.075\%,0\right)}^T$ under bonferroni adjusted $P$-value threshold of $5\times{10}^{-4}$. (h) Power performance under bonferroni adjusted $P$-value threshold of $5\times{10}^{-4}$ for global and single tests in the baseline setting. The results are plotted under one alternative setting: With $\boldsymbol{PV}{\boldsymbol{E}}_{\alpha }={\left(0.075\%,0.075\%\right)}^T$ (‘two: All’ for global test; ‘two: T1’ and ‘two: T2’ for single tests).

We initially examined type I error control under null hypothesis in baseline setting. Given that the null distributions of minimum $P$-values for IVW-R, MRAID and MR-APSS are not trivial to obtain, we only displayed the quantile–quantile (QQ) plot of METEOR in the global test, which was well-calibrated (Fig. 2a). All methods also demonstrated well-calibrated type I error control in the single tests for both outcomes (Fig. 2b). Although some PIPs exceeded 0.5, MR^2^ also demonstrated well, with the median PIPs being 0.031 and 0.034 (Fig. 2c). Similar results remained regardless of the sample sizes (Supplementary Fig. S2A-C), numbers of outcomes (Supplementary Fig. S3A-F), proportions of SNPs with horizontal pleiotropy effects (Supplementary Fig. S4), proportions of exposure variance explained by the genetic effects (Supplementary Fig. S5), proportions of phenotype variance explained by the horizontal pleiotropic effects (Supplementary Fig. S6), correlations among multiple outcomes (Supplementary Figs. S7, S8A-F,), and whether horizontal pleiotropy was considered (Supplementary Fig. S9A-C). Additionally, METEOR remained stable across various priors of ${\pi}_{\beta }$, ${\pi}_1$ and ${\pi}_0$ (Supplementary Figs. S10A-C, S11A-C, S12A-C).

We also compared methods’ performance with 0% and 100% sample overlap between the exposure and both outcomes (${n}_1={n}_{21}={n}_{22}=\mathrm{50,000}$). Both IVW-R and MRAID displayed inflated type I error control under 100% sample overlap, with the inflation factors (IFs) for the single tests of both outcomes being 1.420 and 1.425 for IVW-R, 1.363 and 1.905 for MRAID, respectively. MR^2^ also showed a slight increase in false discoveries, with the proportion of replicates with PIP$\ge$0.5 increasing from 0.036 to 0.064 for the first outcome, and from 0.040 to 0.058 for the second outcome. In contrast, METEOR, MR-APSS, and MrDAG maintained well (Fig. 2a-f). Specifically, the IF for METEOR was 1.040 for the global test, 1.156 and 1.015 for both single tests, respectively. Additionally, across all methods, the larger sample overlap corresponded to narrower confidence intervals (CIs), due to the correlation between the residuals of the exposure and outcome (Supplementary Fig. S13). In simulations without horizontal pleiotropy but with 100% sample overlap between exposure and outcomes, IVW-R and MRAID exhibited inflated type I error control, whereas METEOR, MR^2^, and MrDAG remained well-calibrated (Supplementary Fig. S14). When the sample size increased to 100,000 for both exposure and outcomes, MRAID showed improved type I error control, while IVW-R continued to exhibit substantially inflated type I error, and the false discovery rate of MR^2^ was not substantially changed and remained at ~0.060 (Supplementary Fig. S15). As expected, all methods yielded more accurate causal effects and narrow CIs in large sample size setting (Supplementary Fig. S13).

### Simulations: Power comparison

Under the Bonferroni correction ($P=5\times{10}^{-4}$), we examined power under alternative hypothesis in baseline setting. METEOR was the most powerful method in global and single tests (Fig. 2g, h), likely due to its ability in accounting for the correlations among outcomes and utilizing more correlated SNPs. When the exposure effected only one outcome, MR^2^ exhibited false discoveries for the null outcome, while METEOR yielded the highest number of true discoveries for the non-null outcome (74 versus 15, 63, 2, 37, and 4 for IVW-R, MRAID, MR-APSS, MR^2^, and MrDAG; Fig. 2g). When the exposure effected both outcomes, METEOR again demonstrated the highest power (Fig. 2h), with average improvements of 55.33% and 56.50% in the global and single tests, respectively, over other methods. Furthermore, METEOR outperformed MR^2^ and MrDAG with the corresponding areas under the ROC curve (AUC) of 0.99, 0.80, and 0.71, respectively (Supplementary Fig. S16F). Similar results were observed across a range of simulation settings (Supplementary Fig. S2D-F, S3G-H, S8G-H, S9D-F, S16-S20). These findings remained when opposite causal effects were designed (Supplementary Fig. S21). As expected, the power of all methods increased as the casual effect sizes (Supplementary Figs. S19-S20) and sample sizes increased (Supplementary Fig. S22). METEOR benefited from the increased correlations among outcomes (Supplementary Fig. S23). Importantly, METEOR was robust to various priors of ${\pi}_{\beta }$, ${\pi}_1$, and ${\pi}_0$ (Supplementary Figs. S10D, S11D, S12D). Under a series of $PV{E}_{\alpha k}$ values in the baseline setting, METEOR consistently outperformed the other methods based on the unified FDR of 0.05 (Supplementary Fig. S24).

**Figure 3:**
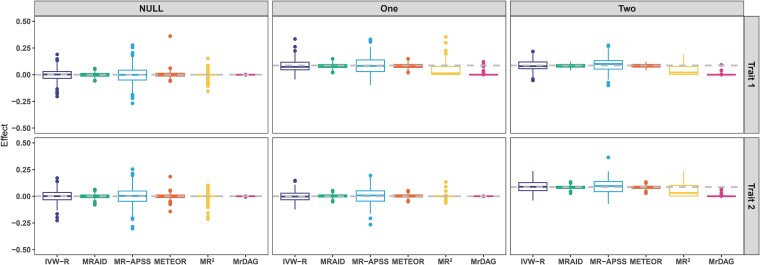
Estimates of causal effects from six MR methods in the baseline setting. The baseline setting involves one exposure and two outcomes with $PV{E}_{{\tilde{G}}_1}=10\%$, $M=100$, ${\pi}_{1k}=20\%$, $PV{E}_{hk}=5\%$, ${n}_1={n}_{2k}=\mathrm{50,000}\ \left(k=1,2\right)$, ${\tilde{\rho}}_{y_1,{y}_2}=0.5$ and ${\tilde{\rho}}_{x,{y}_1}={\tilde{\rho}}_{x,{y}_2}=0$. Three causal effect settings are displayed from left to right: (1) the null setting where the exposure has no effect on any outcome with $\boldsymbol{PV}{\boldsymbol{E}}_{\alpha }={\left(0,0\right)}^T$; (2) an alternative setting where the exposure causally affects one outcome with $\boldsymbol{PV}{\boldsymbol{E}}_{\alpha }={\left(0.075\%,0\right)}^T$ and (3) an alternative setting where the exposure causally affects both outcomes with $\boldsymbol{PV}{\boldsymbol{E}}_{\alpha }={\left(0.075\%,0.075\%\right)}^T$. The causal effect estimates for the first and second outcomes are listed from top to bottom.

We further compared the estimation accuracy of causal effects across all methods. They produced unbiased causal effect estimates in null simulations. Compared to other methods, both METEOR and MRAID showed narrower 95% CIs presumably due to the nature of their likelihood inference framework. Similar results were also observed under alternative simulations, while both MR^2^ and MrDAG underestimated the causal effects (Fig. 3). These findings retained with smaller sample sizes (${n}_1={n}_{21}={n}_{22}=\mathrm{20,000}$; Supplementary Fig. S25). Under the setting with opposite causal effects, METEOR remained accurate, whereas MR^2^ and MrDAG overestimated the negative effects, and underestimated the positive effects (Supplementary Fig. S26).

### Simulations: robustness and advantages of METEOR

We examined robustness against different proportions of sample overlap and correlations. Across all scenarios, including those with no sample overlap or correlation, with sample overlap but no correlation, and with both sample overlap and correlation, METEOR consistently showed well-calibrated type I error in both global test (Supplementary Fig. S27A, S28A, S29A, S30, Fig. 2a) and single tests (Supplementary Fig. S27B-F, S28B-C, S29B-C, S30, Fig. 2b-c). METEOR achieved the highest power than IVW-R, MRAID, and MR-APSS, particularly in the global test (Supplementary Fig. S27G, S28D-E, S29D-E), and provided better performance in detecting the true causal associations than MR^2^ and MrDAG (Supplementary Fig. S27H, S28F, S29F).

METEOR relies on normality distributional assumption and linear modeling structures. To evaluate its robustness, we conducted simulations under the baseline setting by introducing violations of these assumptions. First, to assess the robustness against the non-normality, we considered a t distribution with different degrees of freedom ($df=30$, $10$, and $5$) to represent varying levels of heavy-tailed behavior, with the small degrees of freedom representing the large deviation of normal distribution. The results showed that METEOR maintained well-controlled type I error rates across different degrees of freedom in both global and single tests (Supplementary Figs. S31-S33). The other five methods also demonstrated well-calibrated type I error control in the single tests for both outcomes. In terms of power, METEOR remained the most powerful method in global and single tests after Bonferroni correction ($P=5\times{10}^{-4}$; Supplementary Figs. S31-S33). For example, when $df=5$ and the exposure affected both outcomes, the power of global tests for IVW-R, MRAID, MR-APSS, METEOR was 0.208, 0.823, 0.052, and 0.938, respectively. For the single tests, the power of IVW-R, MRAID, MR-APSS, METEOR, MR^2^, and MrDAG was 0.167, 0.729, 0.063, 0.833, 0.406 and 0 for the first outcome, and 0.104, 0.688, 0.021, 0.833, 0.385, and 0.031 for the second outcome, respectively. METEOR again demonstrated the highest power (Supplementary Fig. S33E), with average improvements of 57.70% and 53.77% in the global and single tests, respectively, compared with other methods. Furthermore, METEOR outperformed MR^2^ and MrDAG with the corresponding AUC of 0.99, 0.81, and 0.71, respectively (Supplementary Fig. S33F). Second, to investigate the impact of nonlinear genetic effects, we introduced a quadratic term in the relationship between genetic variants and the exposure. Under this nonlinear setting, METEOR controlled type I error well in both global and single tests. However, all methods failed to detect significant signals after Bonferroni correction $\left(P=5\times{10}^{-4}\right)$, suggesting all methods, including METEOR, are vulnerable to nonlinear genetic effects (Supplementary Fig. S34).

We further assessed the methodological advantages of METEOR. In baseline setting with horizontal pleiotropy ($PV{E}_{hk}=5\%$), METEOR achieved well-calibrated type I error, while METEOR with $\boldsymbol{\eta}$ restricted to be zero produced inflated $P$-values (Supplementary Fig. S35). METEOR also gained substantial power from its self-adaptive IVs selection and its ability to model correlations among outcomes, outperforming versions using pre-specified independent instrumental SNPs (Supplementary Fig. S36), analysing outcomes separately, and restricting $\boldsymbol{\Omega}$ to be an identity matrix (Supplementary Figs. S37-S38). Additionally, METEOR with $\boldsymbol{\Omega}$ restricted to the identity matrix produced inflated type I error control (Supplementary Figs. S38-S39), due to the failure to account for sample overlap.

### Real data applications: Positive and negative control analyses

**Figure 4:**
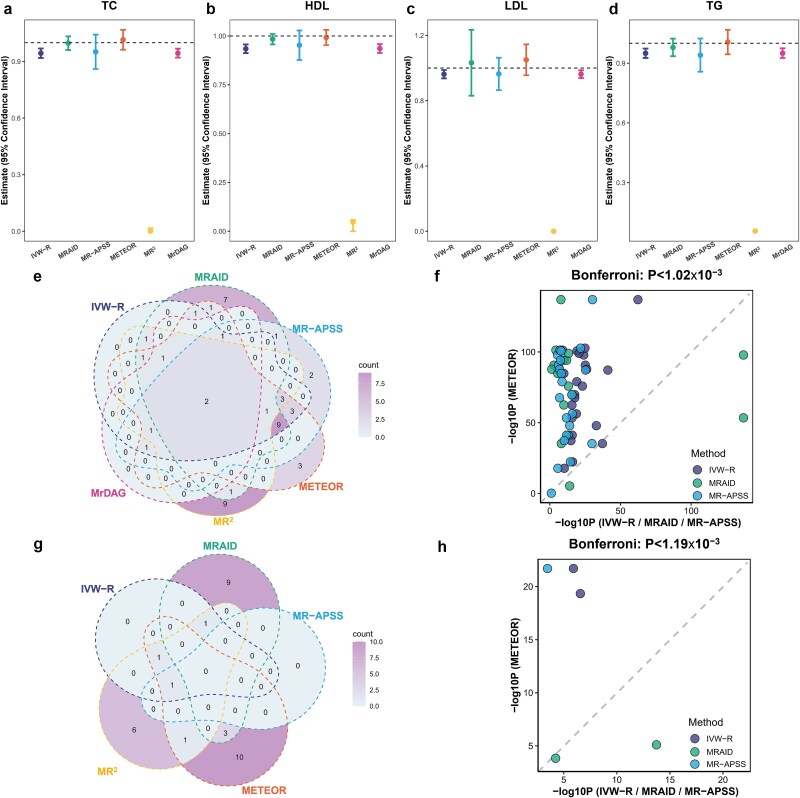
Summarized results from the positive control and shared exposure detection analyses. Point estimates and 95% confidence intervals (CIs) from IVW-R, MRAID, MR-APSS, METEOR, MR^2^, and MrDAG are shown in positive control analysis to examine the causal effect of lipid trait on itself, including (a) TC-TC, (b) HDL-HDL, (c) LDL-LDL, and (d) TG-TG. The horizontal black dashed lines in panels (a-d) represent the true causal effect size of one. The 95% confidence intervals are calculated using formula $\alpha \pm 1.96\times se$, where $\alpha$ represents the causal effect estimate and $se$ denotes its standard error. The results of the shared exposure detection analysis underlying brain-heart axis are presented in (e-f) using a Bonferroni adjusted $P$-value threshold of $1.02\times{10}^{-3}$, and underlying brain-gut axis are presented in (g-h) using a $P$-value threshold of $1.19\times{10}^{-3}$. Panels (e, g) show Venn plots for the number of causal pairs detected by MR methods, respectively. MrDAG identified no significant associations after Bonferroni correction ($P=1.19\times{10}^{-3}$); thus, only the other five methods are shown in panel G. Scatter plots of significant causal pairs with the same effect direction are shown in (f, h) for METEOR and IVW-R, for METEOR and MRAID and for METEOR and MR-APSS underlying brain-heart axis and brain-gut axis.

In positive control analysis, only METEOR, MR-APSS and MRAID were able to produce 95% CIs covering the true causal effects for all trait pairs under two-sample MR setting. In contrast, IVW-R and MrDAG presented slight underestimation, whereas MR^2^ presented substantial underestimation of the causal effects (Fig. 4a-d). More importantly, METEOR produced the relatively narrow CIs across almost all positive controls (Supplementary Table S8). For example, METEOR ($\alpha =1.051$; 95% CI: 0.956 to 1.145), MR-APSS ($\alpha =0.964$; 95% CI: 0.865 to 1.063) and MRAID ($\alpha =1.032$; 95% CI: 0.830 to 1.234) correctly estimated the causal effect of LDL on itself, with METEOR providing the best 95% CI. In contrast, IVW-R ($\alpha =0.962$; 95% CI: 0.937 to 0.988), MrDAG ($\alpha =0.962$; 95% CI: 0.939 to 0.987) and MR^2^ ($\alpha =0$; 95% CI: 0 to 0) did not cover the true causal effect. After Bonferroni correction ($p=1.25\times{10}^{-2}$; $p=3.125\times{10}^{-3}$), IVW-R, MRAID, MR-APSS and METEOR identified the positive associations for the four pairs in global and single tests (Supplementary Table S9). MrDAG also identified these associations in single tests by PIP, whereas MR^2^ missed TC-TC (PIP $=0.064$) and LDL-LDL (PIP $=0.013$). Compared with the two-sample MR setting, IVW-R and MrDAG improved in the one-sample MR setting, with 95% CIs covering the true causal effects for all pairs, but MR^2^ still failed to detect TC-TC (PIP $=0.159$) and LDL-LDL (PIP $=0.022$) (Supplementary Table S10).

In negative control analysis, IVW-R and MR^2^ falsely detected one causal pair of TG-SC ($p=1.73\times{10}^{-4}$; PIP $=0.962$) in two-sample MR setting under the Bonferroni correction ($p=6.25\times{10}^{-3}$) for the single test. In one-sample MR setting, IVW-R and MR^2^ again detected a spurious TG-SC effect ($p=5.96\times{10}^{-6}$; PIP $=0.999$), while MRAID falsely identified a significant causal effect of LDL on SC ($p=1.16\times{10}^{-5}$), suggesting that sample overlap could lead to false positives for methods that cannot account for sample overlap. In contrast, METEOR, MR-APSS and MrDAG performed well in the presence of sample overlap between exposure and outcome (Supplementary Table S11). Under a Bonferroni correction ($p=1.25\times{10}^{-2}$) for the global test, IVW-R and MRAID also identified some false signals, while both METEOR and MR-APSS did not.

### Real data applications: Shared exposure detection analyses in brain-heart and brain-gut axes

**Figure 5:**
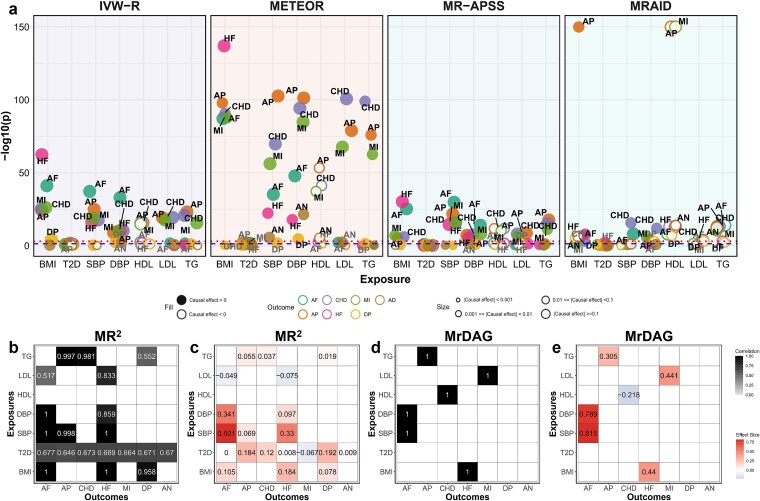
Detailed results of six MR methods in the shared exposure detection analysis for the multimorbidity of CVDs and MDs. (a) Causal effect estimates and the corresponding $P$-values from IVW-R, METEOR, MR-APSS, and MRAID. Different outcomes are represented with different colors. The direction of causal effect is indicated by solid or hollow circle, with the circle size reflecting the causal effect size. The red and blue dashed lines represent the Bonferroni adjusted $-{\mathit{\log}}_{10}p$-value threshold and $-{\mathit{\log}}_{10}$-transformed nominal significance threshold, respectively. Two-sides $p$-values are calculated for the three methods. Posterior inclusion probabilities (PIPs) of each exposure (y axis) against each outcome (x axis) from (b) MR^2^ and (d) MrDAG. Posterior mean of the causal effect of each exposure (y axis) against each outcome (x axis) from (c) MR^2^ and (e) MrDAG. For better illustration, PIPs and estimated effect sizes of non-detected exposure-outcome pairs (PIP$\le 0.5$) are not plotted.

We first conducted an analysis to identify metabolic risk factors for multimorbidity of CVDs and MDs. Under Bonferroni correction ($P=7.14\times{10}^{-3}$) for global test, METEOR identified six out of seven causal relationships (excluding T2D), with four yielding smaller $P$-values than IVW-R, MRAID and MR-APSS. All six significant global tests were consistent with the fact that there was at least one significant causal pair in the corresponding single tests, so as the remaining insignificant global test. Under Bonferroni correction ($P=1.02\times{10}^{-3}$) for single test, IVW-R, MRAID, MR-APSS and METEOR identified 25, 26, 24, and 28 pairs, respectively (Fig. 4e), while, MR^2^ and MrDAG identified 20 and 6 pairs based on PIP$\ge 0.5$. Most signals from other methods were also detected by METEOR with consistent directions and generally smaller $P$-values (Fig. 4f and Fig. 5; Supplementary Note 15). Some associations uniquely identified by other methods seemed to be counter-intuitive and inconsistent with the evidence from previous literature. For example, MRAID identified negative causal associations of BMI on DP ($\alpha =-0.035$, $P=4.60\times{10}^{-6}$) and AN ($\alpha =-0.038$, $P=6.66\times{10}^{-7}$). However, previous studies reported a substantially higher prevalence of DP and AN among people with obesity, especially with severe obesity [52–56]. Additionally, evidence from observational and MR studies supported both psychological and biological mechanisms, under the obesity-DP/AN causal relationship [52, 53, 57–61]. Similar results were obtained with a $P$-value threshold of 0.05 for the single tests (Supplementary Table S12 and Fig. 5a).

Importantly, METEOR uniquely detected several causal pairs with biologically plausible mechanisms (Fig. 4e and Supplementary Table S12). Under Bonferroni correction ($P=1.02\times{10}^{-3}$) for the single tests (Fig. 5a), METEOR identified SBP-AN ($\alpha =0.042$, $P=1.83\times{10}^{-6}$), DBP-DP ($\alpha =0.026$, $P=1.81\times{10}^{-5}$), and DBP-AN ($\alpha =0.060$, $P=3.94\times{10}^{-22}$), indicating potential positive causal effects of blood pressure on DP and AN. Often, patients with hypertensive, experience profound emotional stress, which increase the risk to develop mental health disorders, particularly DP and AN [62, 63]. It also uniquely identified four additional plausible causal pairs (Fig. 5a), such as T2D-CHD ($\alpha =0.143$, $P=0.016$) and T2D-MI ($\alpha =0.142$, $P=0.027$), which have supported by several epidemiological studies and clinical evidence [64–68].

Based on results from METEOR (Supplementary Table S12 and Note 15), BMI, LDL and TG were shared metabolic exposures for the multimorbidity within CVDs, with BMI affecting the most CVD outcomes. SBP, DBP and HDL were shared exposures for the multimorbidity across CVDs and MDs, with DBP affecting the most outcomes, followed by SBP. These findings highlight the importance of controlling BMI for co-prevention and co-management of multiple CVDs, and controlling blood pressure in co-management for multimorbidity across CVDs and MDs. Additionally, all six metabolic factors showed the causal effects on AP, CHD, and MI.

Next, we performed an analysis to identify metabolic risk factors for the multimorbidity of GI diseases and MDs. Under Bonferroni correction ($P=7.14\times{10}^{-3}$) for the global test, METEOR identified six of seven causal relationships, with single test results consistent (Supplementary Table S13 available online at https://bib.oxfordjournals.org/). Under Bonferroni correction ($P=1.19\times{10}^{-3}$) for single test, IVW-R, MRAID, MR-APSS and METEOR identified 3, 13, 1, and 16 pairs, respectively (Fig. 4g), while MR^2^ and MrDAG identified 10 and 0 pairs based on PIPs. In particular, two out of the three significant pairs identified by IVW-R and the one significant pair identified by MR-APSS were identified by METEOR with consistent effect directions and smaller $P$-values (Fig. 4h and Supplementary Table S13). However, only 2 out of the 13 significant pairs identified by MRAID and 3 out of the 10 significant pairs identified by MR^2^ were also identified by METEOR with consistent effect directions (Fig. 4h and Supplementary Table S13). Among the 11 and 7 pairs identified by MRAID and MR^2^, rather than by METEOR, some are inconsistent with previous literature. For example, MRAID also identified negative causal association of BMI on DP ($\alpha =-0.031$, $P=8.14\times{10}^{-6}$) and AN ($\alpha =-0.037$, $P=3.61\times{10}^{-7}$), consistent with the false discoveries from the above hear-brain axis analysis.

METEOR uniquely detected many biologically plausible causal pairs (Supplementary Table S13 and Note  15). For example, DBP was identified as a shared risk factor for two GI diseases and two MDs. Both BMI and LDL were shared exposures for the multimorbidity within GI diseases, with BMI affecting the most outcomes. SBP, DBP, HDL, and TG were shared exposures for the multimorbidity across GI diseases and MDs, with DBP and SBP affecting the most outcomes. These findings highlight controlling BMI could benefit co-prevention and co-management of the multiple GI diseases, and also highlight the importance of controlling blood pressure in co-management for the multimorbidity across GI diseases and MDs.

## Discussion

We have presented METEOR, an efficient MR method that leverages GWAS summary statistics to facilitate the identification of shared and outcome-specific exposures across multiple outcome traits. METEOR accounts for sample overlap and correlations between exposure and outcomes, and among multiple outcomes. It explicitly models horizontal pleiotropy, incorporates self-adaptive IV selection within a joint likelihood framework, and produces accurate causal effect estimates with well-calibrated $P$-values for both global and single-outcome tests. METEOR is applicable in a wide range of settings, regardless of the GWAS data sources for the exposure and outcomes. Through extensive simulations and applications, METEOR effectively controls type I error and achieves substantial power gains over existing univariate and multi-outcome MR methods.

By explicitly modeling the parameter $\boldsymbol{\Omega}$, METEOR is able to account for complex sample structure such as population stratification, cryptic relatedness, and sample overlap [30, 69, 70]. We primarily demonstrated the advantage of METEOR in scenarios with varying degrees of sample overlap, which can induce correlations between the exposure and outcomes as well as among multiple outcomes. Ignoring such correlation, as in IVW-R and MRAID, would lead to biased test statistics. In addition, increasing the sample size could lead to a reduction in false discoveries and narrower confidence intervals. However, it does not completely eliminate the influence introduced by sample overlap, as the correlation between the residuals of the exposure and outcome remains.

Although METEOR is formulated under the continuous outcome and linear modeling framework, linear models can be viewed as first-order Taylor approximations to logistic regression, especially in GWAS field, and are known to exhibit a certain degree of robustness to model misspecification [71]. This provides a theoretical basis for directly applying linear models to case–control outcomes. Indeed, some GWAS analyses for binary traits are often conducted using linear (mixed) models that treat case–control status as a quantitative trait, mainly due to computational efficiency and scalability. Even for those binary traits analysed using logistic regression, GWAS summary statistics are typically reported as log(OR) estimates. Under typical small effect sizes of SNPs, these estimates can be approximately interpreted as linear effects on an underlying liability scale, which makes them compatible with a linear model [72]. Such approximation has been also widely adopted in summary-level MR analyses. Taken together, METEOR can be used under a broad range of outcome types. However, the linear approximation is most accurate when genetic effect sizes are small and case–control ratio of the outcome is not extremely imbalanced [72]. This limitation is commonly shared by most summary-level MR methods, rather than specific to METEOR.

METEOR is mainly developed to infer the total causal effect of a specific exposure on each of multiple outcomes. In contrast, the MVMR approaches [73] jointly consider multiple exposures, aiming to estimates the direct causal effects of each exposure. Incorporating additional exposures allows for the modeling of indirect or mediating effects through other exposures and thus improves the inference of direct causal effects. However, extending METEOR into the multi-exposures setting is nontrivial. For example, in a setting with two exposures, METEOR would need to self-adaptively select IVs or horizontal pleiotropic SNPs across both exposures, and the role of each candidate SNP, whether it is an instrumental or pleiotropic SNP, is specific to each exposure. Furthermore, whether a SNP serves as an IV or exhibits pleiotropy when estimating the causal effect of the first exposure on certain outcomes also depends on the role of the second exposure, whether it is a confounder, mediator, or collider.

Horizontal pleiotropy occurs when the SNP instruments exhibit effects on the outcome through pathways other than the exposure. It can generally be categorized into uncorrelated pleiotropy, which arises from paths independent of the SNP effects on the exposure, and the correlated pleiotropy, which arises when SNPs are associated with unobserved exposure-outcome confounders, thereby inducing correlation between the horizontal pleiotropic effects and the SNP effects on the exposure. For instance, insulin resistance occurs when excess glucose in the blood reduces the ability of the cells to absorb and use blood sugar for energy. IR is an important cause of T2D [74] and also has profound effects on lipoproteins, such as LDL [75]. When investigating the causal effect of LDL on T2D, the selected candidate instrumental SNPs may include IR-associated SNPs. These IR-associated SNPs are likely to be associated with both LDL and T2D, leading to correlated pleiotropy in the MR analysis. However, METEOR primarily focuses on modeling uncorrelated horizontal pleiotropy, it does not account for correlated horizontal pleiotropy. Statistically, modelling the correlated pleiotropy in addition to uncorrelated pleiotropy may introduces challenges related to the identifiability of causal effects. Several univariate outcome MR methods, such as CAUSE, MRAID and LHC-MR, model both types of horizontal pleiotropy by assuming only a small proportion of SNPs exhibit correlated pleiotropic effects, thereby mitigating identifiability concerns. We conducted additional simulations with correlated horizontal pleiotropy (details in Supplementary Note 16). METEOR maintained type I error control and was robust with modest correlated horizontal pleiotropy but exhibit inflated type I error control when the proportion of pleiotropic SNPs becomes large (Supplementary Fig. S40). It is important to note that distinguishing causal effects from correlated pleiotropy becomes even more challenging in multi-outcome MR settings, as the increased number of outcomes and the complex correlations among them may elevate the likelihood that SNPs may appear to exhibit correlated pleiotropic effects, further complicating causal inference.

**Key Points**

- METEOR addresses the challenge of identifying causal exposures for multiple outcomes underlying multimorbidity by formulating a multi-outcome MR framework that jointly infers shared and outcome-specific exposures.
- METEOR adaptively selects valid IVs from correlated SNPs, accounts for sample overlap and horizontal pleiotropy, and employs a joint likelihood-based procedure to ensure calibrated $P$-values and accurate effect estimates.
- Simulations show that METEOR presents well-calibrated $P$-values and high power for both global and single-outcome tests. In real data, METEOR suggests that controlling BMI may benefit the co-management of multiple CVDs and GI diseases while controlling blood pressure could benefit the co-management of multimorbidity across CVDs and MDs, as well as across GI diseases and MDs.

## Supplementary Material

METEOR-Supp_bbag364

## Data Availability

No data were generated in the present study. The LD score data is publicly available at https://github.com/bulik/ldsc. The UK Biobank individual data is from UK Biobank resource (https://www.ukbiobank.ac.uk/) under application number 30186. The UK Biobank summary data is available at http://www.nealelab.is/uk-biobank/. The FinnGen summary data is available at https://www.finngen.fi/en. 1000 Genomes project data (phase3) is available at https://ftp.1000genomes.ebi.ac.uk/vol1/ftp/release/20130502/.

## Code availability

We used the R package METEOR (v.1.0) developed in the present study for data analysis. METEOR is described in the Methods section and deposited at Github (https://yuanzhongshang.github.io/METEOR/). We also performed analysis using IVW-R (v.0.6.0, https://github.com/cran/MendelianRandomization/), MRAID (v.1.0, https://github.com/yuanzhongshang/MRAID), MR-APSS (v.0.2.0, https://github.com/YangLabHKUST/MR-APSS), MR^2^ (v.0.1.1, https://github.com/lb664/MR2), MrDAG (v.0.1.1, https://github.com/lb664/MrDAG) and PLINK (v.1.90b6.13, https://www.cog-genomics.org/plink/1.9).
