# Supplementary material for: METEOR: a data-adaptive Mendelian randomization method for powerful detection of shared and specific exposures underlying multiple outcomes
Source: Brief Bioinform. 2026 Jul 6;27(4):bbag364. doi: 10.1093/bib/bbag364 (PMC13336660; doi:10.1093/bib/bbag364)
Supplement: METEOR-Supp_bbag364 [file meteor-supp_bbag364.docx]

Supplementary Information for

**METEOR: A data-adaptive Mendelian randomization method for powerful detection of shared and specific exposures underlying multiple outcomes**

Liye Zhang^1,2^, Ran Yan^1,2^, Weiming Gong^1,2^, Xiang Zhou^3,4*^, Lu Liu^1,2*^, Zhongshang Yuan^1,2*^

1. Department of Biostatistics, School of Public Health, Cheeloo College of Medicine, Shandong University, Jinan, Shandong 250012, China.

2. Institute for Medical Dataology, Cheeloo College of Medicine, Shandong University, Jinan, Shandong 250012, China.

3. Department of Biostatistics, University of Michigan, Ann Arbor, MI 48109, USA.

4. Center for Statistical Genetics, University of Michigan, Ann Arbor, MI 48109, USA.

*Correspondence: [xzhousph@umich.edu](mailto:), [luliu@sdu.edu.cn](mailto:luliu@sdu.edu.cn), and [yuanzhongshang@sdu.edu.cn](mailto:yuanzhongshang@sdu.edu.cn).

Contents

[Supplementary Notes 4](#_Toc228304078)

[1. METEOR model with outcomes from the same dataset 4](#_Toc228304079)

[2. METEOR model with outcomes from different datasets 6](#_Toc228304080)

[3. METEOR model accounting for sample overlap between exposure and each outcome as well as among outcomes with summary statistics 9](#_Toc228304081)

[4. Estimation of the correlation matrix $\boldsymbol{\Omega}$ from LDSC 10](#_Toc228304082)

[5. Detailed sampling steps of METEOR 10](#_Toc228304083)

[6. Inference procedure 13](#_Toc228304084)

[7. Causal effect identification 14](#_Toc228304085)

[8. Compared methods 15](#_Toc228304086)

[9. Parameter settings for simulations with varying one parameter at a time 16](#_Toc228304087)

[10. Parameter settings for simulations with varying combinations of sample overlap proportions and correlations 16](#_Toc228304088)

[11. Simulation settings for assessing the robustness of METEOR under violations of normal distribution and linear genetic effects 17](#_Toc228304089)

[12. Simulation settings for assessing the contribution of key components in METEOR 17](#_Toc228304090)

[13. Implementation details for type I error control and power evaluation 18](#_Toc228304091)

[14. Details of real data processing 19](#_Toc228304092)

[15. Results of exposure detection analyses 19](#_Toc228304093)

[16. Parameter settings for simulations with correlated horizontal pleiotropy 21](#_Toc228304094)

[Supplementary Figures 22](#_Toc228304095)

[Supplementary Figure 1 22](#_Toc228304096)

[Supplementary Figure 2 22](#_Toc228304097)

[Supplementary Figure 3 23](#_Toc228304098)

[Supplementary Figure 4 24](#_Toc228304099)

[Supplementary Figure 5 25](#_Toc228304100)

[Supplementary Figure 6 26](#_Toc228304101)

[Supplementary Figure 7 27](#_Toc228304102)

[Supplementary Figure 8 28](#_Toc228304103)

[Supplementary Figure 9 29](#_Toc228304104)

[Supplementary Figure 10 30](#_Toc228304105)

[Supplementary Figure 11 31](#_Toc228304106)

[Supplementary Fig 12 32](#_Toc228304107)

[Supplementary Fig 13 33](#_Toc228304108)

[Supplementary Fig 14 33](#_Toc228304109)

[Supplementary Fig 15 33](#_Toc228304110)

[Supplementary Figure 16 34](#_Toc228304111)

[Supplementary Figure 17 35](#_Toc228304112)

[Supplementary Figure 18 36](#_Toc228304113)

[Supplementary Figure 19 37](#_Toc228304114)

[Supplementary Figure 20 38](#_Toc228304115)

[Supplementary Figure 21 39](#_Toc228304116)

[Supplementary Figure 22 39](#_Toc228304117)

[Supplementary Figure 23 40](#_Toc228304118)

[Supplementary Figure 24 40](#_Toc228304119)

[Supplementary Figure 25 41](#_Toc228304120)

[Supplementary Figure 26 41](#_Toc228304121)

[Supplementary Figure 27 42](#_Toc228304122)

[Supplementary Figure 28 43](#_Toc228304123)

[Supplementary Figure 29 44](#_Toc228304124)

[Supplementary Figure 30 45](#_Toc228304125)

[Supplementary Figure 35 49](#_Toc228304126)

[Supplementary Figure 36 50](#_Toc228304127)

[Supplementary Figure 37 50](#_Toc228304128)

[Supplementary Figure 38 51](#_Toc228304129)

[Supplementary Figure 39 51](#_Toc228304130)

[Supplementary Figure 40 52](#_Toc228304131)

[Supplementary Tables 53](#_Toc228304132)

[Supplementary Table 1 Summary of multi-outcome MR methods 53](#_Toc228304133)

[Supplementary Table 2 The parameter settings in the simulations 54](#_Toc228304134)

[Supplementary Table 3 Individual data used in this study 54](#_Toc228304135)

[Supplementary Table 4 Summary data used in this study 55](#_Toc228304136)

[Supplementary Table 5 CPU time (seconds) for different methods 55](#_Toc228304137)

[Supplementary Table 6 Six MR methods and the corresponding software 56](#_Toc228304138)

[Supplementary Table 7 Simulation scenarios in this study 57](#_Toc228304139)

[Supplementary Table 8 Estimates of causal effect and 95% confidence intervals from different MR methods in the positive control analysis 58](#_Toc228304140)

[Supplementary Table 9 $\boldsymbol{p}$-values or PIP of positive control analysis for all MR methods in two sample MR setting 58](#_Toc228304141)

[Supplementary Table 10 $\boldsymbol{p}$-values or PIP of positive control analysis for all MR methods in one sample MR setting 59](#_Toc228304142)

[Supplementary Table 11 $\boldsymbol{p}$-values or PIP of negative control analysis for all MR methods 60](#_Toc228304143)

[Supplementary Table 12 Results of shared exposure detection analysis based on brain-heart axis 61](#_Toc228304144)

[Supplementary Table 13 Results of shared exposure detection analysis based on brain-gut axis 63](#_Toc228304145)

[Supplementary References 65](#_Toc228304146)

# Supplementary Notes

## 1. METEOR model with outcomes from the same dataset

$\boldsymbol{x}=\boldsymbol{G}_{x}\boldsymbol{\beta}+\boldsymbol{\varepsilon}_{x}$ (1)

$\boldsymbol{y}_{k}=\alpha_{k}\boldsymbol{G}_{y}\boldsymbol{\beta}+\boldsymbol{G}_{y}\boldsymbol{\eta}_{k}+\boldsymbol{\varepsilon}_{y_{k}}, k=1,\cdots,K$ (2)

We denote $\boldsymbol{x}$ as an $n_{1}$-vector of the exposure for $n_{1}$ individuals in the exposure GWAS and $\boldsymbol{y}_{k}$ as an $n_{2}$-vector of the $k$-th outcome for $n_{2}$ individuals in the outcome GWAS. There is no sample overlap between exposure and outcome. We initially select $p$ SNPs that are associated with the exposure with a marginal $p$-value below the genome-wide significance threshold of $5\times{10}^{-8}$. These SNPs are likely in linkage disequilibrium (LD) with each other and are utilized as correlated candidate instrumental variables (IVs). We denote $\boldsymbol{G}_{x}$ as an $n_{1}$ by $p$ genotype matrix for these $p$ SNPs in the exposure dataset, and $\boldsymbol{G}_{y}$ as an $n_{2}$ by $p$ genotype matrix for the same $p$ SNPs in the outcome dataset. We scale $\boldsymbol{x}$,$\boldsymbol{y}_{k}$ and each column of the two genotype matrices ($\boldsymbol{G}_{x}$ and $\boldsymbol{G}_{y}$) to have zero mean and unit standard deviation. $\boldsymbol{\beta}$ is a $p$-vector of correlated SNP effect sizes on the exposure; $\alpha_{k}$ is a scalar that represents the causal effect of the exposure on the $k$-th outcome; $\boldsymbol{\eta}_{k}$ is a $p$-vector of horizontal pleiotropic effects on the $k$-th outcome; $\boldsymbol{\varepsilon}_{x}$ is an $n_{1}$-vector of residual errors with each element independently and identically distributed from the same normal distribution $N\left( 0,\sigma_{x}^{2} \right)$; $\boldsymbol{\varepsilon}_{y_{k}}$ is an $n_{2}$-vector of residual error and ($\boldsymbol{\varepsilon}_{y_{1}},\cdots,\boldsymbol{\varepsilon}_{y_{K}}$) following a matrix normal distribution $MN_{n_{2},K}\left( \boldsymbol{0},\mathbf{I}_{n_{2}},\boldsymbol{\Omega} \right)$, where $\mathbf{I}_{n_{2}}$ is an $n_{2}$ by $n_{2}$ identity matrix and $\boldsymbol{\Omega}$ is a $K$ by $K$ symmetric covariance matrix among $K$ different outcomes. Denote $\boldsymbol{y}=\left( \boldsymbol{y}_{1}^{T},\cdots,\boldsymbol{y}_{K}^{T} \right)^{T}$, $\boldsymbol{\alpha}=\left( \alpha_{1},\cdots\alpha_{K} \right)^{T}$, $\boldsymbol{\eta}=\left( \boldsymbol{\eta}_{1}^{T},\cdots, \boldsymbol{\eta}_{K}^{T} \right)^{T}$ and $\boldsymbol{\varepsilon}_{y}=\left( \boldsymbol{\varepsilon}_{y_{1}}^{T},\cdots,\boldsymbol{\varepsilon}_{y_{K}}^{T} \right)^{T}$. We can rewrite the equation (2) as follow:

$\boldsymbol{y}=\mathbf{I}_{K}\otimes\left( \boldsymbol{G}_{y}\boldsymbol{\beta} \right)\boldsymbol{\alpha}+\mathbf{I}_{K}\otimes\boldsymbol{G}_{y}\boldsymbol{\eta}+\boldsymbol{\varepsilon}_{y}$ (3)

where $\mathbf{I}_{K}$ is a $K$ by $K$ identity matrix and $\boldsymbol{\varepsilon}_{y}$ is an $n_{2}\times K$-vector of residual errors, following a multivariate normal distribution $MVN(\boldsymbol{0},\boldsymbol{\Omega}\otimes\mathbf{I}_{n_{2}})$, $\otimes$ denotes Kronecker product. For $k=1,\cdots,K$ and $j=1,\cdots,p$, we have

$\beta_{j}\sim\pi_{\beta}N\left( 0, \sigma_{\beta}^{2} \right)+\left( 1-\pi_{\beta} \right)\delta_{0}$,

$\eta_{k,j}\sim\pi_{1k}N\left( 0, \sigma_{k}^{2} \right)+\left( 1-\pi_{1k} \right)\delta_{0}$, if $\beta_{j}\neq0$,

$\eta_{k,j}\sim\pi_{0k}N\left( 0, \sigma_{k}^{2} \right)+\left( 1-\pi_{0k} \right)\delta_{0}$, if $\beta_{j}=0$.

where $\delta_{0}$ is Dirac function that represents a point mass at zero. We assume $\sigma_{\beta}^{2}$ follows an inverse gamma distribution $\sigma_{\beta}^{2}\sim InvG\left( a_{\beta},b_{\beta} \right)$, and set $a_{\beta}=\frac{p}{10}+1$ and$b_{\beta}=0.2$ to ensure a prior mean of $\frac{2}{p}$. Similarly, we assume $\sigma_{k}^{2}\sim InvG\left( a_{k},b_{k} \right)$, with $a_{k}=\frac{p}{5}+1$ and $b_{k}=0.2$ that ensure a prior mean of $\frac{1}{p}$. We assume the proportion parameters to follow Beta distributions: $\pi_{\beta}\sim B\mathrm{eta}\left( \lambda_{\beta1},\lambda_{\beta2} \right)$, with $\lambda_{\beta1}=0.5$ and $\lambda_{\beta2}=4.5$ to ensure a prior mean of approximately 0.1; $\pi_{1k}\sim B\mathrm{eta}\left( \lambda_{21k},\lambda_{22k} \right)$ with $\lambda_{21k}=0.5$ and $\lambda_{22k}=1.5$ to ensure a prior mean of 0.25; and $\pi_{0k}\sim B\mathrm{eta}\left( \lambda_{31k},\lambda_{32k} \right)$ with $\lambda_{31k}=0.05$ and $\lambda_{32k}=9.95$ to ensure a prior mean of 0.005. These prior means represent the prior belief that a relatively small proportion of SNPs display non-zero effects on the exposure and horizontal pleiotropy, and that the selected instrumental SNPs ($\beta_{j}\neq0$) are more likely to display horizontal pleiotropy than the non-selected SNPs ($\beta_{j}=0$). Specifically, for the other hyper-parameters, we use relatively non-informative priors, where we set $\alpha_{k}\sim N(0,\sigma_{0k}^{2})$ with $\sigma_{0k}^{2}\to\infty$ for the causal effect parameter $\alpha_{k}$. Of note, our model assumptions on $\boldsymbol{\beta}$ disentangle the putatively SNP-exposure associations from the potentially false signals, while assumptions on $\boldsymbol{\eta}_{k}$ do not necessarily require that the candidate instrumental SNPs affect any of the outcomes only through the exposure.

To facilitate computation, we introduce a $p$-vector of binary indicators $\boldsymbol{\gamma}=\left( \gamma_{1},\ldots,\gamma_{p} \right)^{T}$to indicate whether each SNP has a non-zero effecton the exposure ${(\gamma}_{j}=1)$ or not ${(\gamma}_{j}=0)$. We also introduce another $p$-vector of binary indicators $\boldsymbol{\tau}_{k}=\left( \tau_{k,1},\ldots,\tau_{k,p} \right)^{T}$to indicate whether each SNP has the horizontal pleiotropic effect ${(\tau}_{k,j}=1)$ or not ${(\tau}_{k,j}=0)$ for $k$-th outcome, and $\boldsymbol{\tau}=\left( \boldsymbol{\tau}_{1}^{T},\cdots,\boldsymbol{\tau}_{K}^{T} \right)^{T}$. Thus, we have $p( \gamma_{j}=1)=\pi_{\beta}$, $p\left( \tau_{k,j}=1|\gamma_{j}=1 \right)=\pi_{1k}$ and $p\left( \tau_{k,j}=1|\gamma_{j}=0 \right)=\pi_{0k}$.

The model for summary statistics can be presented in the form of z-scores and the LD matrix. We denote the LD structure of the instrumental SNPs as $\boldsymbol{\Sigma}_{x}$ in the exposure GWAS dataset, and $\boldsymbol{\Sigma}_{y}$ in the outcome GWAS dataset. Often, $\boldsymbol{\Sigma}_{x}$ and $\boldsymbol{\Sigma}_{y}$ are from the same LD reference panels (e.g., the individuals with same ancestry from the 1,000 Genomes project^1^). The proposed model for summary statistics can be constructed as the following two equations

$\boldsymbol{z}_{x}=\sqrt{n_{1}-1}\boldsymbol{\Sigma}_{x}\boldsymbol{\beta}+\boldsymbol{\epsilon}_{x}$ (4)

$\boldsymbol{z}_{y_{k}}=\sqrt{n_{2}-1}\boldsymbol{\Sigma}_{y}\boldsymbol{\beta}\alpha_{k}+\sqrt{n_{2}-1}\boldsymbol{\Sigma}_{y}\boldsymbol{\eta}_{k}+\boldsymbol{\epsilon}_{y_{k}}, k=1,\cdots,K$ (5)

where $\boldsymbol{z}_{x}$ is a $p$-vector of marginal z-scores with $n_{1}$ individuals in the exposure GWAS; $\boldsymbol{z}_{y_{k}}$ is a $p$-vector of marginal z-scores with $n_{2}$ individuals in the $k$-th outcome GWAS; $\boldsymbol{\epsilon}_{x}$ is a $p$-vector of residual error following a multivariate normal distribution $N(\boldsymbol{0},\sigma_{x}^{2}\boldsymbol{\Sigma}_{x})$; $\boldsymbol{\epsilon}_{y_{k}}$ is a $p$-vector of residual error and ($\boldsymbol{\epsilon}_{y_{1}},\cdots,\boldsymbol{\epsilon}_{y_{k}}$) following a matrix normal distribution $MN_{p,k}\left( \boldsymbol{0},\boldsymbol{\Sigma}_{y},\boldsymbol{\Omega} \right)$. Denote $\boldsymbol{z}_{\boldsymbol{y}}=\left( {\boldsymbol{z}_{y}}_{1}^{T},\cdots,{\boldsymbol{z}_{y}}_{k}^{T} \right)^{T}$, and $\boldsymbol{\epsilon}_{y}=\left( \boldsymbol{\epsilon}_{y_{1}}^{T},\cdots,\boldsymbol{\epsilon}_{y_{k}}^{T} \right)^{T}$. We can rewrite the equation (5) as follows:

$\boldsymbol{z}_{y}=\sqrt{n_{2}-1}\left[ \mathbf{I}_{k}\otimes\left( \boldsymbol{\Sigma}_{y}\boldsymbol{\beta} \right)\boldsymbol{\alpha}+\mathbf{I}_{k}\otimes\boldsymbol{\Sigma}_{y}\boldsymbol{\eta} \right]+\boldsymbol{\epsilon}_{y}$ (6)

where $\boldsymbol{\epsilon}_{y}$ is a $p\times k$ matrix of residual errors, following a multivariate normal distribution $MVN(\boldsymbol{0},\boldsymbol{\Omega}\otimes\boldsymbol{\Sigma}_{y})$.

## 2. METEOR model with outcomes from different datasets

When outcomes are from different datasets, the sample sizes and genotype matrices are different. The formula (2) can be constructed as the following equation

$\boldsymbol{y}_{k}=\alpha_{k}\boldsymbol{G}_{y_{k}}\boldsymbol{\beta}+\boldsymbol{G}_{y_{k}}\boldsymbol{\eta}_{k}+\boldsymbol{\varepsilon}_{y_{k}}, k=1,\cdots,K$ (7)

where $\boldsymbol{y}_{k}$ is an $n_{2k}$-vector of the $k$-th outcome for $n_{2k}$ individuals in the $k$-th outcome GWAS. $\boldsymbol{G}_{y_{k}}$ is an $n_{2k}$ by $p$ genotype matrix for the selected $p$ SNPs in the $k$-th outcome dataset.

Without loss of generality, for any $k_{1}$-th and $k_{2}$-th outcomes ($k_{1},k_{2}\in[1,\cdots,K]$), we assume the first $n_{s}$ individuals as the common sample of $k_{1}$-th and $k_{2}$-th outcomes,

$\boldsymbol{y}_{k_{1}}=\alpha_{k_{1}}\boldsymbol{G}_{y_{k_{1}}}\boldsymbol{\beta}+\boldsymbol{G}_{y_{k_{1}}}\boldsymbol{\eta}_{k_{1}}+\boldsymbol{\varepsilon}_{y_{k_{1}}}$ (8)

$\boldsymbol{y}_{k_{2}}=\alpha_{k_{2}}\boldsymbol{G}_{y_{k_{2}}}\boldsymbol{\beta}+\boldsymbol{G}_{y_{k_{2}}}\boldsymbol{\eta}_{k_{2}}+\boldsymbol{\varepsilon}_{y_{k_{2}}}$ (9)

where $\boldsymbol{G}_{y_{k_{1}}}=\left( \begin{matrix} \boldsymbol{G}_{y_{k_{1}s}} \\ \boldsymbol{G}_{y_{k_{1}u}} \end{matrix} \right)$ is an $n_{{2k}_{1}}$ by $p$ genotype matrix for the $p$ SNPs in the $k_{1}$-th outcome dataset and $\boldsymbol{G}_{y_{k_{2}}}=\left( \begin{matrix} \boldsymbol{G}_{y_{k_{2}s}} \\ \boldsymbol{G}_{y_{k_{2}u}} \end{matrix} \right)$ is an $n_{2k_{2}}$ by $p$ genotype matrix for the same $p$ SNPs in the $k_{2}$-th outcome dataset, and $\boldsymbol{G}_{y_{k_{1}s}}=\boldsymbol{G}_{y_{k_{2}s}}$ is $n_{s} \times p$ genotype matrix for the $n_{s}$ overlapped individuals, $\boldsymbol{G}_{y_{k_{1}u}}$ is $\left( n_{{2k}_{1}}-n_{s} \right)\times p$ genotype matrix for individuals only in the $k_{1}$-th outcome GWAS, and $\boldsymbol{G}_{y_{k_{2}u}}$ is $\left( n_{2k_{2}}-n_{s} \right)\times p$ genotype matrix for individuals only in the $k_{2}$-th outcome GWAS. $\boldsymbol{\varepsilon}_{y_{k_{1}}}=\left( \begin{matrix} \boldsymbol{\varepsilon}_{y_{k_{1}s}} \\ \boldsymbol{\varepsilon}_{y_{k_{1}u}} \end{matrix} \right)$ and$\boldsymbol{\varepsilon}_{y_{k_{2}}}=\left( \begin{matrix} \boldsymbol{\varepsilon}_{y_{k_{2}s}} \\ \boldsymbol{\varepsilon}_{y_{k_{2}u}} \end{matrix} \right)$ are $n_{{2k}_{1}}$ and $n_{2k_{2}}$-vectors of residual errors, respectively. Each element of the residual errors for those individuals shared in the $k_{1}$-th and $k_{2}$-th outcomes follows a binary normal distribution $MVN\left( \left( \begin{matrix} 0 \\ 0 \end{matrix} \right),\left( \begin{matrix} \sigma_{y_{k_{1}}}^{2} & \rho_{ek_{1},k_{2}} \sigma_{y_{k_{1}}}\sigma_{y_{k_{2}}} \\ \rho_{ek_{1},k_{2}}\sigma_{y_{k_{1}}}\sigma_{y_{k_{2}}} & \sigma_{y_{k_{2}}}^{2} \end{matrix} \right) \right)$, where $\rho_{ek_{1},k_{2}}$ is the correlation coefficient. Each residual error element for nonoverlapped individuals in $k_{1}$-th or $k_{2}$-th outcome independently follows univariate normal distribution $N\left( 0,\sigma_{y_{k_{1}}}^{2} \right)$ or $N\left( 0,\sigma_{y_{k_{2}}}^{2} \right)$, respectively. Based on (8)-(9), we have

$\boldsymbol{z}_{y_{k_{1}}}=\sqrt{n_{2k_{1}}-1}\boldsymbol{\Sigma}_{y_{k_{1}}}\left( \boldsymbol{\beta}\alpha_{k_{1}}+\boldsymbol{\eta}_{k_{1}} \right)+\frac{{\boldsymbol{G}_{y_{k_{1}}}^{T}\boldsymbol{\varepsilon}}_{y_{k_{1}}}}{\sqrt{n_{2k_{1}}-1}}$ (10)

$\boldsymbol{z}_{y_{k_{2}}}=\sqrt{n_{2k_{2}}-1}\boldsymbol{\Sigma}_{y_{k_{2}}}\left( \boldsymbol{\beta}\alpha_{k_{2}}+\boldsymbol{\eta}_{k_{2}} \right)+\frac{{\boldsymbol{G}_{y_{k_{2}}}^{T}\boldsymbol{\varepsilon}}_{y_{k_{2}}}}{\sqrt{n_{2k_{2}}-1}}$ (11)

Given $\theta=(\boldsymbol{\beta,}\alpha_{k_{1}},\alpha_{k_{2}}\boldsymbol{,}\boldsymbol{\eta}_{k_{1}},\boldsymbol{\eta}_{k_{2}})$, we firstly derive the expectations of $\boldsymbol{z}_{y_{k_{1}}}$ and $\boldsymbol{z}_{y_{k_{2}}}$ based on (10)-(11)

$E[\boldsymbol{z}_{y_{k_{1}}}]=\sqrt{n_{2k_{1}}-1}\boldsymbol{\Sigma}_{y_{k_{1}}}\left( \boldsymbol{\beta}\alpha_{k_{1}}+\boldsymbol{\eta}_{k_{1}} \right)$ (12)

$E[\boldsymbol{z}_{y_{k_{2}}}]=\sqrt{n_{2k_{2}}-1}\boldsymbol{\Sigma}_{y_{k_{2}}}\left( \boldsymbol{\beta}\alpha_{k_{2}}+\boldsymbol{\eta}_{k_{2}} \right)$ (13)

Then, we derive the variances of $\boldsymbol{z}_{y_{k_{1}}}$ and $\boldsymbol{z}_{y_{k_{2}}}$, as well as the covariance of $\boldsymbol{z}_{y_{k_{1}}}$ and $\boldsymbol{z}_{y_{k_{2}}}$:

$var[\boldsymbol{z}_{y_{k_{1}}}]=var\left[ \frac{{\boldsymbol{G}_{y_{k_{1}}}^{T}\boldsymbol{\varepsilon}}_{y_{k_{1}}}}{\sqrt{n_{2k_{1}}-1}} \right]=\sigma_{y_{k_{1}}}^{2}\boldsymbol{\Sigma}_{y_{k_{1}}}$ (14)

$var[\boldsymbol{z}_{y_{k_{2}}}]=var\left[ \frac{{\boldsymbol{G}_{y_{k_{2}}}^{T}\boldsymbol{\varepsilon}}_{y_{k_{2}}}}{\sqrt{n_{2k_{2}}-1}} \right]=\sigma_{y_{k_{2}}}^{2}\boldsymbol{\Sigma}_{{y_{k}}_{2}}$ (15)

$$Cov\left[ \boldsymbol{z}_{y_{k_{1}}},\boldsymbol{z}_{y_{k_{2}}} \right]=E\left[ \left( \boldsymbol{z}_{y_{k_{1}}}-E\left[ \boldsymbol{z}_{y_{k_{1}}} \right] \right)\left( \boldsymbol{z}_{y_{k_{2}}}-E\left[ \boldsymbol{z}_{y_{k_{2}}} \right] \right)^{T} \right]=E\left[ \frac{{\boldsymbol{G}_{y_{k_{1}}}^{T}\boldsymbol{\varepsilon}}_{y_{k_{1}}}}{\sqrt{n_{2k_{1}}-1}}\cdot\left( \frac{{\boldsymbol{G}_{y_{k_{2}}}^{T}\boldsymbol{\varepsilon}}_{y_{k_{2}}}}{\sqrt{n_{2k_{2}}-1}} \right)^{T} \right]=E\left[ \frac{{\boldsymbol{G}_{y_{k_{1}}}^{T}\boldsymbol{\varepsilon}}_{y_{k_{1}}}\boldsymbol{\varepsilon}_{y_{k_{2}}}^{T}\boldsymbol{G}_{y_{k_{2}}}}{\sqrt{(n_{2k_{1}}-1)(n_{2k_{2}}-1)}} \right]=\frac{1}{\sqrt{(n_{2k_{1}}-1)(n_{2k_{2}}-1)}}\boldsymbol{G}_{y_{k_{1}}}^{T}E\left[ \boldsymbol{\varepsilon}_{y_{k_{1}}} \boldsymbol{\varepsilon}_{y_{k_{2}}}^{T} \right]\boldsymbol{G}_{y_{k_{2}}}=\frac{1}{\sqrt{\left( n_{2k_{1}}-1 \right)\left( n_{2k_{2}}-1 \right)}}\left( \boldsymbol{G}_{y_{k_{1}s}}^{T},\boldsymbol{G}_{y_{k_{1}u}}^{T} \right)E\left[ \left( \begin{matrix} \boldsymbol{\varepsilon}_{y_{k_{1}s}} \\ \boldsymbol{\varepsilon}_{y_{k_{1}u}} \end{matrix} \right)\left( \begin{matrix} \boldsymbol{\varepsilon}_{y_{k_{2}s}}^{T} & \boldsymbol{\varepsilon}_{y_{k_{2}u}}^{T} \end{matrix} \right) \right]\left( \begin{matrix} \boldsymbol{G}_{y_{k_{2}s}} \\ \boldsymbol{G}_{y_{k_{2}u}} \end{matrix} \right)=\frac{1}{\sqrt{\left( n_{2k_{1}}-1 \right)\left( n_{2k_{2}}-1 \right)}}\left( \boldsymbol{G}_{y_{k_{1}s}}^{T},\boldsymbol{G}_{y_{k_{1}u}}^{T} \right)\left( \begin{matrix} \rho_{ek_{1},k_{2}}\sigma_{y_{k_{1}}}\sigma_{y_{k_{2}}}\mathbf{I}_{s\times s} & \boldsymbol{0} \\ \boldsymbol{0} & \boldsymbol{0} \end{matrix} \right)\left( \begin{matrix} \boldsymbol{G}_{y_{k_{2}s}} \\ \boldsymbol{G}_{y_{k_{2}u}} \end{matrix} \right)=\frac{1}{\sqrt{\left( n_{2k_{1}}-1 \right)\left( n_{2k_{2}}-1 \right)}}\boldsymbol{G}_{y_{k_{1}s}}^{T}\rho_{ek_{1},k_{2}}\sigma_{y_{k_{1}}}\sigma_{y_{k_{2}}}\boldsymbol{G}_{y_{k_{2}s}}=\frac{(n_{s}-1)\rho_{ek_{1},k_{2}}\sigma_{y_{k_{1}}}\sigma_{y_{k_{2}}}}{\sqrt{\left( n_{2k_{1}}-1 \right)\left( n_{2k_{2}}-1 \right)}}\boldsymbol{\Sigma}_{s}=\rho_{k_{1},k_{2}}\sigma_{y_{k_{1}}}\sigma_{y_{k_{2}}}\boldsymbol{\Sigma}_{s}$$

(16)

where $\mathbf{I}_{s\times s}$ is $s\times s$ identity matrix and $\rho_{k_{1},k_{2}} =\frac{(n_{s}-1)\rho_{ek_{1},k_{2}}}{\sqrt{\left( n_{1}-1 \right)\left( n_{2}-1 \right)}}$ is the intercept estimated from bivariate LDSC, which can account for the correlation of the two outcomes. Based on (14)-(16), we obtain

$Cov\left( \begin{matrix} \boldsymbol{z}_{y_{k_{1}}} \\ \boldsymbol{z}_{y_{k_{2}}} \end{matrix} \right)=\left( \begin{matrix} \sigma_{y_{k_{1}}}^{2}\boldsymbol{\Sigma}_{{y_{k}}_{1}} & \rho_{k_{1},k_{2}}\sigma_{y_{k_{1}}}\sigma_{y_{k_{2}}}\boldsymbol{\Sigma}_{s} \\ \rho_{k_{1},k_{2}}\sigma_{y_{k_{1}}}\sigma_{y_{k_{2}}}\boldsymbol{\Sigma}_{s} & \sigma_{y_{k_{2}}}^{2}\boldsymbol{\Sigma}_{{y_{k}}_{2}} \end{matrix} \right)$ (17)

Assume $\boldsymbol{\Sigma}_{{y_{k}}_{1}}\boldsymbol{=}\boldsymbol{\Sigma}_{{y_{k}}_{2}}\boldsymbol{=}\boldsymbol{\Sigma}_{s}=\boldsymbol{\Sigma}_{y}$, and denote $\boldsymbol{\Omega}\boldsymbol{=}\left( \begin{matrix} \sigma_{y_{k_{1}}}^{2} & \rho_{k_{1},k_{2}}\sigma_{y_{k_{1}}}\sigma_{y_{k_{2}}} \\ \rho_{k_{1},k_{2}}\sigma_{y_{k_{1}}}\sigma_{y_{k_{2}}} & \sigma_{y_{k_{2}}}^{2} \end{matrix} \right)$, we obtain the $\left( \boldsymbol{z}_{y_{k_{1}}}^{T},\boldsymbol{z}_{y_{k_{2}}}^{T} \right)^{T}$ follow the multivariate normal distribution

$\left( \begin{matrix} \boldsymbol{z}_{y_{k_{1}}} \\ \boldsymbol{z}_{y_{k_{2}}} \end{matrix} \right)\sim MVN\left( \left( \begin{matrix} E[\boldsymbol{z}_{y_{k_{1}}}] \\ E[\boldsymbol{z}_{y_{k_{2}}}] \end{matrix} \right),\boldsymbol{\Omega}\boldsymbol{\otimes}\boldsymbol{\Sigma}_{y} \right)$ (18)

which can be further converted to a matrix normal distribution

$\left( \boldsymbol{z}_{y_{k_{1}}}\boldsymbol{,}\boldsymbol{z}_{y_{k_{2}}} \right)\sim MN\left( \left( E[\boldsymbol{z}_{y_{k_{1}}}],E[\boldsymbol{z}_{y_{k_{2}}}] \right),\boldsymbol{\Sigma}_{y},\boldsymbol{\Omega} \right)$ (19)

Furthermore, we obtain the general formula for the $k$ outcomes,

$\boldsymbol{z}_{y_{k}}=\sqrt{n_{2k}-1}\boldsymbol{\Sigma}_{y_{k}}\boldsymbol{\beta}\alpha_{k}+\sqrt{n_{2k}-1}\boldsymbol{\Sigma}_{y_{k}}\boldsymbol{\eta}_{k}+\boldsymbol{\epsilon}_{y_{k}}, k=1,\cdots,K$ (20)

where $\boldsymbol{z}_{y_{k}}$ is a $p$-vector of marginal z-scores with $n_{2k}$ individuals in the $k$-th outcome GWAS; $\boldsymbol{\Sigma}_{y_{k}}$ is a $p\times p$ symmetric positive definite matrix. Often, $\boldsymbol{\Sigma}_{y_{k}}$ is from the same LD reference panels (e.g., the individuals with same ancestry from the 1,000 Genomes project), thus, we can obtain $\boldsymbol{\Sigma}_{y_{1}}=\cdots\boldsymbol{\Sigma}_{y_{K}}=\boldsymbol{\Sigma}_{y}$. In addition, we denote $\boldsymbol{\xi}=\left( \sqrt{n_{21}-1},\cdots,\sqrt{n_{2K}-1} \right)^{T}$. We can rewrite the equations (20) as follows:

$\boldsymbol{z}_{y}=\mathbf{I}_{K}\otimes\left( \boldsymbol{\Sigma}_{y}\boldsymbol{\beta} \right)\left( \boldsymbol{\xi\circ\alpha} \right)+\mathbf{I}_{K}\otimes\boldsymbol{\Sigma}_{y}\left( \left( \boldsymbol{\xi\otimes}\boldsymbol{1}_{p} \right)\boldsymbol{\circ\eta} \right)+\boldsymbol{\epsilon}_{y}$ (21)

where $\boldsymbol{1}_{p}$ is a $p$-vector of ones, and which can be further converted to a matrix normal distribution

$\boldsymbol{Z}_{Y}^{T}\sim MN\left( \left( E\left[ \boldsymbol{z}_{y_{1}} \right],E\left[ \boldsymbol{z}_{y_{2}} \right],\cdots,E\left[ \boldsymbol{z}_{y_{K}} \right] \right),\boldsymbol{\Sigma}_{y},\boldsymbol{\Omega} \right)$ (22)

$\boldsymbol{\Omega}\boldsymbol{=}\left( \begin{matrix} \sigma_{y_{1}}^{2} & \rho_{1,2}\sigma_{y_{1}}\sigma_{y_{2}} & \cdots& \rho_{1,K}\sigma_{y_{1}}\sigma_{y_{K}} \\ \rho_{2,1}\sigma_{y_{2}}\sigma_{y_{1}} & \sigma_{y_{2}}^{2} & \boldsymbol{\cdots} & \rho_{2,K}\sigma_{y_{2}}\sigma_{y_{K}} \\ \boldsymbol{\cdots} & \boldsymbol{\cdots} & \boldsymbol{\cdots} & \boldsymbol{\cdots} \\ \rho_{K,1}\sigma_{y_{K}}\sigma_{y_{1}} & \rho_{K,2}\sigma_{y_{K}}\sigma_{y_{2}} & \boldsymbol{\cdots} & \sigma_{y_{K}}^{2} \end{matrix} \right)$ (23)

where $\boldsymbol{Z}_{Y}=\left( \boldsymbol{z}_{y_{1}}, \cdots,\boldsymbol{z}_{y_{K}} \right)^{T}$; the $p$ by $p$ row covariance matrix $\boldsymbol{\Sigma}_{y}$ characterizes the covariance among the marginal z-scores across SNPs due to LD; and the $K$ by $K$ column covariance matrix $\boldsymbol{\Omega}$ characterizes the covariance among the marginal z-scores on the $K$ outcomes to account for the correlations due to potential sample overlap.

In particular, when $K$ outcomes are from the same dataset, $\rho_{k_{1},k_{2}}=\rho_{ek_{1},k_{2}}$, we obtain $\boldsymbol{\Omega}\boldsymbol{=}Cov\left( \boldsymbol{y}_{1},\cdots\boldsymbol{y}_{K} \right)=Cov\left( \boldsymbol{z}_{1},\cdots\boldsymbol{z}_{K} \right)$. In addition, $\boldsymbol{\Omega}\boldsymbol{=}\left( \sigma_{y_{k}}^{2} \right)_{K\times K}$ is a diagonal matrix with diagonal elements are $\sigma_{y_{k}}^{2}$, $k\in[1,\cdots,K]$, when no overlapping individuals between any two outcome datasets.

## 3. METEOR model accounting for sample overlap between exposure and each outcome as well as among outcomes with summary statistics

For the exposure dataset and the $K$ outcome datasets, we consider the possibility of sample overlap between any two datasets. Based on formula (4) and (20), we obtain

$\left( \boldsymbol{z}_{x},\boldsymbol{z}_{y_{1}},\cdots,\boldsymbol{z}_{y_{K}} \right)\sim MN\left( \left( E\left[ \boldsymbol{z}_{x} \right],E\left[ \boldsymbol{z}_{y_{1}} \right],\cdots,E\left[ \boldsymbol{z}_{y_{K}} \right] \right),\boldsymbol{\Sigma},\boldsymbol{\Omega} \right)$ (24)

$E\left[ \boldsymbol{z}_{x} \right]=\sqrt{n_{1}-1}\boldsymbol{\Sigma}\boldsymbol{\beta}$ (25)

$E\left[ \boldsymbol{z}_{y_{k}} \right]=\sqrt{n_{2k}-1}\boldsymbol{\Sigma}\boldsymbol{\beta}\alpha_{k}+\sqrt{n_{2k}-1}\boldsymbol{\Sigma}\boldsymbol{\eta}_{k},k=1,\cdots,K$ (26)

$\boldsymbol{\Omega}\boldsymbol{=}\left( \begin{matrix} \sigma_{x}^{2} & \rho_{x,y_{1}}\sigma_{x}\sigma_{y_{1}} & \cdots& \rho_{x,y_{K}}\sigma_{x}\sigma_{y_{K}} \\ \rho_{y_{1},x}\sigma_{y_{1}}\sigma_{x} & \sigma_{y_{1}}^{2} & \cdots& \rho_{y_{1},y_{K}}\sigma_{y_{1}}\sigma_{y_{K}} \\ \cdots& \cdots& \cdots& \cdots\\ \rho_{y_{K},x}\sigma_{y_{K}}\sigma_{x} & \rho_{y_{K},y_{1}}\sigma_{y_{K}}\sigma_{y_{1}} & \cdots& \sigma_{y_{K}}^{2} \end{matrix} \right)$ (27)

Again, the $p$ by $p$ covariance matrix $\boldsymbol{\Sigma}$ characterizes the covariance among the marginal z-scores across SNPs due to LD, and the $K+1$ by $K+1$ covariance matrix $\boldsymbol{\Omega}$ characterizes the covariance among the marginal z-scores on the exposure and $K$ outcomes to account for the correlations due to potential sample overlap.

Denote $\boldsymbol{z}=\left( \boldsymbol{z}_{x}^{T},\boldsymbol{z}_{y_{1}}^{T},\cdots,\boldsymbol{z}_{y_{K}}^{T} \right)^{T}$, $\boldsymbol{\xi}=\left( \sqrt{n_{1}-1},\sqrt{n_{21}-1},\cdots,\sqrt{n_{2K}-1} \right)^{T}$, $\boldsymbol{\alpha}=\left( 1,\alpha_{1}, \cdots,\alpha_{K} \right)^{T}$, $\boldsymbol{\eta}=\left( \boldsymbol{\eta}_{0}^{T},\boldsymbol{\eta}_{1}^{T},\cdots, \boldsymbol{\eta}_{K}^{T} \right)^{T}$, $\boldsymbol{\epsilon}=\left( \boldsymbol{\epsilon}_{x}^{T},\boldsymbol{\epsilon}_{y_{1}}^{T},\cdots,\boldsymbol{\epsilon}_{y_{K}}^{T} \right)^{T}$, where $\boldsymbol{\eta}_{0}$ is a $p$-vector of zeros. We can rewrite the equation (24) as follows:

$\boldsymbol{z}=\mathbf{I}_{K+1}\otimes\left( \boldsymbol{\Sigma}\boldsymbol{\beta} \right)\left( \boldsymbol{\xi\circ\alpha} \right)+\mathbf{I}_{K+1}\otimes\boldsymbol{\Sigma}\left( \left( \boldsymbol{\xi\otimes}\boldsymbol{1}_{p} \right)\boldsymbol{\circ\eta} \right)+\boldsymbol{\epsilon}$ (28)

$\boldsymbol{z}\sim MVN(\boldsymbol{E[z]},\boldsymbol{\Omega}\otimes\boldsymbol{\Sigma})$ (29)

$\mathbf{E}\left[ \boldsymbol{z} \right]\boldsymbol{=}\left( E\left[ \boldsymbol{z}_{x} \right]^{T},E\left[ \boldsymbol{z}_{y_{1}} \right]^{T},\cdots,E\left[ \boldsymbol{z}_{y_{K}} \right]^{T} \right)^{T}$ (30)

where $\mathbf{I}_{K+1}$ is a $K+1$ by $K+1$ identify matrix, and the term $\left( \boldsymbol{\xi\circ\alpha} \right)$ represents Hadamard product, also known as element wise product, of the two vectors $\boldsymbol{\xi}$ and $\boldsymbol{\alpha}$.

## 4. Estimation of the correlation matrix $\boldsymbol{\Omega}$ from LDSC

We adopt the linkage disequilibrium score regression (LDSC) method, the intercept of which can be used to estimate correction factor, in order to obtain the estimate $\hat{\boldsymbol{\Omega}}$ of the matrix parameter $\boldsymbol{\Omega}$.

$$\hat{\boldsymbol{\Omega}}\boldsymbol{=}\left( \begin{matrix} \omega_{0} & \omega_{0,1} & \cdots& \omega_{0,K} \\ \omega_{0,1} & \omega_{1} & \cdots& \omega_{1,K} \\ \cdots& \cdots& \cdots& \cdots\\ \omega_{0,K} & \omega_{1,K} & \cdots& \omega_{k} \end{matrix} \right)$$

where $\omega_{0}$ and $\omega_{k}$ ($k=1,\cdots K$) are the intercepts estimated from single-trait LDSC for the exposure and the $k$-th outcome, respectively, which are used to adjust the bias in estimation errors^2^. While $\omega_{0,k}$ is the intercept estimated from bivariate LDSC to account for the correlation of the estimation errors between the exposure and the $k$-th outcome^3, 4^, and $\omega_{k_{1},k_{2}}$ ($k_{1},k_{2}\in[1,\cdots,K]$) is also the intercept estimated from bivariate LDSC to account for the correlation of the estimation errors among different outcomes^3, 4^.

## 5. Detailed sampling steps of METEOR

For the summary statistics version of METEOR model accounting for sample overlap among exposure and outcomes, given $\hat{\boldsymbol{\Omega}}$ obtained by LDSC, denote $\boldsymbol{\pi}_{1}\boldsymbol{=}\left( \pi_{11}\boldsymbol{,\cdots}\pi_{1K} \right)^{T}$, $\boldsymbol{\pi}_{0}\boldsymbol{=}\left( \pi_{01}\boldsymbol{,\cdots}\pi_{0K} \right)^{T}$, $\boldsymbol{\sigma}^{2}=\left( \sigma_{1}^{2},\cdots,\sigma_{K}^{2} \right)^{T}$, we can obtain the posterior distribution for all other parameters as

$f\left( \boldsymbol{\alpha},\boldsymbol{\beta},\boldsymbol{\eta},\boldsymbol{\gamma},\boldsymbol{\tau,}\pi_{\beta},\boldsymbol{\pi}_{1},\boldsymbol{\pi}_{0},\sigma_{\beta}^{2}, \boldsymbol{\sigma}^{2} | \boldsymbol{z} \right)\propto f\left( \boldsymbol{z} | \boldsymbol{\alpha},\boldsymbol{\beta},\boldsymbol{\eta},\boldsymbol{\gamma},\boldsymbol{\tau} \right)f\left( \boldsymbol{\beta} | \boldsymbol{\gamma} \right)f\left( \boldsymbol{\eta} | \boldsymbol{\tau} \right)f\left( \boldsymbol{\tau} | \boldsymbol{\gamma} \right)f\left( \boldsymbol{\gamma} | \pi_{\beta} \right)f\left( \pi_{\beta} \right)\prod_{k=1}^{K} \left[ f\left( \pi_{1k} \right)f\left( \pi_{0k} \right) \right]$

$$=\left( 2\pi\right)^{-\frac{p(K+1)}{2}}\left| \boldsymbol{\Omega}\otimes\boldsymbol{\Sigma} \right|^{-\frac{1}{2}}$$

$\exp\left\{ \begin{aligned} -\frac{1}{2}\left[ \boldsymbol{z}-\mathbf{I}_{K+1}\otimes\left( \boldsymbol{\Sigma}\boldsymbol{\beta} \right)\left( \boldsymbol{\xi\circ\alpha} \right)-\mathbf{I}_{K+1}\otimes\boldsymbol{\Sigma}\left( \left( \boldsymbol{\xi\otimes}\boldsymbol{1}_{p} \right)\boldsymbol{\circ\eta} \right) \right]^{T} \\ \left( \boldsymbol{\Omega}\otimes\boldsymbol{\Sigma} \right)^{-1}\left[ \boldsymbol{z}-\mathbf{I}_{K+1}\otimes\left( \boldsymbol{\Sigma}\boldsymbol{\beta} \right)\left( \boldsymbol{\xi\circ\alpha} \right)-\mathbf{I}_{K+1}\otimes\boldsymbol{\Sigma}\left( \left( \boldsymbol{\xi\otimes}\boldsymbol{1}_{p} \right)\boldsymbol{\circ\eta} \right) \right] \end{aligned} \right\}$

$\cdot\prod_{j=1}^{p} \left[ \left( 2\pi\right)^{-\frac{1}{2}}\left( \sigma_{\beta}^{2} \right)^{-\frac{1}{2}}\exp\left( -\frac{\beta_{j}^{2}}{2\sigma_{\beta}^{2}} \right) \right]^{\gamma_{j}}\prod_{j=1}^{p} \left[ \delta_{0}\left( \beta_{j} \right) \right]^{1-\gamma_{j}}$

$\cdot\prod_{k=1}^{K} \prod_{j=1}^{p} \left[ \left( 2\pi\right)^{-\frac{1}{2}}\left( \sigma_{k}^{2} \right)^{-\frac{1}{2}}\exp\left( -\frac{\eta_{k,j}^{2}}{2\sigma_{k}^{2}} \right) \right]^{\tau_{k,j}}\prod_{k=1}^{K} \prod_{j=1}^{p} \left[ \delta_{0}\left( \eta_{k,j} \right) \right]^{1-\tau_{k,j}}$

$\cdot\prod_{k=1}^{K} \left[ \pi_{1k}^{\sum_{j=1}^{p} \left( \gamma_{j}\tau_{k,j} \right)}\left( 1-\pi_{1k} \right)^{\sum_{j=1}^{p} \left( \gamma_{j}\left( 1-\tau_{k,j} \right) \right)}\pi_{0k}^{\sum_{j=1}^{p} \left( \left( 1-\gamma_{j} \right)\tau_{k,j} \right)}\left( 1-\pi_{0k} \right)^{\sum_{j=1}^{p} \left( \left( 1-\gamma_{j} \right)\left( 1-\tau_{k,j} \right) \right)} \right]\cdot\pi_{\beta}^{\sum_{j=1}^{p} \gamma_{j}}\left( 1-\pi_{\beta} \right)^{\sum_{j=1}^{p} \left( 1-\gamma_{j} \right)}\cdot Beta\left( \pi_{\beta},\lambda_{\beta1},\lambda_{\beta2} \right)$

$\cdot\prod_{k=1}^{K} \left[ Beta\left( \pi_{1i},\lambda_{21i},\lambda_{22i} \right)\cdot Beta\left( \pi_{0i},\lambda_{31i},\lambda_{32i} \right) \right]\cdot InvG\left( \sigma_{\beta}^{2},a_{\beta},b_{\beta} \right)\cdot\sum_{k=1}^{K} [InvG\left( \sigma_{k}^{2},a_{k},b_{k} \right)]$

Based on the above likelihood, we conduct Gibbs sampling on all parameters as $\theta=\left( \beta_{j}, \gamma_{j},\eta_{k,j}, \tau_{k,j}, \alpha_{k},\pi_{\beta},\pi_{1k},\pi_{0k},\sigma_{\beta}^{2}, \sigma_{k}^{2} \right)$. We denote $\mathbf{W}_{k+1}$ is a $(K+1)$ by $(K+1)$ identity matrix $\mathbf{I}_{K+1}$ but with the element in row $k+1$ and column $k+1$ is zero; $\boldsymbol{P}_{2k+1}$ is a $(K+1)$-vector of zeros but the $(k+1)$-th element is one, $k=1,\cdots,K$.

Given $\gamma_{j}$=1 and other parameters, the posterior distribution of $\beta_{j}$ is a normal distribution with mean $\mu_{\beta_{j}}$ and variance $\sigma_{\beta_{j}}^{2}$, where

$$\mu_{\beta_{j}}=\left\{ \left[ \boldsymbol{z}-\mathbf{I}_{K+1}\otimes\left( \boldsymbol{\Sigma}_{-j}\boldsymbol{\beta}_{\boldsymbol{-}j} \right)\left( \boldsymbol{\xi\circ\alpha} \right)-\mathbf{I}_{K+1}\otimes\boldsymbol{\Sigma}\left( \left( \boldsymbol{\xi\bigotimes}\boldsymbol{1}_{p} \right)\boldsymbol{\circ\eta} \right) \right]^{T}\left( \boldsymbol{\Omega}\otimes\boldsymbol{\Sigma} \right)^{-1}\left[ \left( \boldsymbol{I}_{K+1}\otimes\boldsymbol{\Sigma}_{j} \right)\left( \boldsymbol{\xi\circ\alpha} \right) \right] \right\}\sigma_{\beta_{j}}^{2}$$

$$\sigma_{\beta_{j}}^{2}=1/K_{1}$$

where $K_{1}=\left[ \left( \mathbf{I}_{K+1}\otimes\boldsymbol{\Sigma}_{j} \right)\left( \boldsymbol{\xi\circ\alpha} \right) \right]^{T}\left( \boldsymbol{\Omega}\otimes\boldsymbol{\Sigma} \right)^{-1}\left[ \left( \mathbf{I}_{K+1}\otimes\boldsymbol{\Sigma}_{j} \right)\left( \boldsymbol{\xi\circ\alpha} \right) \right]+\frac{1}{\sigma_{\beta}^{2}}$.

Given $\tau_{k,j}$=1 and other parameters, the posterior conditional distribution of $\eta_{k,j}$ is a normal distribution with mean $\mu_{\eta_{k,j}}$ and variance $\sigma_{\eta_{k,j}}^{2}$, where

$$\mu_{\eta_{k,j}}=\sqrt{n_{2k}-1}\left[ \boldsymbol{z}-\mathbf{I}_{K+1}\otimes\left( \boldsymbol{\Sigma}\boldsymbol{\beta} \right)\left( \boldsymbol{\xi\circ\alpha} \right)-\mathbf{W}_{k+1}\otimes\boldsymbol{\Sigma}\left( \left( \boldsymbol{\xi\bigotimes}\boldsymbol{1}_{p} \right)\boldsymbol{\circ\eta} \right)-\sqrt{n_{2k}-1}\boldsymbol{P}_{2k+1}\otimes\left( \boldsymbol{\Sigma}_{-j}\boldsymbol{\eta}_{k,-j} \right) \right]^{T}\left( \boldsymbol{\Omega}\otimes\boldsymbol{\Sigma} \right)^{-1}\left( \boldsymbol{P}_{2K}\otimes\boldsymbol{\Sigma}_{j} \right)\sigma_{\eta_{k,j}}^{2}$$

$$\sigma_{\eta_{k,j}}^{2}=1/K_{2}$$

where $K_{2}={\left( n_{2k}-1 \right)\left( \boldsymbol{P}_{2k+1}\otimes\boldsymbol{\Sigma}_{j} \right)}^{T}\left( \boldsymbol{\Omega}\otimes\boldsymbol{\Sigma} \right)^{-1}\left( \boldsymbol{P}_{2k+1}\otimes\boldsymbol{\Sigma}_{j} \right)+\frac{1}{\sigma_{k}^{2}}$.

After integrating out $\beta_{j}$, we obtain the posterior conditional distribution of $\gamma_{j}$ as

$$p\left( \gamma_{j}=1|\boldsymbol{\beta}_{-j}, \boldsymbol{\eta}, \boldsymbol{\alpha},\sigma_{\beta}^{2},\pi_{\beta},\pi_{1k},\pi_{0k} \right)\propto\exp\left\{ \frac{\mu_{\beta_{j}}^{2}}{2\sigma_{\beta_{j}}^{2}}+0.5\text{log}\left( \sigma_{\beta_{j}}^{2} \right)-0.5\text{log}\left( \sigma_{\beta}^{2} \right)+\text{log}\left( \pi_{\beta} \right)+\sum_{k=1}^{K} \left[ \tau_{k,j}\log\left( \pi_{1k} \right)+\left( 1-\tau_{k,j} \right)\log\left( 1-\pi_{1k} \right) \right] \right\}$$

$$p\left( \gamma_{j}=0|\boldsymbol{\beta}_{-j}, \boldsymbol{\eta}, \boldsymbol{\alpha},\sigma_{\beta}^{2},\pi_{\beta},\pi_{1k},\pi_{0k} \right)\propto\exp\left\{ \text{log}\left( 1-\pi_{\beta} \right)+\sum_{k=1}^{K} \left[ \tau_{k,j}\log\left( \pi_{0k} \right)+(1-\tau_{k,j})log(1-\pi_{0k}) \right] \right\}$$

After integrating out $\eta_{k,j}$, we obtain the posterior conditional distribution of $\tau_{k,j}$ as

$$p\left( \tau_{k,j}=1|\boldsymbol{\eta}_{k,-j}, \boldsymbol{\beta},\boldsymbol{\alpha},\sigma_{k}^{2},\pi_{1k},\pi_{0k} \right)\propto\exp\left[ \frac{\mu_{\eta_{k,j}}^{2}}{2\sigma_{\eta_{k,j}}^{2}}+0.5\text{log}\left( \sigma_{\eta_{k,j}}^{2} \right)-0.5\text{log}\left( \sigma_{k}^{2} \right)+\gamma_{j}\text{log}\left( \pi_{1k} \right)+(1-\gamma_{j})log(\pi_{0k}) \right]$$

$$p\left( \tau_{k,j}=0|\boldsymbol{\eta}_{k,-j}, \boldsymbol{\beta}, \boldsymbol{\alpha},\sigma_{k}^{2},\pi_{1k},\pi_{0k} \right)\propto\exp\left[ \gamma_{j}\text{log}\left( 1-\pi_{1k} \right)+(1-\gamma_{j})log({1-\pi}_{0k}) \right]$$

The posterior conditional distribution of $\alpha_{k}$ is a normal distribution with mean $\mu_{\alpha_{k}}$ and variance $\sigma_{\alpha_{k}}^{2}$, where

$$\mu_{\alpha_{k}}=\sqrt{n_{2k}-1}\left[ \boldsymbol{z}-\mathbf{I}_{K+1}\otimes\boldsymbol{\Sigma}\left( \left( \boldsymbol{\xi\bigotimes}\boldsymbol{1}_{p} \right)\boldsymbol{\circ\eta} \right)-\mathbf{W}_{k+1}\otimes\left( \boldsymbol{\Sigma}\boldsymbol{\beta} \right)\left( \boldsymbol{\xi\circ\alpha} \right) \right]^{T}\left( \boldsymbol{\Omega}\otimes\boldsymbol{\Sigma} \right)^{-1}\left[ \boldsymbol{P}_{2k+1}\otimes\left( \boldsymbol{\Sigma}\boldsymbol{\beta} \right) \right]\sigma_{\alpha_{k}}^{2}$$

$$\sigma_{\alpha_{k}}^{2}=1/K_{3}$$

where $K_{3}=(n_{2k}-1)\left[ \boldsymbol{P}_{2k+1}\otimes\left( \boldsymbol{\Sigma}\boldsymbol{\beta} \right) \right]^{T}\left( \boldsymbol{\Omega}\otimes\boldsymbol{\Sigma} \right)^{-1}\left[ \boldsymbol{P}_{2k+1}\otimes\left( \boldsymbol{\Sigma}\boldsymbol{\beta} \right) \right]$.

The posterior conditional distribution of $\pi_{\beta}$ is $Beta({\sum_{j=1}^{p} \gamma_{j}+\lambda}_{\beta1},{\sum_{j=1}^{p} {(1-\gamma}_{j})+\lambda}_{\beta2})$.

The posterior conditional distribution of $\pi_{1k}$ is $Beta({\sum_{j=1}^{p} {\gamma_{j}\tau}_{k,j}+\lambda}_{21k},{\sum_{j=1}^{p} {\gamma_{j}(1-\tau}_{k,j})+\lambda}_{22k})$.

The posterior conditional distribution of $\pi_{0k}$ is $Beta({\sum_{j=1}^{p} {(1-\gamma_{j})\tau}_{k,j}+\lambda}_{31k},{\sum_{j=1}^{p} {(1-\gamma_{j})(1-\tau}_{k,j})+\lambda}_{32k})$.

The posterior conditional distribution of $\sigma_{\beta}^{2}$is an inverse gamma distribution with the shape parameter $\sigma_{\beta\_shape}^{2}=\frac{\sum_{j=1}^{p} \gamma_{j}}{2}+a_{\beta}$, and the scale parameter $\sigma_{\beta\_scale}^{2}=\frac{\sum_{j=1}^{p} \gamma_{j}\beta_{j}^{2}+2b_{\beta}}{2}$.

The posterior conditional distribution of$\sigma_{k}^{2}$is an inverse gamma distribution with the shape parameter $\sigma_{k\_shape}^{2}=\frac{\sum_{j=1}^{p} \tau_{k,j}}{2}+a_{k}$, and the scale parameter $\sigma_{k\_scale}^{2}=\frac{\sum_{j=1}^{p} \tau_{k,j}\eta_{k,j}^{2}+2b_{k}}{2}$.

## 6. Inference procedure

We denote $\mu_{\alpha_{k}}$ and $\sigma_{\alpha_{k}}^{2}$ as the posterior mean and posterior variance of the causal effect parameter $\alpha_{k}$. Since both the likelihood and the posterior follow normal distributions asymptotically, we, with a normal distribution $N(0,\sigma_{0k}^{2})$ as a prior distribution, are able to obtain the approximate maximum likelihood estimate and its standard error by the method of moment as

$$\hat{\alpha}_{k}=\frac{\sigma_{0k}^{2}\mu_{\alpha k}}{\sigma_{0k}^{2}-\sigma_{\alpha k}^{2}}$$

$$se\left( \hat{\alpha}_{k} \right)=\frac{\sigma_{0k}\sigma_{\alpha k}}{\sqrt{\sigma_{0k}^{2}-\sigma_{\alpha k}^{2}}}$$

The z-score $Z_{k}=\frac{\hat{\alpha}_{k}}{se\left( \hat{\alpha}_{k} \right)}$ follows standard normal distribution asymptotically, which allows us to obtain a corresponding $p$-value for testing the null hypothesis $H_{0}:$ $\alpha_{k}=0$. With a non-informative prior for $\alpha_{k}$ by setting $\sigma_{0k}^{2}\to\infty$, we have $\hat{\alpha}_{k}=\mu_{\alpha k}$ and $se\left( \hat{\alpha}_{k} \right)=\sigma_{\alpha k}$. Note that the choice of the prior on $\alpha_{k}$ dose not substantially influence the results as we only use the likelihood, expressed as the ratio of the posterior and prior, for causal effect test^5, 6^. In another way, the test statistic is

$$T_{Wald_{k}}=\frac{\hat{\alpha}_{k}^{2}}{Var(\hat{\alpha}_{k})}$$

which asymptotically distributed as $\chi_{df=1}^{2}$, and its $p$-value can be obtained accordingly. Similarly, for the multivariate analysis, we can use the Gibbs posterior samples to obtain the estimate ${\hat{\boldsymbol{\alpha}}}^{\boldsymbol{*}}$ of the causal effects $\boldsymbol{\alpha}^{\boldsymbol{*}}\boldsymbol{=}\left( \alpha_{1}, \cdots,\alpha_{K} \right)^{T}$ as well as the estimate $Cov({\hat{\boldsymbol{\alpha}}}^{*})$ of their variance-covariance matrix, and then we can carry out the Wald test to test the overall hypothesis $H_{0}:\alpha_{1}=\cdots=\alpha_{K}=0$. Then, the test statistic is

$$T_{Wald}={\hat{\boldsymbol{\alpha}}}^{*T}\left[ Cov({\hat{\boldsymbol{\alpha}}}^{*}) \right]^{-1}{\hat{\boldsymbol{\alpha}}}^{\boldsymbol{*}}$$

which approximately follows a chi-square distribution with $K$ degrees of freedom under $H_{0}$.

In addition, to test whether $H_{0}:\alpha_{1}=\cdots=\alpha_{K}=0$ (the exposure does not have any effect on any outcome, global test), univariate MR usually applies the minP test with Bonferroni correction. Suppose the $p$-values for the exposure effects on different outcomes are $p_{1},\cdots,p_{K}$, the $p$-value for the overall test is $min({Kp}_{1},\cdots,{Kp}_{K},1)$. It compares $K$ times the smallest $p$-value of any coefficient $\alpha_{k}$ to the significant threshold (e.g., 0.05). This method does not take into account the correlation between different outcomes, which may give conservative results.

## 7. Causal effect identification

The causal interpretation of the parameter vector $\boldsymbol{\alpha}^{\boldsymbol{*}}$ and its identification can be derived under the framework of decision-theoretic causal inference^7, 8, 9, 10^. We define the causal effects of exposure $\boldsymbol{x}$ on the multiple outcomes $\boldsymbol{Y=}\left( \boldsymbol{y}_{1}\boldsymbol{,\cdots,}\boldsymbol{y}_{K} \right)^{T}$ as the difference between the expected values of $\boldsymbol{Y}$ under an intervention that impose on $\boldsymbol{x}$ a reference value $\boldsymbol{x}_{0}$ and another intervention that impose another value $\boldsymbol{x}_{1}$. Let the symbol $F_{x}$ label the regime under which the value of $\boldsymbol{x}$ is generated, with $F_{x}\boldsymbol{=}\boldsymbol{x}_{1}$ indicating that $\boldsymbol{x}$ is fixed to value $\boldsymbol{x}_{1}$ by an intervention, and $F_{x}\boldsymbol{=\emptyset}$ denoting the observational regime under which the data have actually been generated. Then the average causal effects (**ACE**) of $\boldsymbol{x}$ on the $K$ outcomes $\boldsymbol{Y}$ is defined by

$$\boldsymbol{ACE=E}\left( \boldsymbol{Y}^{T} | F_{x}\boldsymbol{=}\boldsymbol{x}_{1} \right)\boldsymbol{-E(}\boldsymbol{Y}^{T}\boldsymbol{|}F_{x}\boldsymbol{=}\boldsymbol{x}_{0}\boldsymbol{)}$$

For instance, for any outcome $k$, $k=1,\cdots,K$, the ACE of $\boldsymbol{x}$ on the continuous outcome $\boldsymbol{Y}_{k}^{T}$ following the definition above can be expressed as

$$\boldsymbol{ACE=E}\left( \boldsymbol{Y}_{k}^{T} | F_{x}\boldsymbol{=}\boldsymbol{x}_{1} \right)\boldsymbol{-E(}\boldsymbol{Y}_{k}^{T}\boldsymbol{|}F_{x}\boldsymbol{=}\boldsymbol{x}_{0}\boldsymbol{)}$$

Let the notation $\boldsymbol{A⫫B|C}$ indicates that $\boldsymbol{A}$ is independent of $\boldsymbol{B}$ given $\boldsymbol{C}$.

Our proposed METEOR model based on the observational data under $F_{x}\boldsymbol{=\emptyset}$ has been presented below. Note that we directly model the horizontal pleiotropic effects through the $\boldsymbol{G}\longrightarrow\boldsymbol{Y}$ arrow. Therefore, our model does not require the Exclusion Restriction condition of traditional MR. However, the other two assumptions in traditional MR must be satisfied; that is, $\boldsymbol{G}$ is associated with $\boldsymbol{x}$, and $\boldsymbol{U}⫫\boldsymbol{G}$ (1).

One must note that requirements relating only to the observational regime can never be sufficient to estimate the causal effect of $\boldsymbol{x}$ on $\boldsymbol{Y}$, which is defined in terms of interventional regimes. Instead, we need to make additional assumptions that relate the observational regime $F_{x}=\emptyset$ to the interventional regimes $F_{x}=\boldsymbol{x}$. Under the assumption that the unobserved $\boldsymbol{U}$ is a sufficient covariate for the effect of $\boldsymbol{x}$ on $\boldsymbol{Y}$, we can do this by elaborating **Figure S1** to explicitly include the nonstochastic regime indicator $F_{x}$ for $\boldsymbol{x}$. For $F_{x}\boldsymbol{=\emptyset}$, this recovers the assumptions embedded in **Figure S1**, but in addition it relates the observational structure to what would happen under an intervention to set $\boldsymbol{x}$.


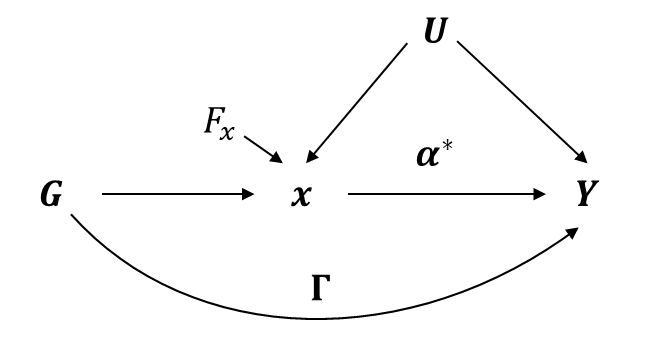


**Figure S1**. The causal diagram of METEOR with the nonstochastic regime indicator $F_{x}$

It illustrates that an intervention on $\boldsymbol{x}$ will not affect $\boldsymbol{G}$ or $\boldsymbol{U}$, that is $F_{x}⫫(\boldsymbol{U},\boldsymbol{G})$ (2). And conditional on $\boldsymbol{G}$ and $\boldsymbol{U}$, the distribution of $\boldsymbol{Y}$ given $\boldsymbol{x}$ does not depend on whether the value of $\boldsymbol{x}$ has been generated by passive observation or intervention, that is $\boldsymbol{Y}⫫F_{x}|\left( \boldsymbol{x},\boldsymbol{G},\boldsymbol{U} \right)$ (3). Furthermore, the formula (**1**) can be extended to $\boldsymbol{U}⫫\boldsymbol{G|}F_{x}$ (4).

We can describe the dependence of $\boldsymbol{Y}$ on $(\boldsymbol{x},\boldsymbol{G})$ by a linear model: $E\left( \boldsymbol{Y}^{T} | \boldsymbol{x},\boldsymbol{G},\boldsymbol{U} \right)=\boldsymbol{M}+\boldsymbol{x}\boldsymbol{\alpha}^{*T}+\boldsymbol{G}\boldsymbol{\Gamma}^{T}$ (5), where $\boldsymbol{\Gamma}=\left( \boldsymbol{\eta}_{1},\cdots,\boldsymbol{\eta}_{K} \right)^{T}$, $\boldsymbol{M}$ is some function of $\boldsymbol{U}$. Because (5) holds in the interventional regime $F_{x}=\boldsymbol{x}_{1}$, we deduce $E\left( \boldsymbol{Y}^{T} | F_{x}=\boldsymbol{x}_{1} \right)=\boldsymbol{M}_{0}+\boldsymbol{x}_{1}\boldsymbol{\alpha}^{*T}+\boldsymbol{G}_{0}$, where $\boldsymbol{M}_{0}\boldsymbol{=}E\boldsymbol{(M|}F_{x}=\boldsymbol{x}_{1}\boldsymbol{)}$ and $\boldsymbol{G}_{0}=E\left( \boldsymbol{G} | F_{x}=\boldsymbol{x}_{1} \right)\boldsymbol{\Gamma}^{T}$ are constant vectors independent of $\boldsymbol{x}_{1}$ following (2). Thus $\boldsymbol{\alpha}^{\boldsymbol{*}}$ can be interpreted causally, as it describes how the mean of $\boldsymbol{Y}$ responds to manipulation of $\boldsymbol{x}$. Next, we show how to estimate $\boldsymbol{\alpha}^{\boldsymbol{*}}$. Again by (3), the formula (5) is also $E\left( \boldsymbol{Y}^{T} | \boldsymbol{x},\boldsymbol{G},\boldsymbol{U},F_{x}=\emptyset\right)$. Then,

$$E\left( \boldsymbol{Y}^{T} | \boldsymbol{G},F_{x}=\emptyset\right)=E\left( \boldsymbol{M} | \boldsymbol{G},F_{x}=\boldsymbol{x}_{1} \right)\boldsymbol{+}E\left( \boldsymbol{x} | \boldsymbol{G},F_{x}=\emptyset\right)\boldsymbol{\alpha}^{*T}+\boldsymbol{G}\boldsymbol{\Gamma}^{T}$$

By (4), the first term on the right side is a constant vector including $k$ constants, thus $E\left( \boldsymbol{Y}^{T} | \boldsymbol{G},F_{x}=\emptyset\right)=constant vector\boldsymbol{+}E\left( \boldsymbol{x} | \boldsymbol{G},F_{x}=\emptyset\right)\boldsymbol{\alpha}^{*T}+\boldsymbol{G}\boldsymbol{\Gamma}^{T}$ (6). Equation (6) relates two functions of $\boldsymbol{G}$, each of which can be identified from observational data. Consequently, we can estimate the causal parameter $\boldsymbol{\alpha}^{\boldsymbol{*}}$.

## 8. Compared methods

We compared the performance of METEOR with other five existing methods: (i) IVW-R^11^, which obtains the causal effect estimate through weighting and combining the effect estimates from individual instrumental SNPs. It relies on random effects to account for horizontal pleiotropy and the heterogeneity of effect estimates across different instrumental SNPs. (ii) MR-APSS^12^, which accounts for pleiotropy and sample structure simultaneously and relies on a foreground-background model to decompose the observed SNP effect sizes, where the background model is used to account for confounding factors hidden in GWAS summary statistics, including correlated pleiotropy and sample structure, and the foreground model is used to perform causal inference while accounting for uncorrelated pleiotropy. (iii) MRAID^13^, which is a two sample MR method that can automatically select suitable instruments from a candidate set of correlated SNPs and control for both correlated and uncorrelated horizontal pleiotropy. It relies on a likelihood-based framework for causal inference. (iv) MR^214^, which is a multiple exposures and multiple outcomes MR method that uses a sparse Bayesian Gaussian copula regression framework to detect causal effects while estimating the residual correlation between outcomes. It is formulated on summary-level data of genetic association and assumes the genetic associations with the exposure and that with the outcome are taken from two distinct cohorts with non-overlapping samples. (v) MrDAG^15^, which is a Bayesian causal graphical model for summary-level MR analysis to detect the dependency within the exposures, the outcomes, and between them, to improve causal effects estimation. MrDAG totally combines three causal inference strategies, including using genetic variants as instrumental variables to account for unobserved confounders, performing structure learning to detect and orientate the direction of the dependencies within the exposures and the outcomes, and finally employing interventional calculus to derive the principled causal effect estimates.

## 9. Parameter settings for simulations with varying one parameter at a time

We set the sample sizes of exposure and outcomes to be 20,000, 50,000 or 100,000, the number of outcomes to be 2 or 4, $PVE_{\tilde{G}_{1}}$ to be 10% or 15%, $\pi_{1k}$ to be 0.1, 0.2 0.3 or 0.4 and $PVE_{hk}$ to be 5% or 10%. In addition, for $PVE_{\alpha k}$, we set it to be 0.05%, 0.075% or 0.1% when the sample size was 50,000, and set it to 0.1%, 0.15%, 0.2% or 0.25% when the sample size was 20,000. For the correlations among outcomes, we first set the number of outcomes to two with sample sizes $n_{1}=n_{2k}=20,000$ and $\tilde{\rho}_{y_{1},y_{2}}$ to 0, 0.5 or 0.9 for $k\in\left[ 1,2 \right]$. We also set the number of outcomes to four with sample sizes $n_{1}=n_{2k}=50,000$ and the correlation between each two outcomes to be $\tilde{\rho}_{u,v}=q^{\left| k_{1}-k_{2} \right|}$ for $k,k_{1}, k_{2}\in\left[ 1,\cdots,4 \right]$ and $u,v\in\left[ y_{1},\cdots,y_{4} \right]$, where $q$ was randomly drawn from 0.5, 0.7, and 0.9, leading to the correlation ranged from 0.125 to 0.9. In addition, we set various priors for $\pi_{\beta}$ (Beta (0.5, 49.5), Beta (0.5, 4.5) and Beta (0.5, 2.0)), $\pi_{1}$ (Beta (0.5, 2.0), Beta (0.5, 1.5) and Beta (0.5, 1.0)) and $\pi_{0}$ (Beta (0.05, 49.95), Beta (0.05, 9.95) and Beta (0.05, 4.95)). Furthermore, we designed a setting without horizontal pleiotropy, with $PVE_{\tilde{G}_{1}}=10\%$, $M=100$, $K=2$, $\pi_{1k}=0$, $PVE_{hk}=0$, $n_{1}=n_{2k}=20,000 (k=1,2)$, $\tilde{\rho}_{y_{1},y_{2}}=0.5$, $\tilde{\rho}_{x,y_{1}}=\tilde{\rho}_{x,y_{2}}=0$, $\boldsymbol{PV}\boldsymbol{E}_{\alpha}=\left( 0,0 \right)^{T}$ in null simulations, and $\boldsymbol{PV}\boldsymbol{E}_{\alpha}=\left( 0.15\%,0 \right)^{T}$ or $\boldsymbol{PV}\boldsymbol{E}_{\alpha}=\left( 0.15\%,0.15\% \right)^{T}$ in alternative simulations. For the above settings, we assumed that the non-zero causal effects of the exposure on different outcomes had the same sign. We also considered an opposite causal effects setting with, $\boldsymbol{\alpha}=\left( \alpha_{1},\alpha_{2} \right)^{T}=\left( -\sqrt{\frac{PVE_{\alpha_{1}}}{PVE_{\tilde{G}_{1}}}},\sqrt{\frac{PVE_{\alpha_{2}}}{PVE_{\tilde{G}_{1}}}} \right)^{T}$.

## 10. Parameter settings for simulations with varying combinations of sample overlap proportions and correlations

(1) Absence of sample overlap and correlation: one exposure and four outcomes were included, with each drawn from a distinct dataset. (2) Presence of sample overlap but absence of correlation: a. the exposure was from one dataset while the two outcomes were from another dataset, with $\tilde{\rho}_{u,v}=0$ for $u,v\in[x,y_{1},y_{2}]$; b. the exposure and both outcomes were drawn from the same dataset but with $\tilde{\rho}_{u,v}=0$ for $u,v\in[x,y_{1},y_{2}]$. (3) Presence of both sample overlap and correlations: a. the exposure was from one dataset and the two outcomes were from another dataset, with $\tilde{\rho}_{y_{1},y_{2}}=0.5$ and $\tilde{\rho}_{x,y_{1}}=\tilde{\rho}_{x,y_{2}}=0$; b. the exposure and both outcomes were from the same dataset, with $\tilde{\rho}_{u,v}=$ 0.5, 0.7 or 0.9 for $u,v\in[x,y_{1},y_{2}]$; c. the exposure and one outcome were from the same dataset with $\tilde{\rho}_{x,y_{k_{1}}}=0.5$, while the other outcome was from an independent dataset with $\tilde{\rho}_{x,y_{k_{2}}}=\tilde{\rho}_{y_{k_{1}},y_{k_{2}}}=0$, where $k_{1}\neq k_{2}$ and $k_{1},k_{2}\in[1,2]$.

## 11. Simulation settings for assessing the robustness of METEOR under violations of normal distribution and linear genetic effects

First, to assess the robustness against the non-normality, we relaxed the normality assumption for the exposure residuals by considering a t distribution with different degrees of freedom ($df = 30$, $10$, and $5$) to represent varying levels of heavy-tailed behavior, with the small degrees of freedom representing the large deviation of normal distribution. Specifically, each element of the exposure residuals was simulated as

$e_{x_{i}}\sim\frac{t\left( df \right)}{sqrt(\frac{df}{df-2})}\times\sqrt{1-PVE_{\tilde{G}_{1}}}$,

where $PVE_{\tilde{G}_{1}}=10\%$ denotes the proportion of exposure variance explained by genetic effects. Note that we adopted this design to ensure that the exposure variance explained by genetic effects and residuals to be consistent with that in the baseline setting. In contrast, the residuals for the two outcomes were still simulated from a bivariate normal distribution as in the baseline setting, and all other parameters were keep unchanged.

Second, to investigate the impact of nonlinear genetic effects, we introduced a quadratic term in the relationship between genetic variants and the exposure. Specifically, the data were simulated according to

$\boldsymbol{x}={\tilde{\boldsymbol{G}}}_{1}\boldsymbol{\beta}_{1}\boldsymbol{+}({\tilde{\boldsymbol{G}}}_{1}\odot{\tilde{\boldsymbol{G}}}_{1})\boldsymbol{\beta}_{2}+\boldsymbol{e}_{x}$,

$\boldsymbol{y}_{k}={\tilde{\boldsymbol{G}}}_{2k}\boldsymbol{\beta}_{1}\alpha_{k}+{\tilde{\boldsymbol{G}}}_{hk}\boldsymbol{\eta}_{k}+\boldsymbol{e}_{y_{k}},k=1,\cdots,K$.

where $\odot$ denotes the Hadamard (element-wise) product. The effect sizes were sampled as $\beta_{1j}\sim N\left( 0,\frac{PVE_{\tilde{G}_{1}}}{2p} \right)$ and $\beta_{2j}\sim N\left( 0,\frac{PVE_{\tilde{G}_{1}}}{6p} \right)$, and the exposure residuals were generated as $e_{x_{i}}\sim N(0,1-PVE_{\tilde{G}_{1}})$. This setup ensured that each of the linear and quadratic components explained half of $PVE_{\tilde{G}_{1}}$. Notably, only the linear component ${\tilde{\boldsymbol{G}}}_{2k}\boldsymbol{\beta}_{1}$ was used to generate the outcome, consistent with the baseline setting, while all other parameters remained unchanged.

## 12. Simulation settings for assessing the contribution of key components in METEOR

First, to demonstrate the advantage of METEOR to self-adaptively control horizontal pleiotropy, we reran METEOR with the pleiotropy parameter $\boldsymbol{\eta}=\boldsymbol{0}$ and compared it with METEOR in the baseline setting. Second, to evaluate the capacity of METEOR to self-adaptively determine instruments from correlated SNPs, we reran METEOR by pre-specifying a set of independent SNPs as IVs through LD clumping, and compared it with METEOR in the baseline setting. Third, to assess the ability of METEOR in accounting for correlations among the exposure and multiple outcomes, we reran METEOR by forcing $\boldsymbol{\Omega}$ to be the identity matrix and also reran METEOR with one outcome at a time, and compared them with METEOR in terms of power under a four-outcome setting without sample overlap between exposure and each outcome, while the four outcomes were from the same dataset. Additionally, we compared METEOR by forcing $\boldsymbol{\Omega}$ to be an identity matrix with METEOR in terms of type I error control under

## 13. Implementation details for type I error control and power evaluation

For the four methods that produced $p$-values including IVW-R, MRAID MR-APSS, and METEOR, we conducted 500 replicates per scenario to assess type I error control for global and single tests based on quantile-quantile (QQ) plots, and conducted 100 replicates per scenario (at least one non-zero causal effect) to evaluate the power for global and single tests based on Bonferroni correction, with the threshold of $p$-value was $\frac{0.05}{100}=5\times{10}^{-4}$. Specifically, in the two-outcome scenario where the exposure had no effect on one outcome but a non-zero effect on the other, we assessed the false discoveries for the null outcome and true discoveries for the other. For the global test, we followed previous studies^16, 17^ to obtain the global $p$-values of the univariate MR methods, where the global $p$-values were defined as $min(Kp_{1}, Kp_{2}, \ldots, Kp_{K}, 1)$, with $p_{1},\cdots,p_{K}$ being the $p$-values for the effects of the exposure on the multiple outcomes, respectively. In MR^2^ and MrDAG, we declared a significant causal association of an exposure-outcome pair when the PIP exceeds 0.5. We did not include the global tests for MR^2^ and MrDAG for comparison due to the difficulty in obtaining their global PIPs. For the single tests, we evaluated the type I error control of MR^2^ and MrDAG using the boxplots of PIPs in null simulations based on 500 null replicates, evaluated the power analogously to methods that produce $p$-values, and also evaluated their performance using the receiver operating characteristic (ROC) curves following their recommendation, where the true positive rate (TPR) was plotted against the false positive rate (FPR)^14^. The ROC curves were generated using data from $100$ null replicates, and $100\times K$ alternative replicates from the setting where the exposure causally affected one outcome to the setting where the exposure simultaneously affected $K$ outcomes. For example, under the baseline setting with $K=2$, we conducted 100 simulation replicates for $\boldsymbol{PV}\boldsymbol{E}_{\alpha}=\left( 0,0 \right)^{T}$, $\boldsymbol{PV}\boldsymbol{E}_{\alpha}=\left( 0.075\%,0 \right)^{T}$, and $\boldsymbol{PV}\boldsymbol{E}_{\alpha}=\left( 0.075\%,0.075\% \right)^{T}$, respectively, leading to 100 null replicates and 200 alternative replicates. We additionally generated the ROC curves for METEOR and compared its performance with MR^2^ and MrDAG. Besides the power comparison across different methods using their own criteria​​, we also evaluated the power based on an unified FDR of 0.05 for global and single tests in the baseline setting. Specifically, we conducted 100 alternative simulations along with 400 null simulations, repeating this analysis five times to calculate the average power across these replicates.

## 14. Details of real data processing

Both positive and negative control analyses were evaluated in two sample MR and one sample MR settings (100% sample overlap between exposure and outcome).To create the datasets with potential sample overlap, we utilized individual data from the UK Biobank. Specifically, we randomly divided all individuals into two equally sizes, non-overlapping datasets. One dataset was randomly assigned as the exposure and the other as the outcome in two sample MR setting. While the exposure dataset from the two sample MR setting was used as both the exposure and outcome datasets in one sample MR setting. The UK Biobank consists of 487,298 individuals and 92,693,895 imputed SNPs^18^. We used the same sample quality control procedure in Neale lab to retain 337,129 individuals of European ancestry. Furthermore, we filtered out SNPs with a Hardy Weinberg equilibrium (HWE) $p<{10}^{-7}$, a genotype call rate $<95\%$, or a minor allele frequency (MAF) $<0.001$, resulting in 13,876,958 SNPs for analysis. For each trait in turn, we removed the effects of sex and top 10 genotype principal components (PCs) to obtain the residuals, standardized the residuals to have a mean of zero and a standard deviation of one, and used these scaled residuals for subsequent analysis. We obtained summary statistics for each trait through linear regression implemented in PLINK.

For the shared exposure detection analysis to identify the metabolic risk factors for the multimorbidity across CVDs and MDs underlying the brain-heart axis, we selected CVD outcomes from the “diseases of the circulatory system” category including 108 phenotypes and MD outcomes from the “mental and behavioral disorders” category including 95 phenotypes, using summary data from FinnGen^19^ (<https://www.finngen.fi/en>). We only involved the outcomes with the number of cases exceeding 30,000, retaining 15 and 4 phenotypes for CVDs and MDs, respectively. Finally, we excluded 7 poorly defined phenotypes as well as 5 unrelated with CVDs or MDs, resulting in a final set of 5 CVD and 2 MD phenotypes, with an average case number of 49,996 (details in Supplementary Tables 3-4). We treated the multimorbidity across the remaining 5 CVDs and 2 MDs as outcomes.

We used the same $p$-value threshold ($5\times{10}^{-8}$) and $r^{2}$ as that in simulations to select instrumental SNPs. For MRAID and METEOR, if the number of instrumental SNPs exceeded 10,000, we further performed LD clumping to select SNPs with an $r^{2}$ of 0.5. We also used the same significance criteria as that in simulations.

## 15. Results of exposure detection analyses

Most signals from other methods were also detected by METEOR with consistent directions and generally smaller $p$-values. In particular, 24 out of the 25 significant causal pairs identified by IVW-R were also detected by METEOR with consistent effect directions, where the $p$-values of 23 causal pairs from METEOR were smaller than those from IVW-R (Figure 4F and Figure 5A). All 24 significant causal pairs identified by MR-APSS were also identified by METEOR with consistent effect directions and smaller $p$-values (Figure 4F and Figure 5A). Similarly, 16 out of the 26 significant causal pairs identified by MRAID were also identified by METEOR with consistent effect directions, among which the $p$-values of 12 causal pairs from METEOR were smaller than those from MRAID (Figure 4F and Figure 5A). Additionally, 9 out of the 20 significant causal pairs identified by MR^2^ were also detected by METEOR under the Bonferroni correction with consistent causal effect direction (Figure 5A-C). Among the 11 causal pairs identified by MR^2^ rather than by METEOR, some seemed to be counter-intuitive and inconsistent with previous literature. For example, MR^2^ identified negative causal association of LDL on HF ($\alpha=-0.075$, PIP $=0.833$), while high levels of LDL was well-documented to promote atherosclerosis and myocardial ischemia and, in the long term, may result in structural and functional cardiac damage, ultimately increasing the risk of HF^20^. Moreover, 6 significant causal pairs identified by MrDAG were also found by METEOR with Bonferroni correction, and all exhibited consistent effect directions (Figure 5A, D-E).

METEOR identified a total of six shared metabolic exposures for the multimorbidity, consistent with IVW-R, MRAID and MR-APSS. In single tests, METEOR identified more corresponding outcomes across these exposures than IVW-R (28 vs. 25), MRAID (28 vs. 27), MR-APSS (28 vs. 24), MR^2^ (28 vs. 20) and MrDAG (28 vs. 6). For example, DBP was identified by METEOR as a shared exposure for five CVDs and two MDs, compared with five CVDs by IVW-R, three by MRAID, and two each by MR^2^ and MrDAG.

METEOR identified BMI as a shared exposure across all five CVDs. Genetically predicted higher BMI is associated with increased left ventricular hypertrophy, elevated levels of TG, glucose, insulin, and interleukin 6 (an inflammatory marker)^21^ as well as arterial hypertension, SBP, and DBP^22^, all of which may contribute to its associations with CVDs. In addition, METEOR identified blood pressure as the shared exposure with the largest number of associated outcomes across CVDs and MDs along the brain-heart axis. This finding suggests that blood pressure dysregulation is not only a well-established etiological factor for CVDs, but may also contribute to the development of MDs, potentially serving as a physiological link between cardiovascular and psychiatric conditions. Biologically, blood pressure is tightly regulated by the autonomic nervous system. Heightened sympathetic activity contributes to the development of hypertension, which in turn plays a central role in cardiovascular pathophysiology through vascular remodeling and increased cardiac workload^23, 24^. Meanwhile, autonomic dysfunction, characterized by increased sympathetic and reduced parasympathetic tone, has also been implicated in the pathogenesis of mental disorders such as anxiety and depression^25^. These shared neurophysiological mechanisms suggest that dysregulated blood pressure, as a downstream marker of autonomic imbalance, may serve as a common etiological pathway linking cardiovascular and psychiatric conditions. Furthermore, hypertension is often accompanied by chronic low-grade inflammation, endothelial dysfunction, and dysregulation of the hypothalamic-pituitary-adrenal (HPA) axis^26, 27, 28^, which are known factors that accelerate atherosclerosis, elevate the risk of CVDs, and are frequently observed in individuals with mood and anxiety disorders^29, 30, 31^. Our results underscore the importance of blood pressure control in the co-prevention and co-management of CVDs and MDs within the context of multimorbidity. Similar patterns were observed in the shared exposure detection analysis underlying brain-gut axis, where BMI emerged as a shared exposure across GI diseases, and blood pressure was identified as a shared exposure across GI diseases and MDs. Indeed, elevated BMI promotes systemic low-grade inflammation and disrupts gut microbiota composition, contributing to altered intestinal barrier function and increased susceptibility to conditions such as IBD, IBS, GDU and GERD^32^. In parallel, elevated blood pressure is associated with autonomic nervous system dysregulation and HPA axis activation, which can simultaneously affect gastrointestinal function and mental health, thereby linking GI diseases with mental disorders^27, 29, 30, 31, 33, 34^. The consistency of these biologically plausible mechanisms with our results strengthens the advantage of METEOR in accurately identifying shared exposures underlying single-system or cross-system multimorbidity.

## 16. Parameter settings for simulations with correlated horizontal pleiotropy

For the $j$-th SNP, we assumed that it had a probability $\pi_{ck}$ to exhibit correlated horizontal pleiotropy on the $k$-th outcome with the effect size being $\omega\beta_{j}$, where $\beta_{j}$ denoted the effect size of the $j$-th SNP on the exposure, $\omega$ quantified the effect of the unmeasured confounder on the outcome, while the effect of the confounder on the exposure was fixed to one following previous studies^13, 35^. In these simulations, we set $\omega=\sqrt{0.05}$ and varied $\pi_{ck}$ across 0.02, 0.05 and 0.1 for both outcomes, based on the baseline simulation setting. Larger values of $\pi_{ck}$ correspond to a higher proportion of SNPs exhibting correlated horizontal pleiotropy.

# Supplementary Figures


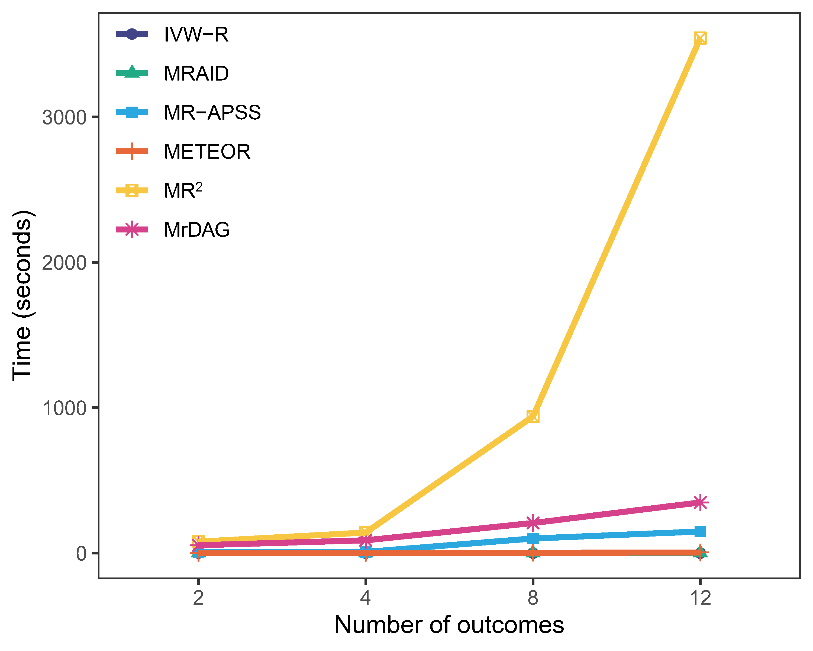


Supplementary Figure 1 Computational time of different methods. All computations were carried out on a single thread of an Intel Xeon Gold E5-2697 v3 CPU. Reported running time are averaged over 500 replicates under the baseline scenario. The number of instrumental variables is fixed at 50. The x-axis represents the number of outcomes, and the y-axis represents the running time in seconds.


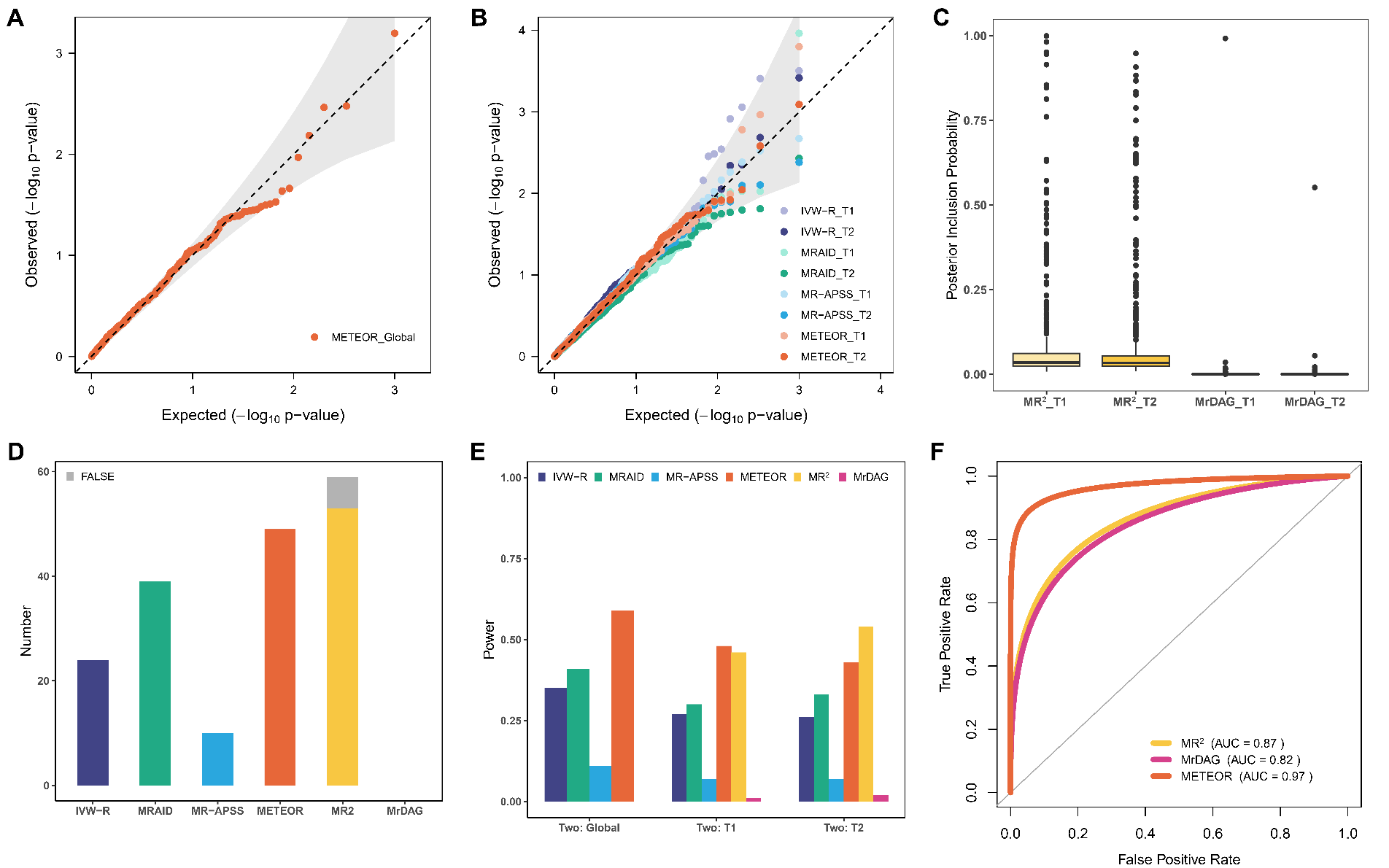


Supplementary Figure 2 Results from simulations with sample sizes of 20,000 for exposure and two outcomes. The scenario involves the following parameters: $PVE_{\tilde{G}_{1}}=10\%$, $K=100$, $\pi_{1k}=20\%$, $PVE_{hk}=5\%$ ($k=1,2$), $\tilde{\rho}_{y_{1},y_{2}}=0.5$ and $\tilde{\rho}_{x,y_{1}}=\tilde{\rho}_{x,y_{2}}=0$. (A) QQ plot from the global test of METEOR in the null simulations. (B) QQ plots from IVW-R, MRAID, MR-APSS and METEOR in testing the causal effects of exposure on the both outcomes (T1 and T2) in null simulations. (C) PIPs from MR^2^ and MrDAG for the two outcomes in the null simulations. Power performance under Bonferroni adjusted $p$-value threshold of $5\times{10}^{-4}$ for global and single tests. (D) Numbers of true discovery and false discover (grey) for all methods in the baseline setting with $\boldsymbol{PV}\boldsymbol{E}_{\alpha}=\left( 0.15\%,0 \right)^{T}$. (E) The results are plotted under $\boldsymbol{PV}\boldsymbol{E}_{\alpha}=\left( 0.15\%,0.15\% \right)^{T}$ (‘Two: Global’ for global test; ‘Two: T1’ and ‘Two: T2’ for single tests). (F) ROC curves for MR^2^, MrDAG and METEOR by plotting the TPR against FPR.


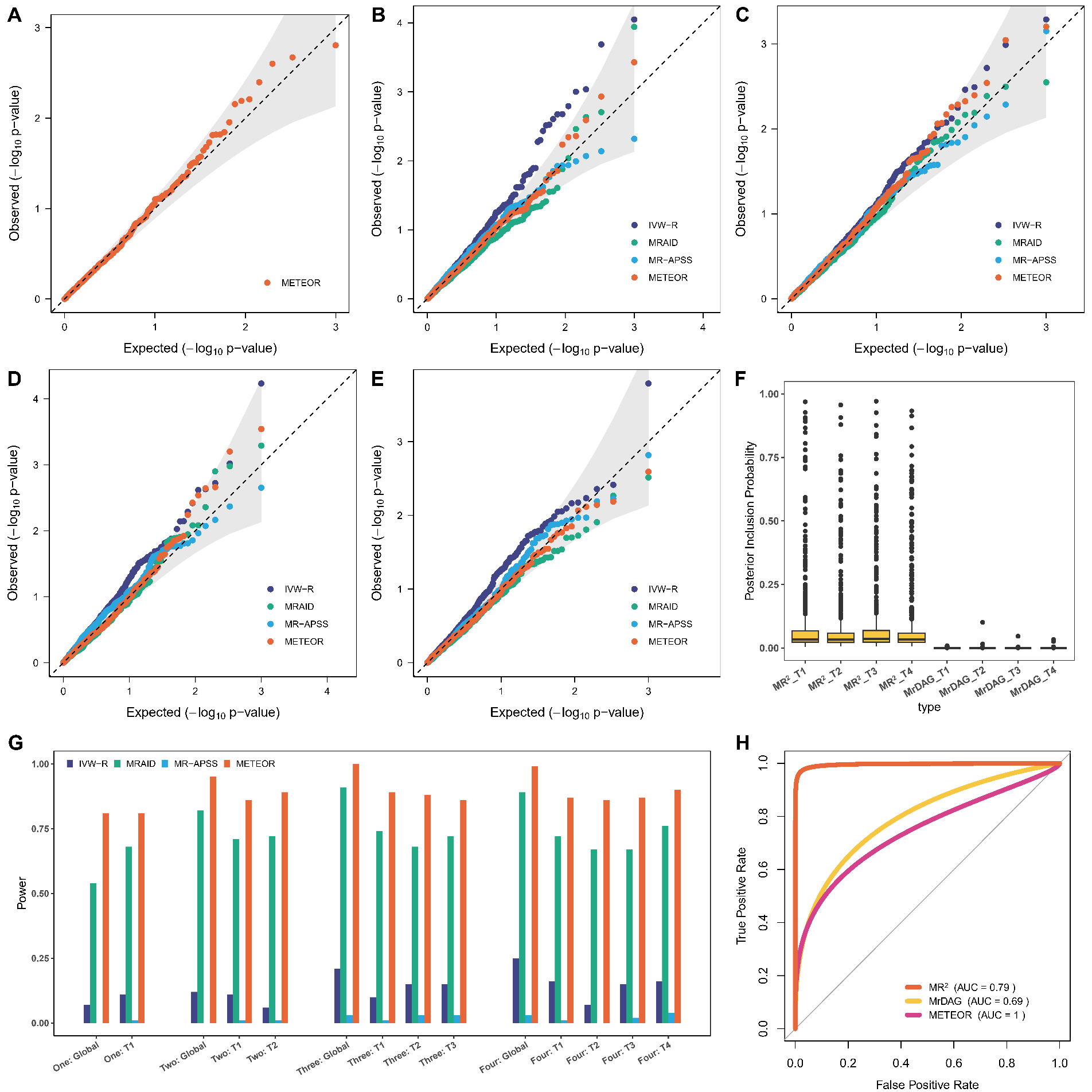


Supplementary Figure 3 Results from simulations with one exposure and four outcomes. The scenario includes the following parameters: $PVE_{\tilde{G}_{1}}=10\%$, $K=100$, $\pi_{1k}=20\%$, $PVE_{hk}=5\%$, $n_{1}=n_{2k}=50,000 (k=1,\cdots,4)$, with a correlation of 0.5 between any two outcomes and a correlation of 0 between exposure and each outcome. In alternative simulations, $PVE_{\alpha k}=0.075\%$. Type I error control is evaluated using quantile-quantile (QQ) plots of $-\log_{10} p$ values. (A) QQ plot from the global test of METEOR in null simulations. QQ plots from IVW-R, MRAID, MR-APSS and METEOR in testing the causal effect of exposure on (B) the first, (C) second, (D) third and (E) fourth outcome in null simulations. (F) Posterior inclusion probabilities (PIPs) from MR^2^ and MrDAG for the four outcomes (T1, T2, T3 and T4). (G) Power performance under Bonferroni adjusted $p$-value threshold of $5\times{10}^{-4}$ for global and single tests. The results from IVW-R, MRAID, MR-APSS and METEOR are plotted for four alternative scenarios: ranging from a setting where the exposure causally affects one trait to a setting where the exposure causally affects four traits. (H) Receiver operating characteristic (ROC) curves for MR^2^, MrDAG and METEOR by plotting the true positive rate (TPR) against the false positive rate (FPR).


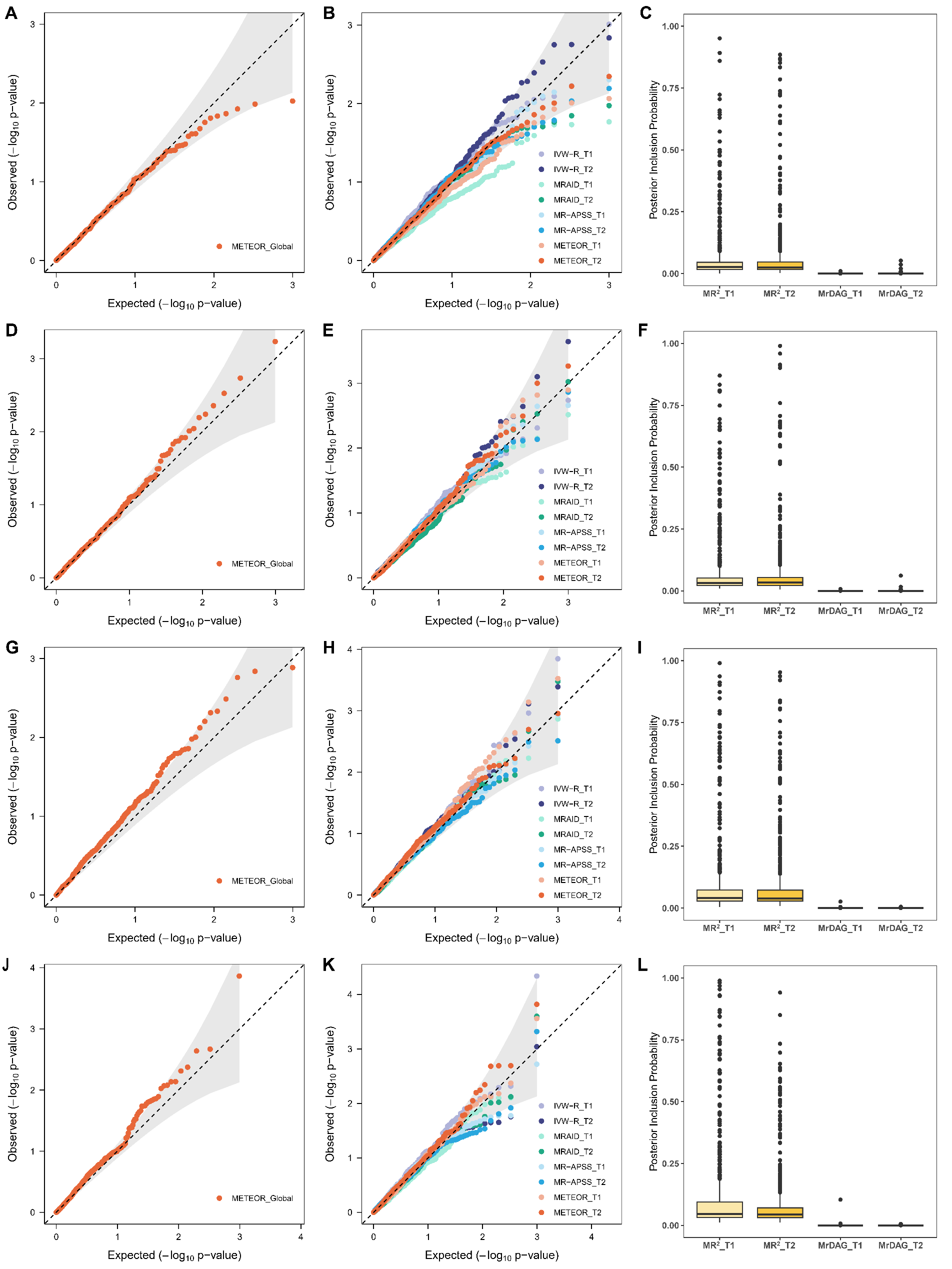


Supplementary Figure 4 Quantile-quantile (QQ) plots from simulations with various $\pi_{1k}$. The scenario involves one exposure and two outcomes, with the following parameters: $PVE_{\tilde{G}_{1}}=10\%$, $K=100$, $PVE_{hk}=5\%$, $n_{1}=n_{2k}=50,000 (k=1,2)$, $\tilde{\rho}_{y_{1},y_{2}}=0.5$ and $\tilde{\rho}_{x,y_{1}}=\tilde{\rho}_{x,y_{2}}=0$. Four values of $\pi_{1k}$ are considered:0.1, 0.2, 0.3 and 0.4, listed from top to bottom. (A, D, G, J) QQ plots from the global tests for METEOR. (B, E, H, K) QQ plots from IVW-R, MRAID, MR-APSS and METEOR in testing the causal effects of exposure on the both outcomes. (C, F, I, L) Posterior inclusion probabilities (PIPs) from MR^2^ and MrDAG for two outcomes (T1 and T2).


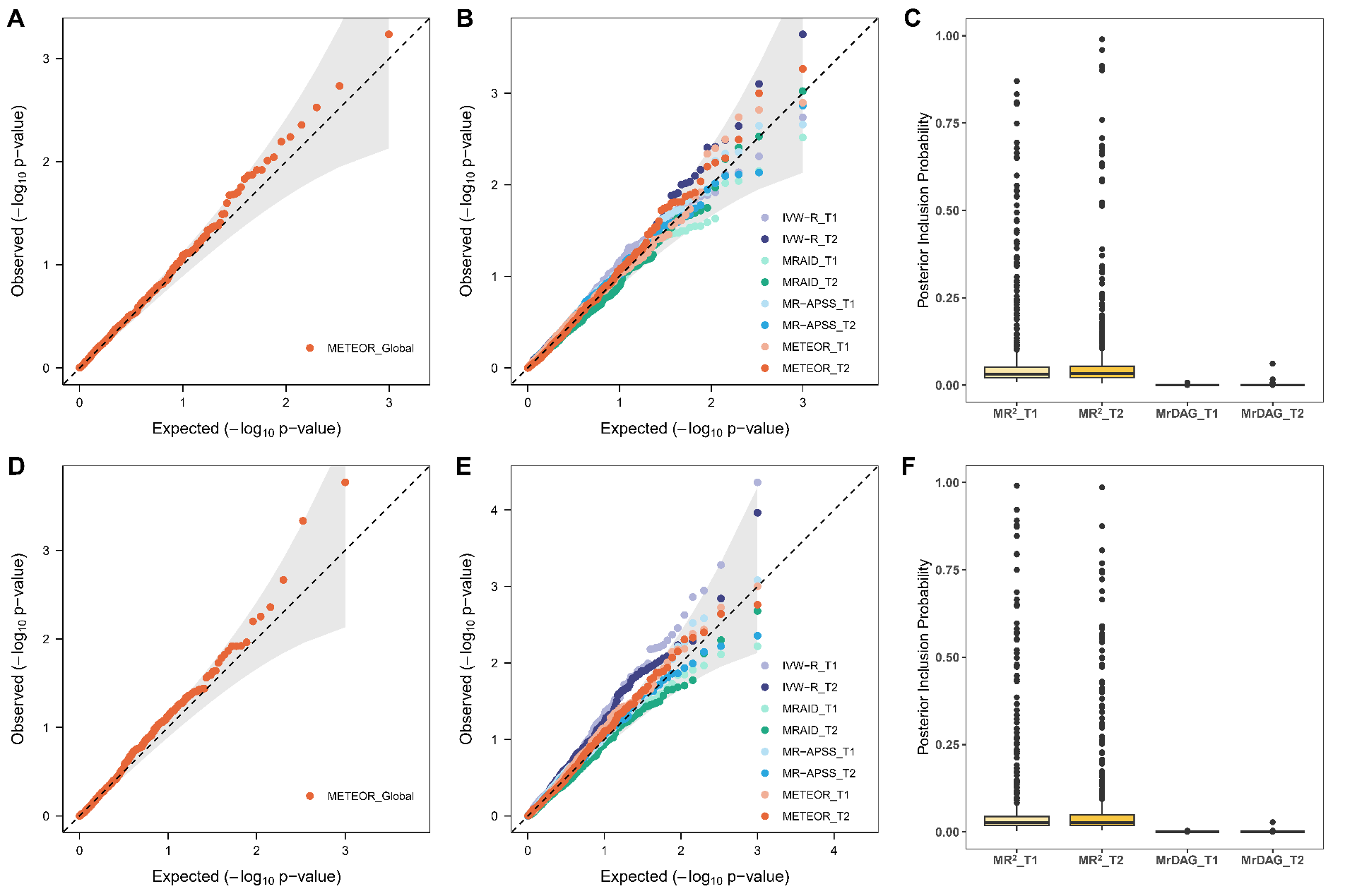


Supplementary Figure 5 Quantile-quantile (QQ) plots from simulations with various $PVE_{\tilde{G}_{1}}$. The scenario involves one exposure and two outcomes, with the following parameters: $K=100$, $\pi_{1k}=20\%$, $PVE_{hk}=5\%$,$n_{1}=n_{2k}=50,000 (k=1,2)$, $\tilde{\rho}_{y_{1},y_{2}}=0.5$ and $\tilde{\rho}_{x,y_{1}}=\tilde{\rho}_{x,y_{2}}=0$. Two values of $PVE_{\tilde{G}_{1}}$ are considered:10% and 15%, listed from top to bottom. Type I error control is evaluated using QQ plots of $-\log_{10} p$ values in null simulations. (A, D) QQ plots from the global tests of METEOR. (B, E) QQ plots from IVW-R, MRAID, MR-APSS and METEOR in testing the causal effects of exposure on both outcomes. (C, F)Posterior inclusion probabilities (PIPs) from MR^2^ and MrDAG for two outcomes (T1 and T2) in null simulations.


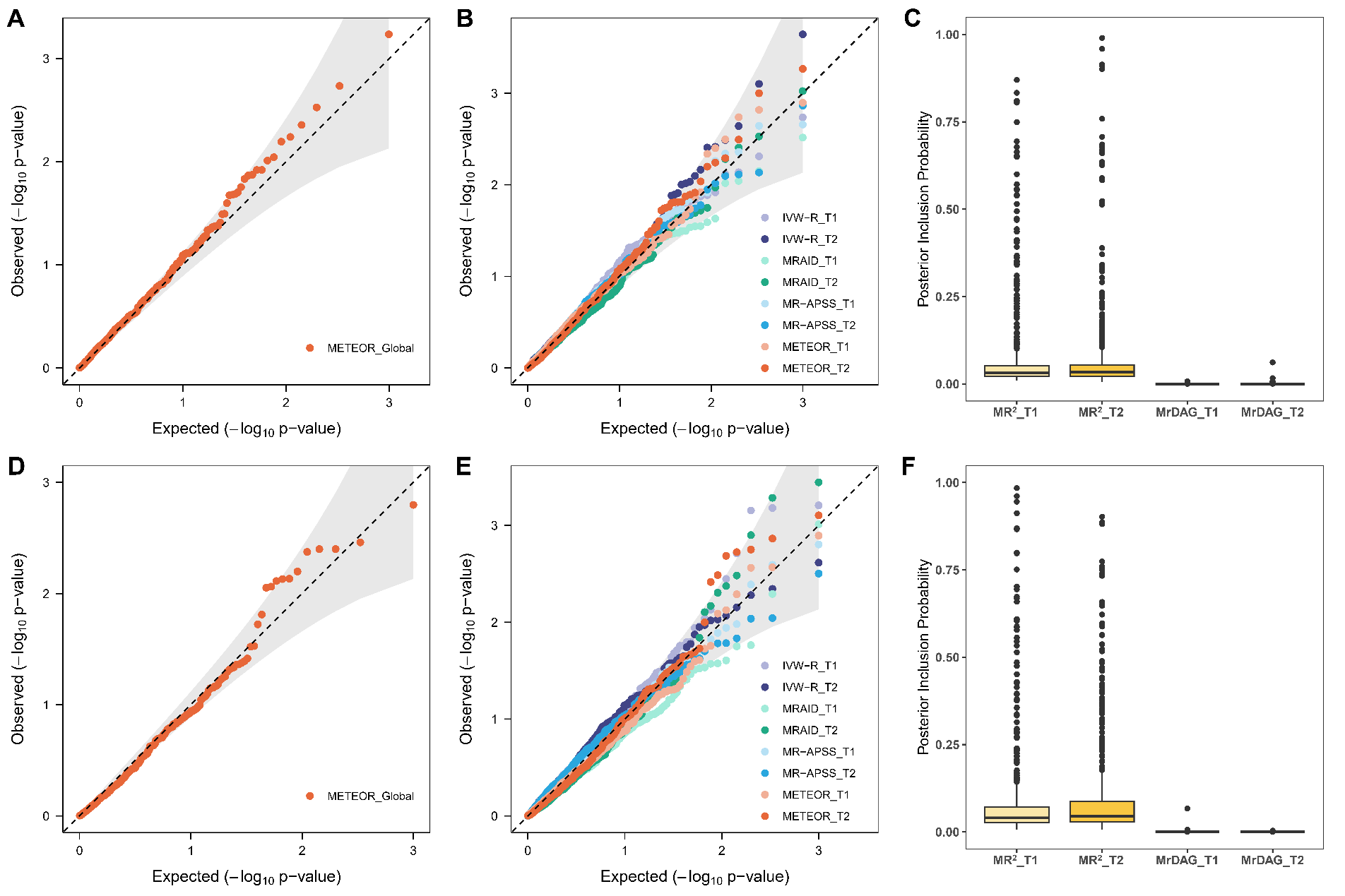


Supplementary Figure 6 Quantile-quantile (QQ) plots from simulations with various $PVE_{hk}$. The scenario involves one exposure and two outcomes, with the following parameters: $PVE_{\tilde{G}_{1}}=10\%$, $K=100$, $\pi_{1k}=20\%$, $n_{1}=n_{2k}=50,000$ ($k=1,2$), $\tilde{\rho}_{y_{1},y_{2}}=0.5$ and $\tilde{\rho}_{x,y_{1}}=\tilde{\rho}_{x,y_{2}}=0$. Type I error control is evaluated using QQ plots of $-\log_{10} p$ in null simulations. Two values of $PVE_{hk}$ are considered:5% and 10%, listed from top to bottom. (A, D) QQ plots from the global tests of METEOR. QQ plots from IVW-R, MRAID, MR-APSS and METEOR in testing the causal effects of exposure on (B, E) the both outcomes. (C, F) Posterior inclusion probabilities (PIPs) from MR^2^ and MrDAG for two outcomes (T1 and T2) in null simulations.


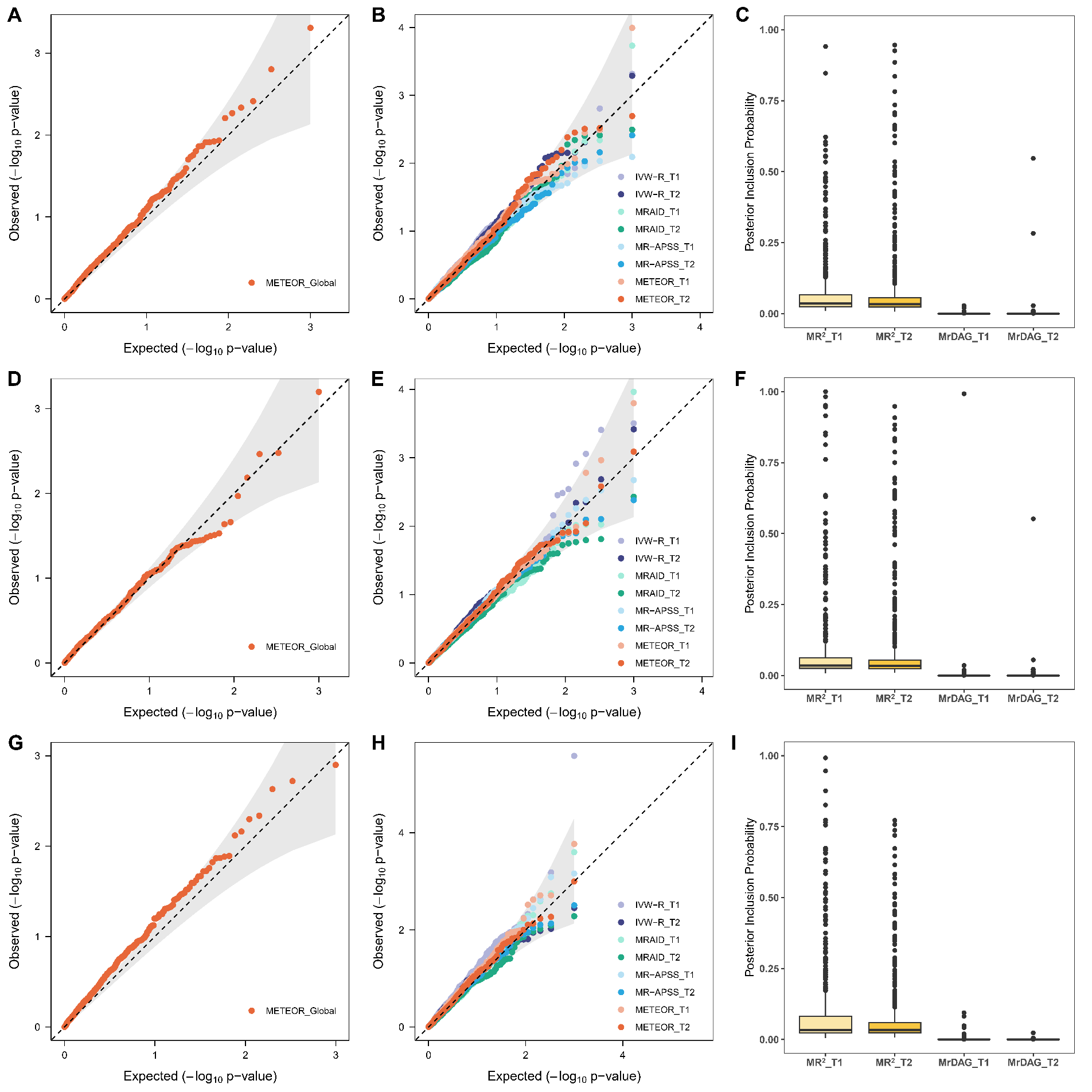


Supplementary Figure 7 Results from simulations with various correlations when sample sizes for both exposure and outcomes are 20,000. The scenario involves one exposure and two outcomes, with the following parameters: $PVE_{\tilde{G}_{1}}=10\%$, $K=100$, $\pi_{1k}=20\%$, $PVE_{hk}=5\%$ ($k=1,2$), and $\tilde{\rho}_{x,y_{1}}=\tilde{\rho}_{x,y_{2}}=0$. Three correlation values between any two outcomes are considered, including 0, 0.5 and 0.9, listed from top to bottom. Type I error control is evaluated using quantile-quantile (QQ) plots of $-\log_{10} p$ values in null simulations. (A, D, G) QQ plots from the global tests of METEOR. (B, E, H) QQ plots from IVW-R, MRAID, MR-APSS and METEOR in testing the causal effects of exposure on the both outcomes. (C, F, I) Posterior inclusion probabilities (PIPs) from MR^2^ and MrDAG for two outcomes (T1 and T2) in null simulations.


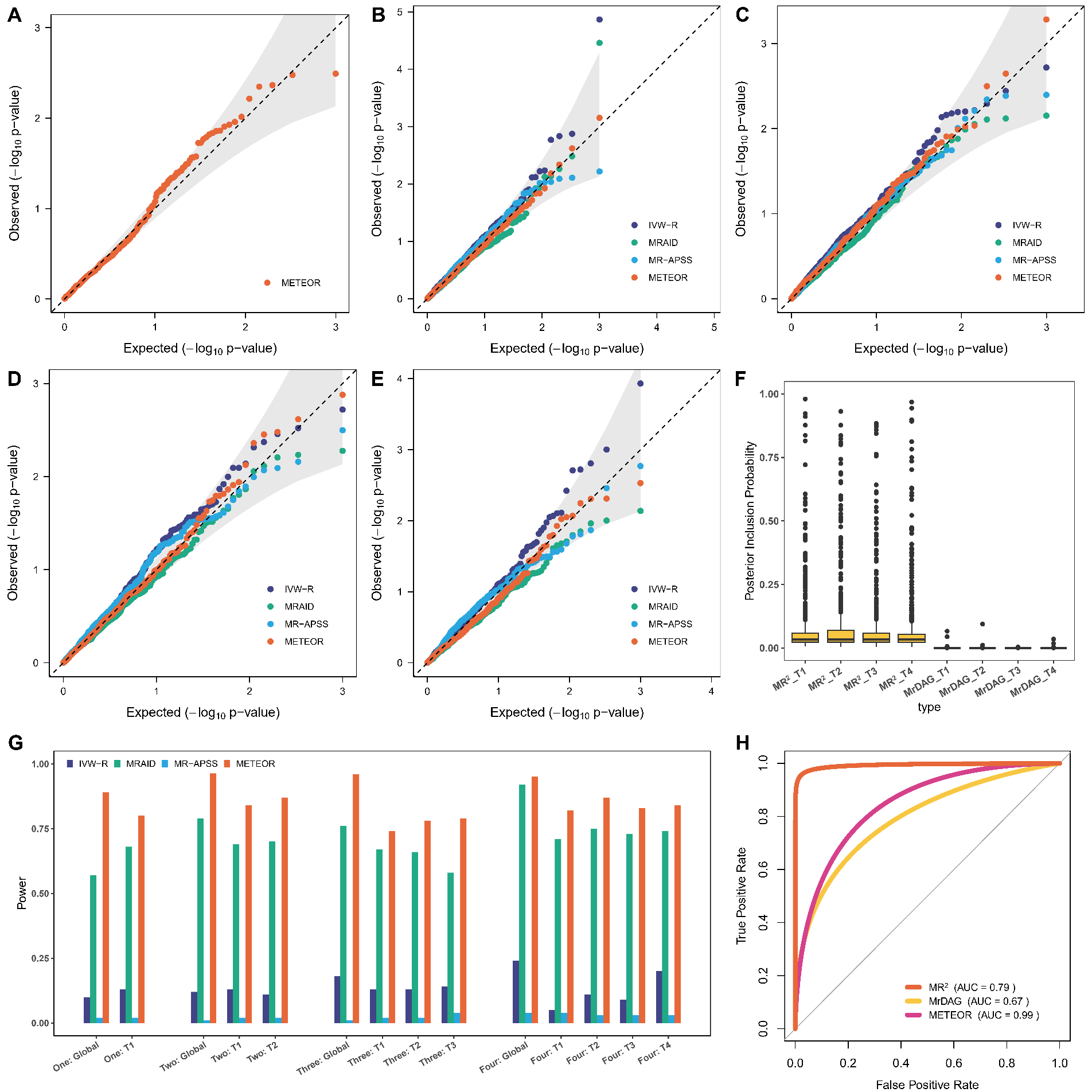


Supplementary Figure 8 Results from simulations with a complex correlation structure for four outcomes. The correlation between outcomes, $\tilde{\rho}_{u,v}=q^{\left| k_{1}-k_{2} \right|}$ for $k_{1}, k_{2}\in[1,\cdots,4]$ and $u, v\in\left[ y_{1},\cdots,y_{4} \right]$, where $q$ is randomly drawn from 0.5, 0.7 and 0.9. The outcome correlation in the exponential covariance structure ranges from 0.125 to 0.9. The scenario includes the following parameters: $PVE_{\tilde{G}_{1}}=10\%$, $K=100$, $\pi_{1k}=20\%$, $PVE_{hk}=5\%$, $n_{1}=n_{2k}=50,000 (k=1,\cdots,4)$, with a correlation of 0 between exposure and each outcome. In alternative simulations, $PVE_{\alpha k}=0.075\%$. Type I error control is evaluated using quantile-quantile (QQ) plots of $-\log_{10} p$ values in null simulations. (A) QQ plot from the global test of METEOR. QQ plots from IVW-R, MRAID, MR-APSS and METEOR in testing the causal effects of exposure on (B) the first, (C) second, (D) third and (E) fourth outcome. (F) Posterior inclusion probabilities (PIPs) from MR^2^ and MrDAG for the four outcomes (T1, T2, T3 and T4) in null simulations. (G) Power performance under Bonferroni adjusted $p$-value threshold of $5\times{10}^{-4}$ for global and single tests. The results from IVW-R, MRAID, MR-APSS and METEOR are plotted for four alternative scenarios: ranging from a setting where the exposure causally affects one trait to a setting where the exposure causally affects four traits. (H) Receiver operating characteristic (ROC) curves for METEOR and MR^2^, plotting the true positive rate (TPR) against the false positive rate (FPR).


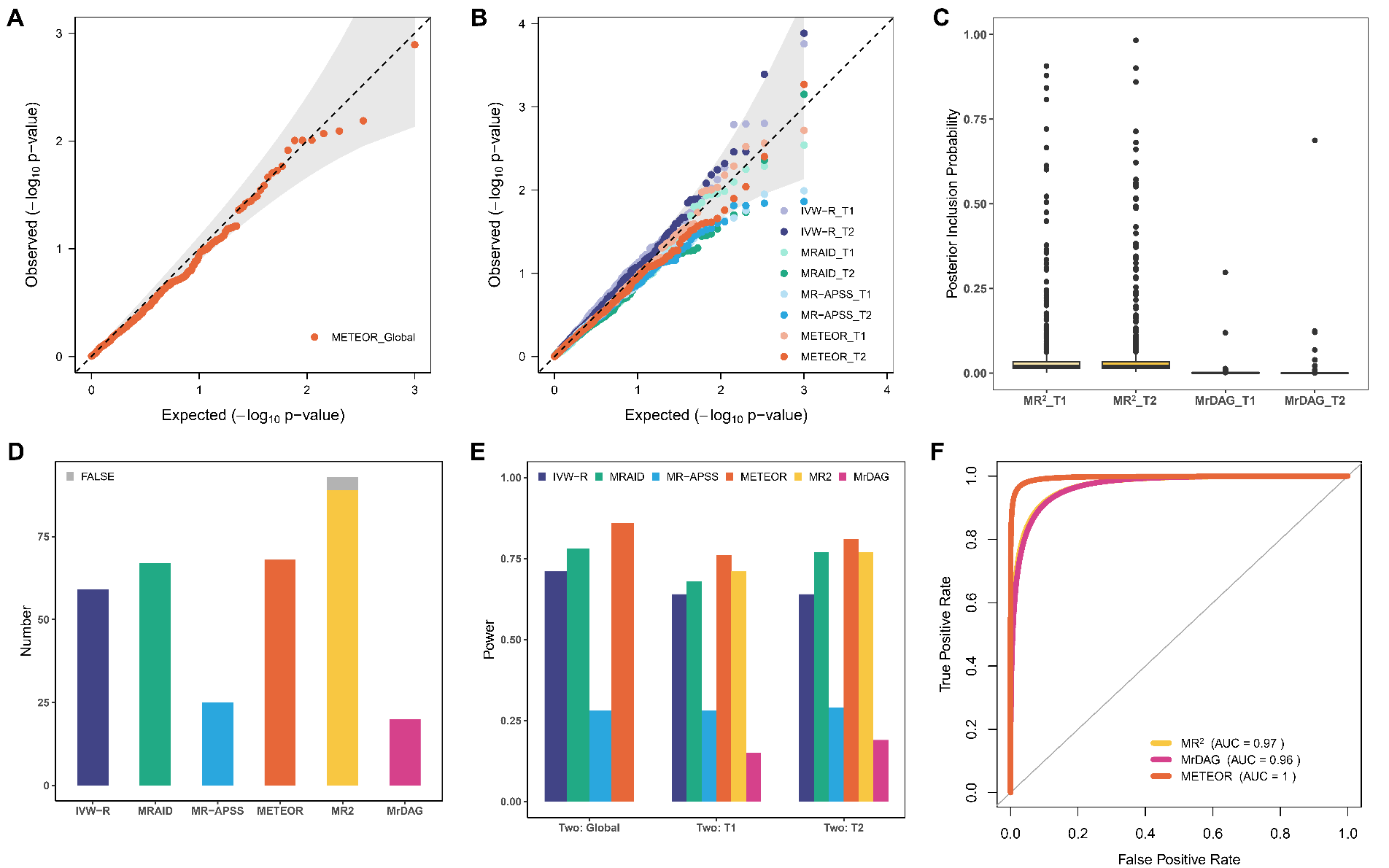


Supplementary Figure 9 Results from simulations with no horizontal pleiotropy when sample sizes for both exposure and outcomes are 20,000. The scenario involves one exposure and two outcomes, with the following parameters: $PVE_{\tilde{G}_{1}}=10\%$, $K=100$, $\pi_{1k}=0$, $PVE_{hk}=0$ ($k=1,2$), $\tilde{\rho}_{y_{1},y_{2}}=0.5$ and $\tilde{\rho}_{x,y_{1}}=\tilde{\rho}_{x,y_{2}}=0$. Type I error control, evaluated using quantile-quantile (QQ) plots of $-\log_{10} p$ values in null simulations. (A) QQ plot from global test of METEOR. (B) QQ plots from IVW-R, MRAID, MR-APSS and METEOR in testing the causal effects of exposure on the both outcomes. (C) Posterior inclusion probabilities (PIPs) from MR^2^ and MrDAG for the two outcomes (T1 and T2) in null simulations. Power performance under Bonferroni adjusted $p$-value threshold of $5\times{10}^{-4}$ for global and single tests. (D) Numbers of true discovery and false discovery (grey) for all methods in the baseline setting with $\boldsymbol{PV}\boldsymbol{E}_{\alpha}=\left( 0.15\%,0 \right)^{T}$. (E) The results are plotted for one alternative scenario: $\boldsymbol{PV}\boldsymbol{E}_{\alpha}=\left( 0.15\%,0.15\% \right)^{T}$. (F) Receiver operating characteristic (ROC) curves for METEOR and MR^2^ by plotting the true positive rate (TPR) against the false positive rate (FPR).


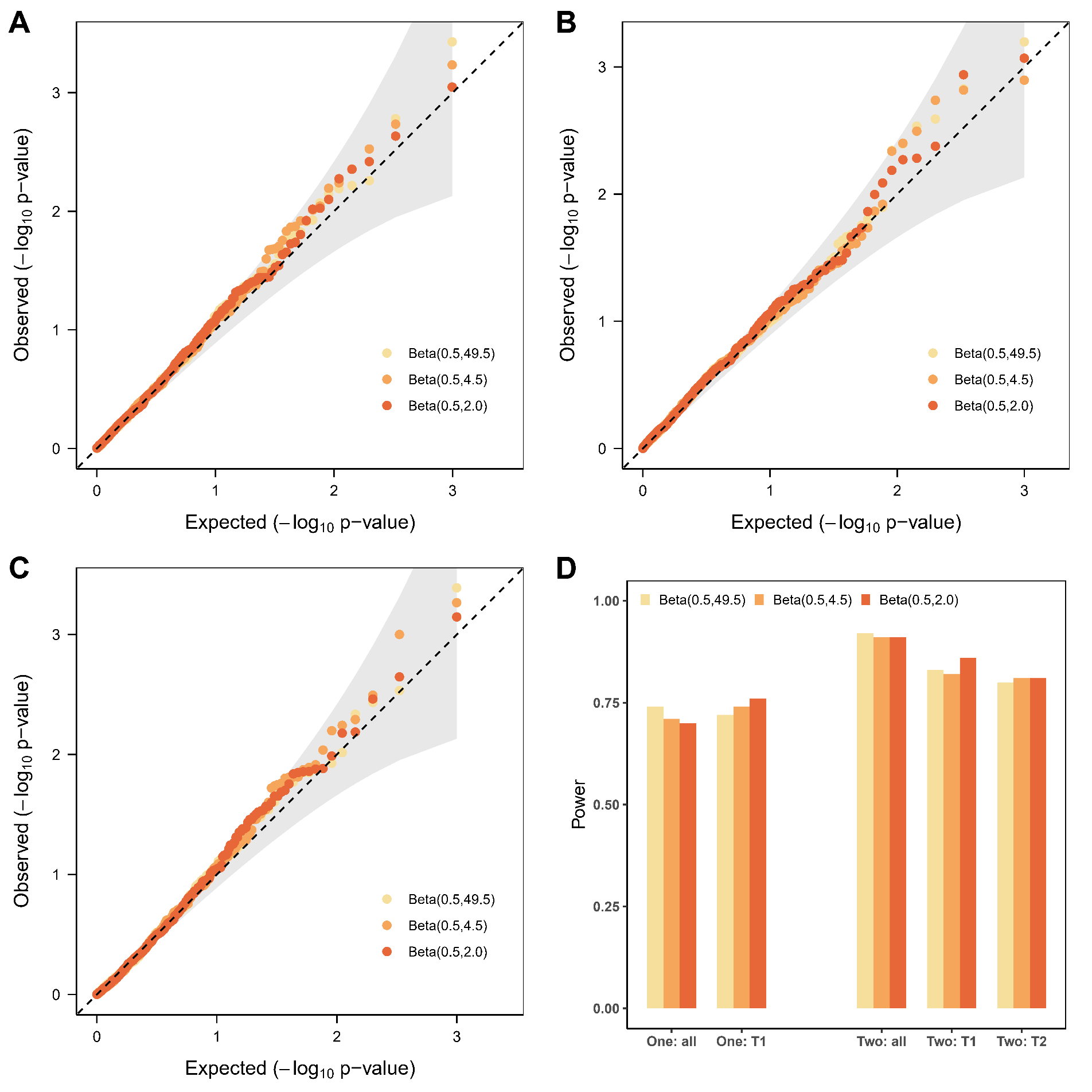


Supplementary Figure 10 Results from METEOR using different priors for $\pi_{\beta}$ in baseline scenario. Three priors are considered: Beta (0.5, 49.5), Beta (0.5, 4.5) and Beta (0.5, 2.0). The scenario involves one exposure and two outcomes, with the following parameters: $PVE_{\tilde{G}_{1}}=10\%$, $K=100$, $\pi_{1k}=20\%$, $PVE_{hk}=5\%$, $n_{1}=n_{2k}=50,000$ ($k=1,2$), $\tilde{\rho}_{y_{1},y_{2}}=0.5$ and $\tilde{\rho}_{x,y_{1}}=\tilde{\rho}_{x,y_{2}}=0$. Type I error control is evaluated using quantile-quantile (QQ) plots of $-\log_{10} p$ values in null simulations. (A) QQ plots from the global test of METEOR. QQ plots from METEOR in testing the causal effects of exposure on (B) the first and (C) the second outcomes. (D) Power performance under Bonferroni adjusted $p$-value threshold of $5\times{10}^{-4}$ for global and single tests. The results are plotted for two alternative scenarios: $\boldsymbol{PV}\boldsymbol{E}_{\alpha}=\left( 0.075\%,0 \right)^{T}$ (left) and $\boldsymbol{PV}\boldsymbol{E}_{\alpha}=\left( 0.075\%,0.075\% \right)^{T}$ (right).


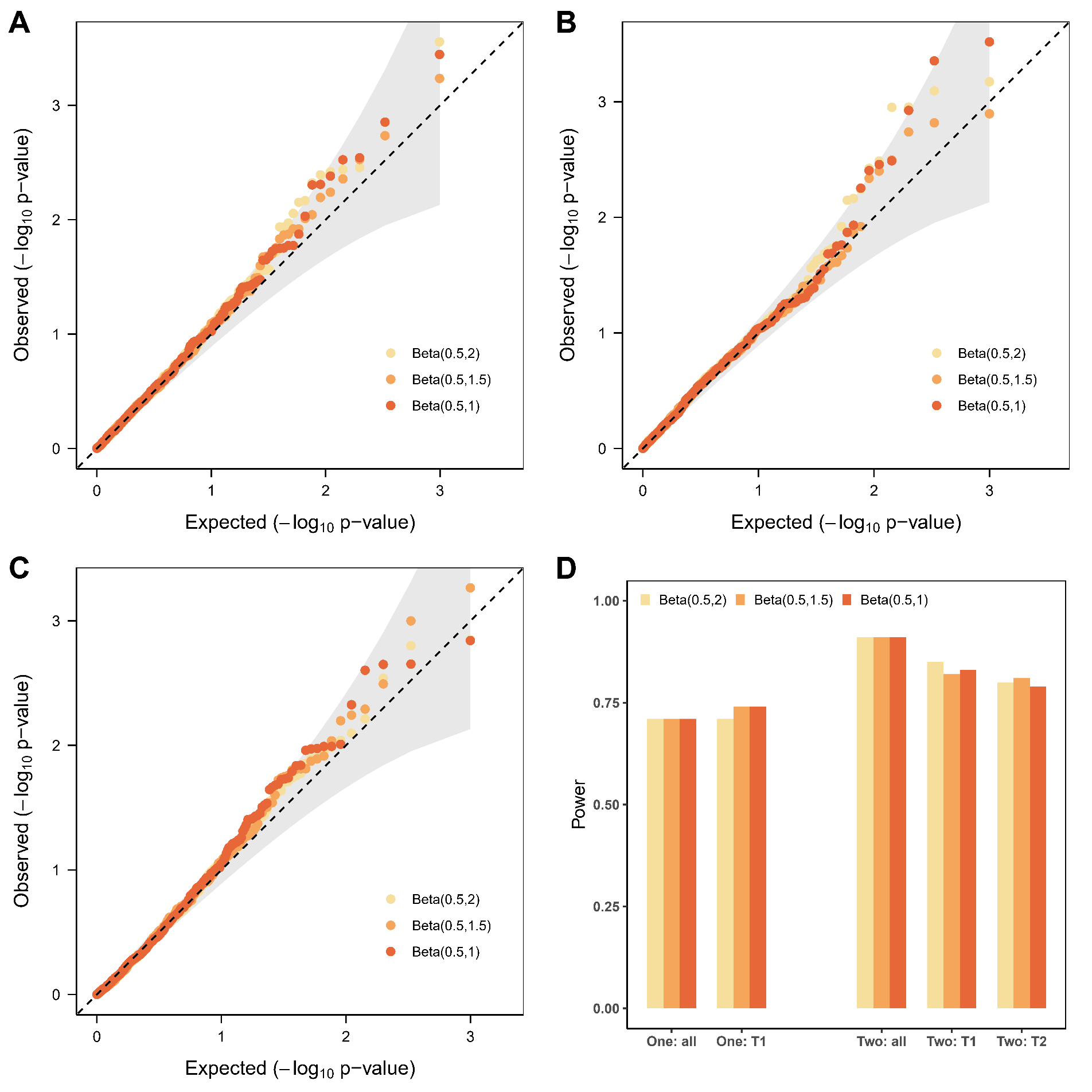


Supplementary Figure 11 Results from METEOR using different priors for $\pi_{1k}$ in baseline scenario. Three priors are considered: Beta (0.5, 2.0), Beta (0.5, 1.5) and Beta (0.5, 1.0). The scenario involves one exposure and two outcomes, with the following parameters: $PVE_{\tilde{G}_{1}}=10\%$, $K=100$, $\pi_{1k}=20\%$, $PVE_{hk}=5\%$, $n_{1}=n_{2k}=50,000$ ($k=1,2$), $\tilde{\rho}_{y_{1},y_{2}}=0.5$ and $\tilde{\rho}_{x,y_{1}}=\tilde{\rho}_{x,y_{2}}=0$. Type I error control is evaluated using quantile-quantile (QQ) plots of $-\log_{10} p$ values in null simulations. (A) QQ plots from the global test of METEOR. QQ plots from METEOR in testing the causal effects of exposure on (B) the first and (C) the second outcomes. (D) Power performance under Bonferroni adjusted $p$-value threshold of $5\times{10}^{-4}$ for global and single tests. The results are plotted for two alternative scenarios: $\boldsymbol{PV}\boldsymbol{E}_{\alpha}=\left( 0.075\%,0 \right)^{T}$ (left) and $\boldsymbol{PV}\boldsymbol{E}_{\alpha}=\left( 0.075\%,0.075\% \right)^{T}$ (right).


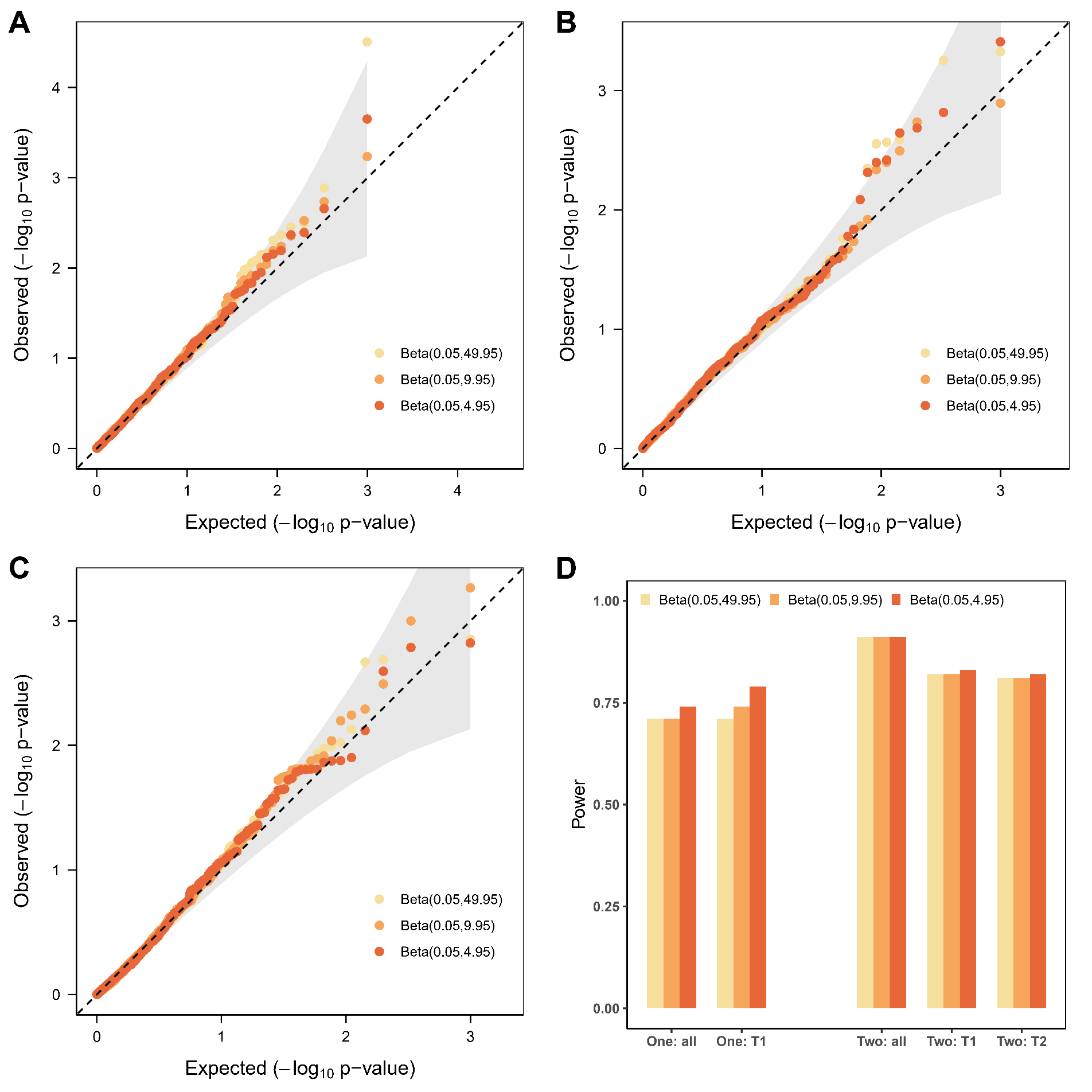


Supplementary Fig 12 Results from METEOR using different priors for $\pi_{0k}$ in baseline scenario. Three priors are considered: Beta (0.05, 49.95), Beta (0.05, 9.95) and Beta (0.05, 4.95). The scenario involves one exposure and two outcomes, with the following parameters: $PVE_{\tilde{G}_{1}}=10\%$, $K=100$, $\pi_{1k}=20\%$, $PVE_{hk}=5\%$, $n_{1}=n_{2k}=50,000$ ($k=1,2$), $\tilde{\rho}_{y_{1},y_{2}}=0.5$ and $\tilde{\rho}_{x,y_{1}}=\tilde{\rho}_{x,y_{2}}=0$. Type I error control is evaluated using quantile-quantile (QQ) plots of $-\log_{10} p$ values in null simulations. (A) QQ plots from the global test of METEOR. QQ plots from METEOR in testing the causal effects of exposure on (B) the first and (C) the second outcomes. (D) Power performance under Bonferroni adjusted $p$-value threshold of $5\times{10}^{-4}$ for global and single tests. The results are plotted for two alternative scenarios: $\boldsymbol{PV}\boldsymbol{E}_{\alpha}=\left( 0.075\%,0 \right)^{T}$ (left) and $\boldsymbol{PV}\boldsymbol{E}_{\alpha}=\left( 0.075\%,0.075\% \right)^{T}$ (right).


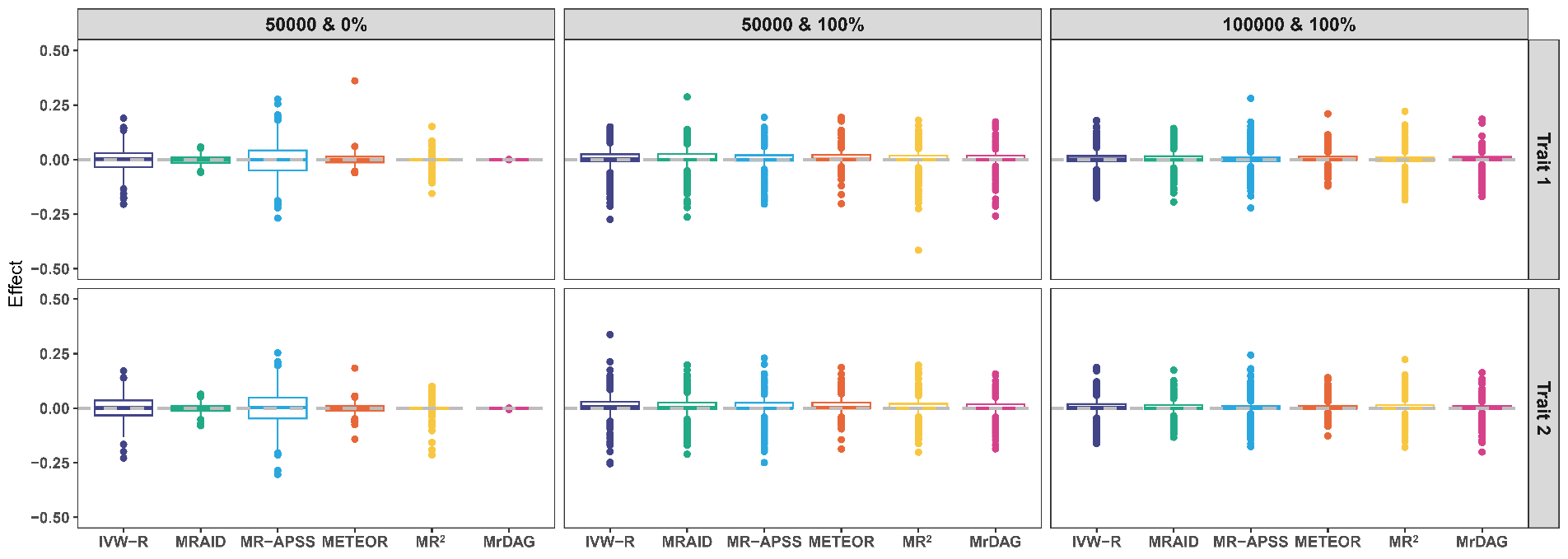


Supplementary Fig 13 Results from simulations with various proportions of sample overlap along with various sample sizes. (Left) Scenario with sample sizes for both exposure and outcomes being 50,000 and without sample overlap. (Middle) Scenario with sample sizes for both exposure and outcomes being 50,000 and with 100% sample overlap. (Right) Scenario with sample sizes for both exposure and outcomes being 100,000 and with 100% sample overlap. These scenarios involve one exposure and two outcomes, with the following parameters: $PVE_{\tilde{G}_{1}}=10\%$, $K=100$, $\pi_{1k}=20\%$, $PVE_{hk}=5\%$ ($k=1,2$), $\tilde{\rho}_{x,y_{1}}=\tilde{\rho}_{x,y_{2}}=0$, and $PVE_{\alpha}=\left( 0,0 \right)^{T}$.


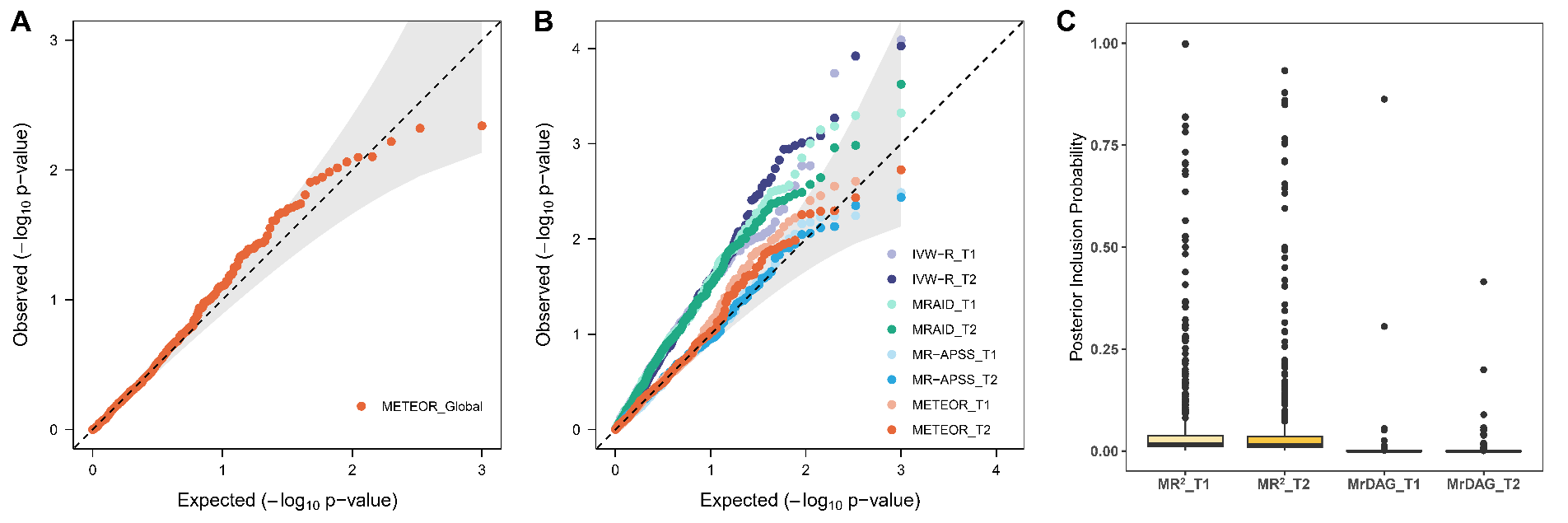


Supplementary Fig 14 Results from simulations with no horizontal pleiotropy in one sample MR setting. The scenario involves one exposure and two outcomes with sample sizes being 50,000, and with the following parameters: $PVE_{\tilde{G}_{1}}=10\%$, $K=100$, $\pi_{1k}=20\%$, $PVE_{hk}=5\%$ ($k=1,2$), $\tilde{\rho}_{x,y_{1}}=\tilde{\rho}_{x,y_{2}}=0$, and $PVE_{\alpha}=\left( 0,0 \right)^{T}$. (A) QQ plot from the global test of METEOR. (B) QQ plots from IVW-R, MRAID, MR-APSS and METEOR in testing the causal effects of exposure on the both outcomes. (C) Posterior inclusion probabilities (PIPs) from MR^2^ and MrDAG for two outcomes (T1 and T2) in null simulations.


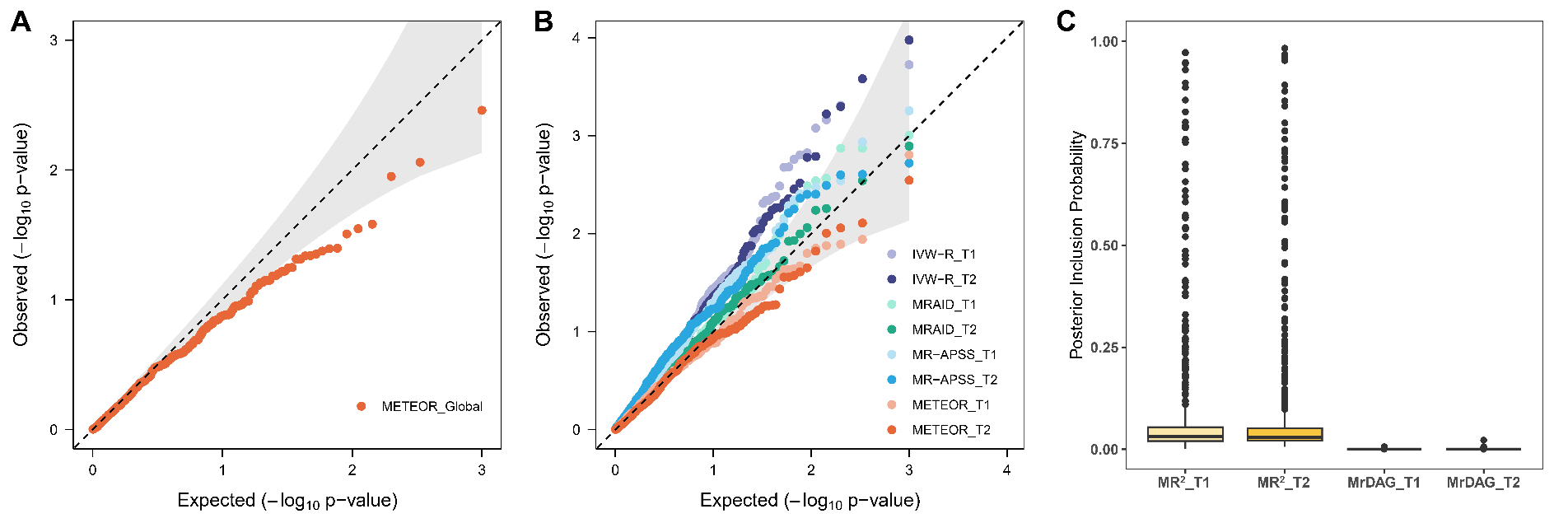


Supplementary Fig 15 Results from simulations with various proportions of sample overlap along with various sample sizes. Scenario with sample sizes for both exposure and outcomes being 100,000 and with 100% sample overlap. These scenarios involve one exposure and two outcomes, with the following parameters: $PVE_{\tilde{G}_{1}}=10\%$, $K=100$, $\pi_{1k}=20\%$, $PVE_{hk}=5\%$ ($k=1,2$) , $\tilde{\rho}_{x,y_{1}}=\tilde{\rho}_{x,y_{2}}=0$, and $PVE_{\alpha}=\left( 0,0 \right)^{T}$. (A) QQ plots from the global test of METEOR. (B) QQ plots from IVW-R, MRAID, MR-APSS and METEOR in testing the causal effects of exposure on the both outcomes. (C) Posterior inclusion probabilities (PIPs) from MR^2^ and MrDAG for two outcomes (T1 and T2) in null simulations.


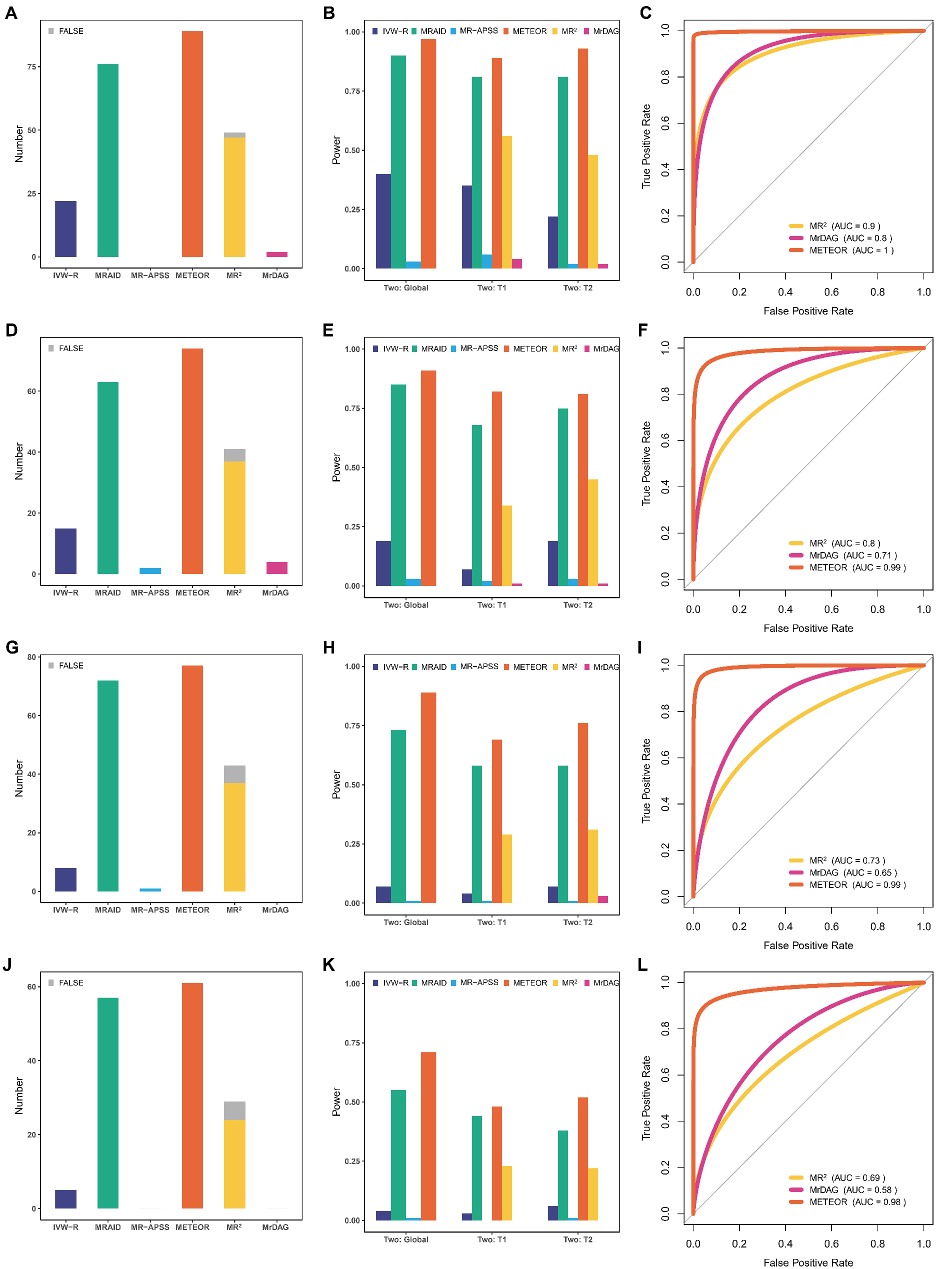


Supplementary Figure 16 Simulations with various $\pi_{1k}$. The scenario involves one exposure and two outcomes (T1 and T2), with the following parameters: $PVE_{\tilde{G}_{1}}=10\%$, $K=100$, $PVE_{hk}=5\%$, $n_{1}=n_{2k}=50,000$ ($k=1,2$), $\tilde{\rho}_{y_{1},y_{2}}=0.5$ and $\tilde{\rho}_{x,y_{1}}=\tilde{\rho}_{x,y_{2}}=0$. Four values of $\pi_{1k}$ are considered: 0.1, 0.2, 0.3, and 0.4, listed from left to right. (A, D, G, J) Numbers of true discovery and false discovery (grey) for all methods in the baseline setting with $\boldsymbol{PV}\boldsymbol{E}_{\alpha}=\left( 0.075\%,0 \right)^{T}$. (B, E, H, K) The results are plotted for one alternative scenario: $\boldsymbol{PV}\boldsymbol{E}_{\alpha}=\left( 0.075\%,0.075\% \right)^{T}$. (C, F, I, L) ROC curves for MR^2^ , MrDAG and METEOR.


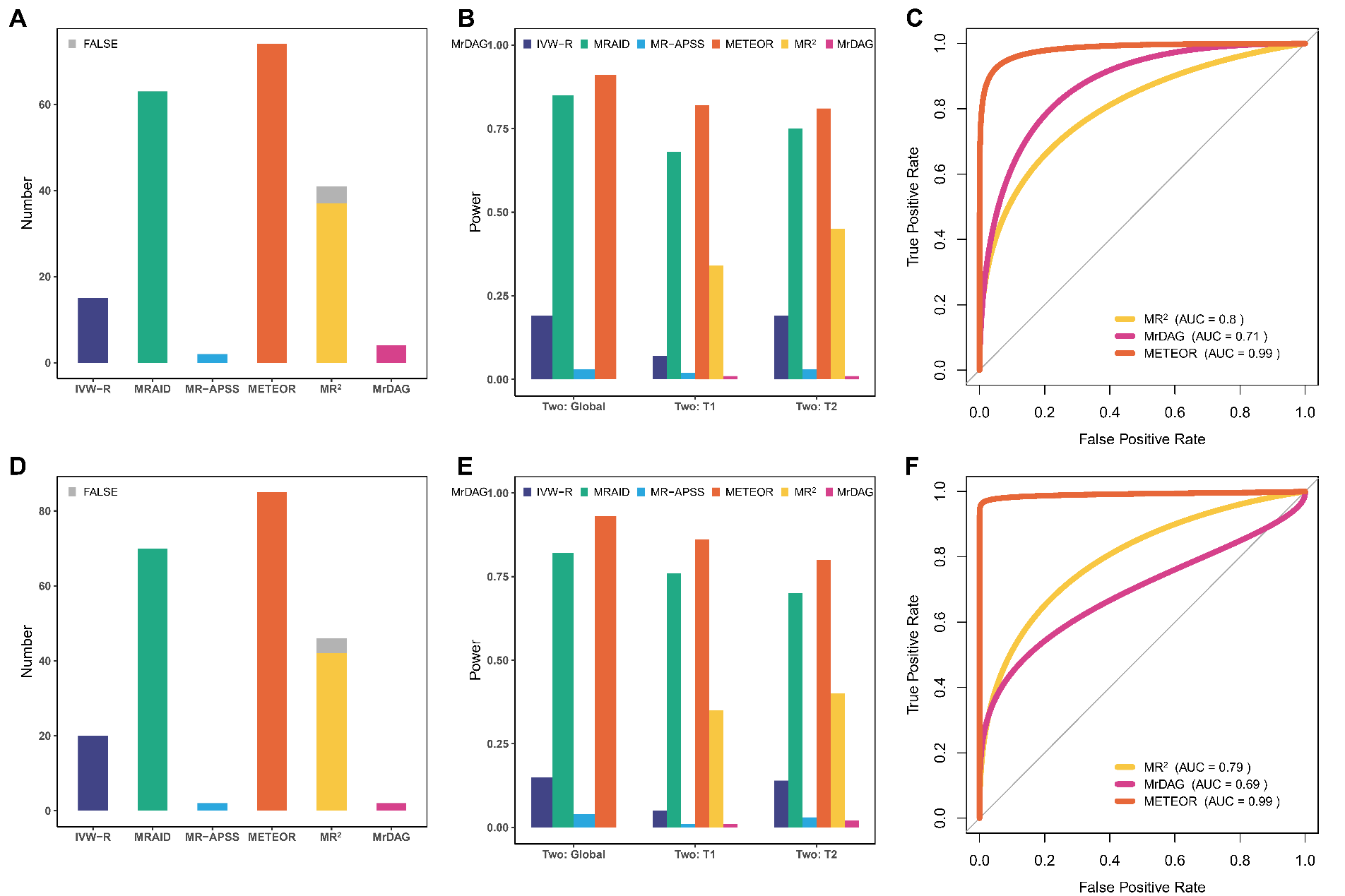


Supplementary Figure 17 Power and Receiver operating characteristic (ROC) curves from simulations with various $PVE_{\tilde{G}_{1}}$. The scenario involves one exposure and two outcomes (T1 and T2), with the following parameters: $K=100$, $\pi_{1k}=20\%$, $PVE_{hk}=5\%$, $n_{1}=n_{2k}=50,000$ ($k=1,2$), $\tilde{\rho}_{y_{1},y_{2}}=0.5$ and $\tilde{\rho}_{x,y_{1}}=\tilde{\rho}_{x,y_{2}}=0$. In alternative simulations, $PVE_{\alpha k}=0.075\%$. Power performance under Bonferroni adjusted $p$-value threshold of $5\times{10}^{-4}$ for global and single tests. (A, D) Numbers of true discovery and false discovery (grey) for all methods in the baseline setting with $\boldsymbol{PV}\boldsymbol{E}_{\alpha}=\left( 0.075\%,0 \right)^{T}$. (B, E) The results are plotted for one alternative scenario: $\boldsymbol{PV}\boldsymbol{E}_{\alpha}=\left( 0.075\%,0.075\% \right)^{T}$. (C, F) ROC curves for MR^2^, MrDAG and METEOR by plotting the true positive rate (TPR) against the false positive rate (FPR) in null simulations. Four values of $PVE_{\tilde{G}_{1}}$ are considered: 10% and 15%, listed from left to right.


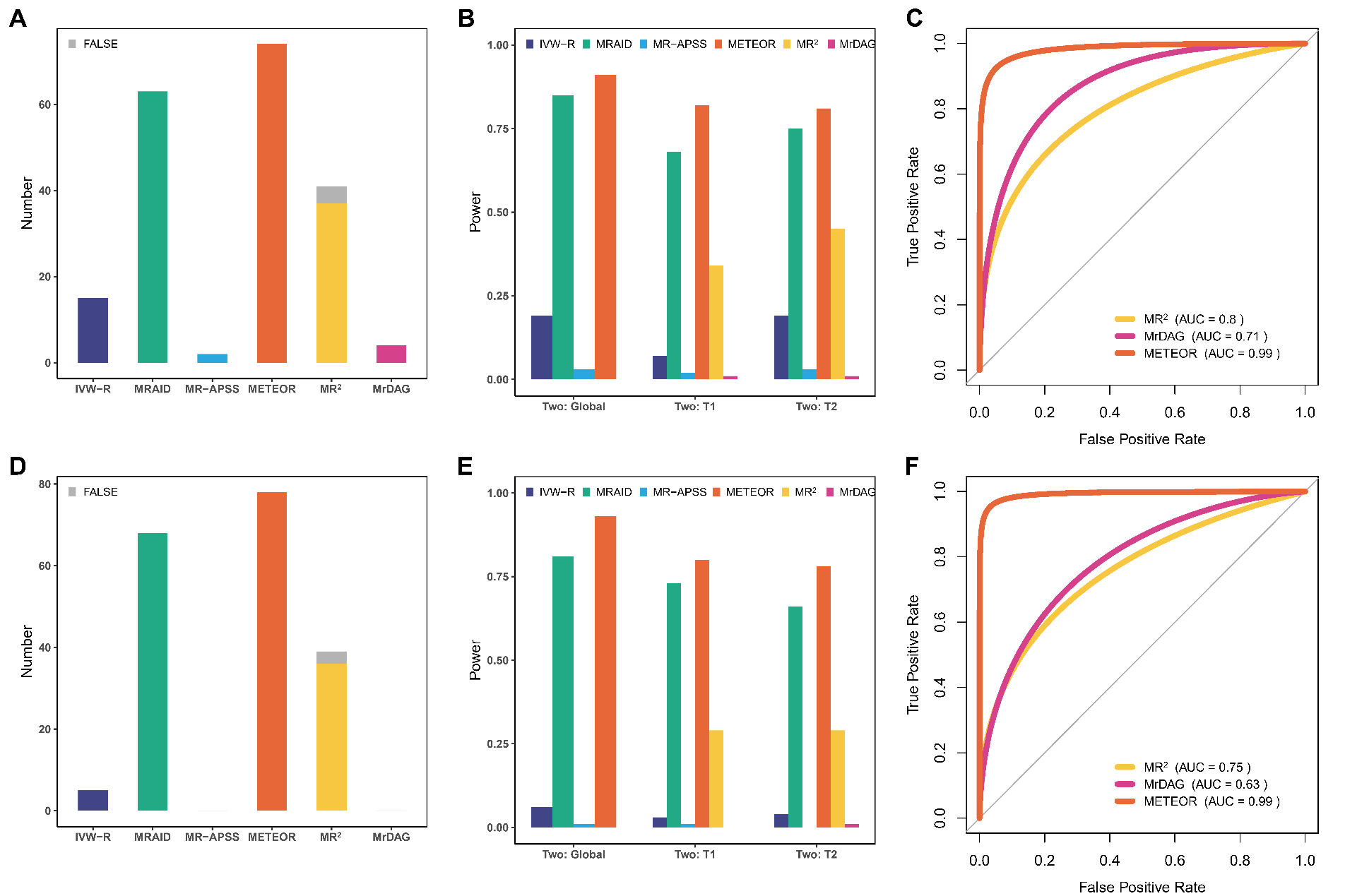


Supplementary Figure 18 Power and Receiver operating characteristic (ROC) curves from simulations with various $PVE_{hk}$. The scenario involves one exposure and two outcomes (T1 and T2), with the following parameters: $PVE_{\tilde{G}_{1}}=10\%$, $K=100$, $\pi_{1k}=20\%$, $n_{1}=n_{2k}=50,000$ (k=1,2), $\tilde{\rho}_{y_{1},y_{2}}=0.5$ and $\tilde{\rho}_{x,y_{1}}=\tilde{\rho}_{x,y_{2}}=0$. Four values of PVE_hk are considered: 5% and 10%, listed from left to right. Power performance under Bonferroni adjusted $p$-value threshold of $5\times{10}^{-4}$ for global and single tests. (A, D) Numbers of true discovery and false discovery (grey) for all methods in the baseline setting with $\boldsymbol{PV}\boldsymbol{E}_{\alpha}=\left( 0.075\%,0 \right)^{T}$. (B, E) The results are plotted for one alternative scenario: $\boldsymbol{PV}\boldsymbol{E}_{\alpha}=\left( 0.075\%,0.075\% \right)^{T}$. (C, F) ROC curves for MR^2^ , MrDAG and METEOR by plotting the true positive rate (TPR) against the false positive rate (FPR) in the baseline setting.


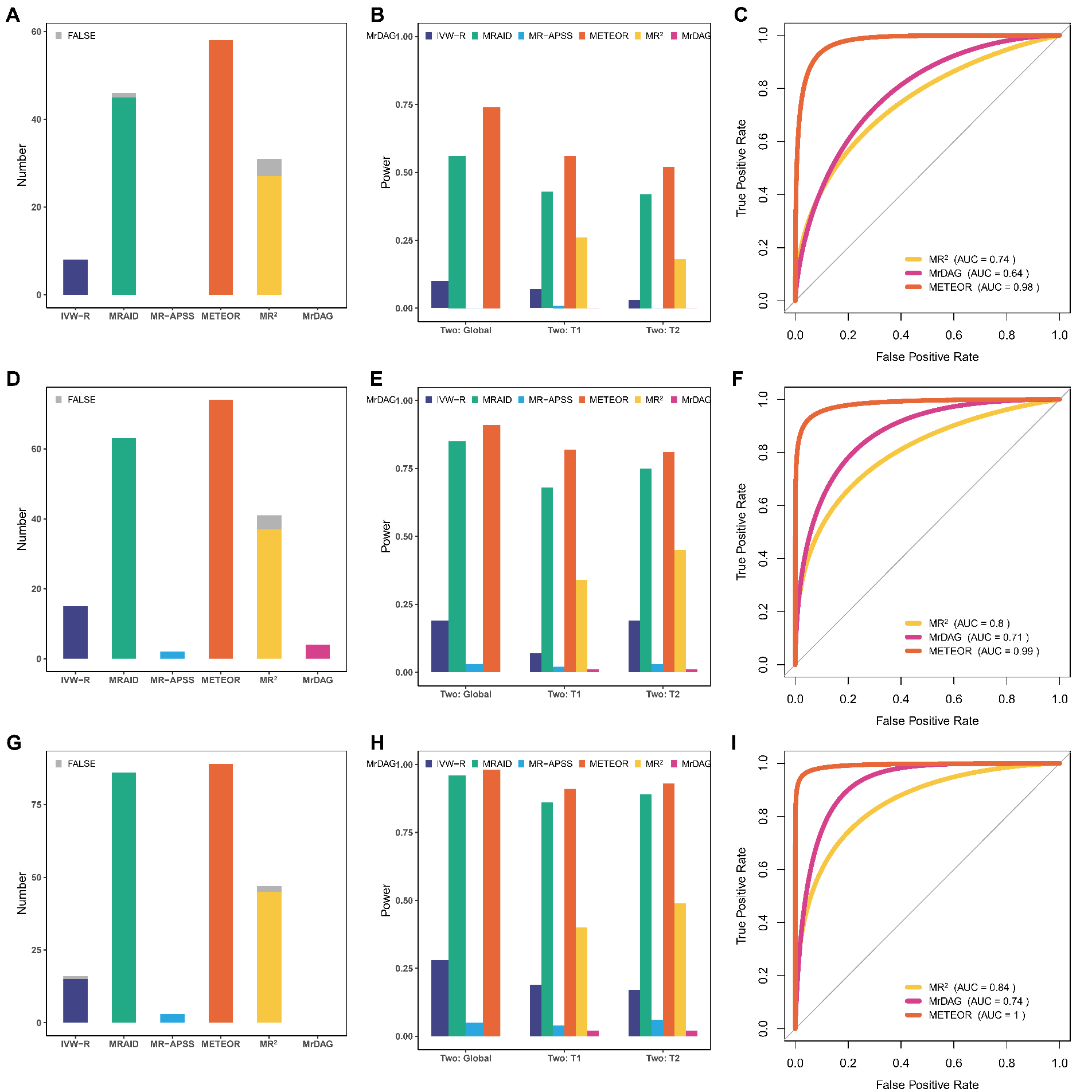


Supplementary Figure 19 Power from simulations with various $PVE_{\alpha k}$ when sample sizes for both exposure and outcomes are 50,000. The scenario involves one exposure and two outcomes (T1 and T2), with the following parameters: $PVE_{\tilde{G}_{1}}=10\%$, $K=100$, $\pi_{1k}=20\%$, $PVE_{hk}=5\%$ ($k=1,2$), $\tilde{\rho}_{y_{1},y_{2}}=0.5$ and $\tilde{\rho}_{x,y_{1}}=\tilde{\rho}_{x,y_{2}}=0$. Three values of $PVE_{\alpha k}$ are considered from top to bottom: 0.05%, 0.75% and 0.1%. Power performance under Bonferroni adjusted $p$-value threshold of $5\times{10}^{-4}$ for global and single tests. (A, D, G) Numbers of true discovery and false discovery (grey) for all methods in the baseline setting with one zero $PVE_{\alpha k}$ and one non-zero $PVE_{\alpha k}$. (B, E, H) The results are plotted for one alternative scenario with both non-zero $PVE_{\alpha k}$. (C, F, I) Receiver operating characteristic (ROC) curves for MR^2^ , MrDAG and METEOR.


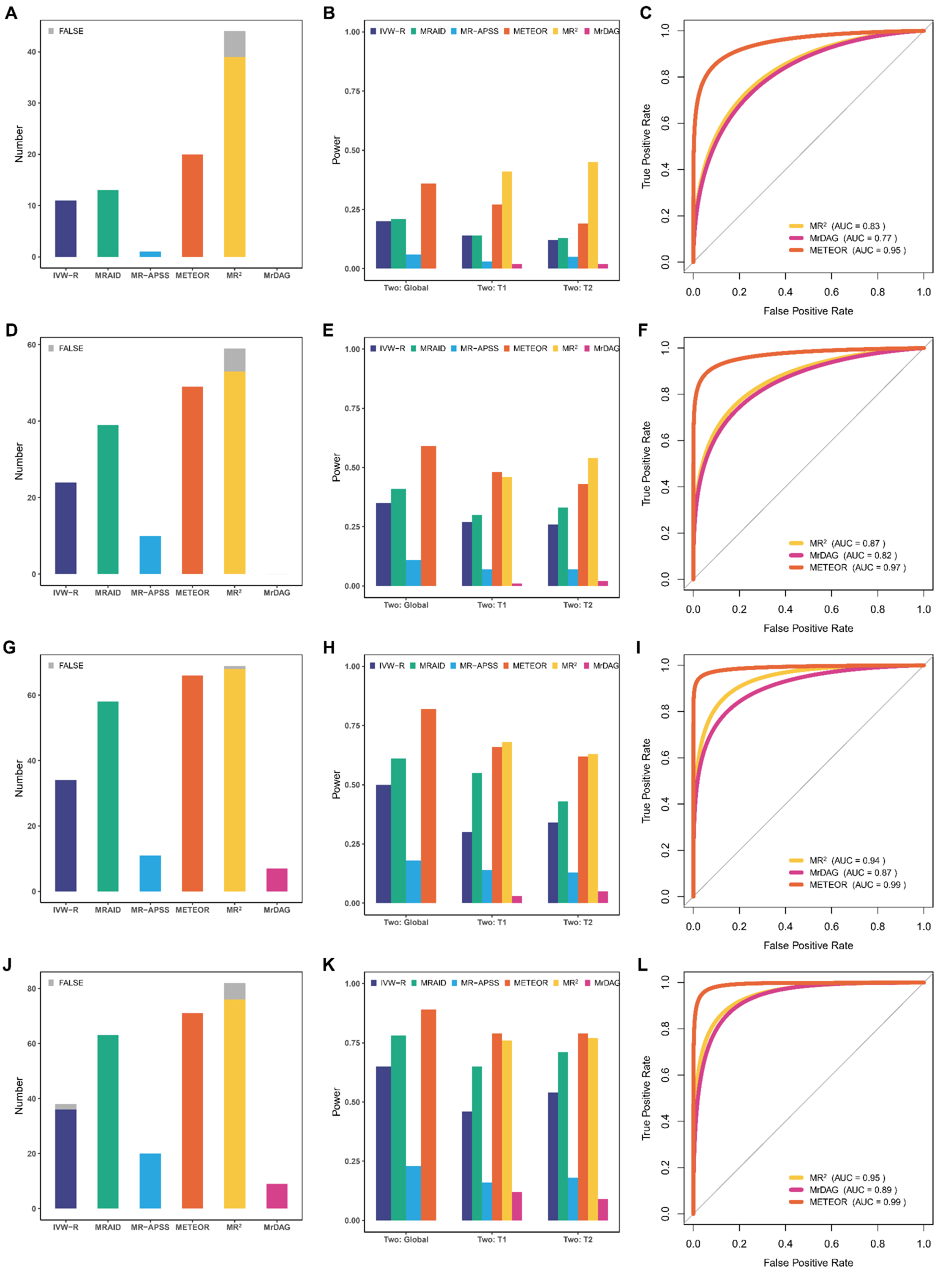


Supplementary Figure 20 Power from simulations with various $PVE_{\alpha k}$ when sample sizes for both exposure and outcomes are 20,000. Four values of $PVE_{\alpha k}$ are considered from top to bottom: 0.1%, 0.15%, 0.2%. (A, D, G, J) Numbers of true discovery and false discovery (grey) for all methods in the baseline setting with one zero $PVE_{\alpha k}$ and one non-zero $PVE_{\alpha k}$ under Bonferroni adjusted $p$-value threshold of $5\times{10}^{-4}$. (B, E, H, K) The results are plotted for one alternative scenario with both non-zero $PVE_{\alpha k}$. (C, F, I, L) Receiver operating characteristic (ROC) curves for MR^2^ , MrDAG and METEOR.


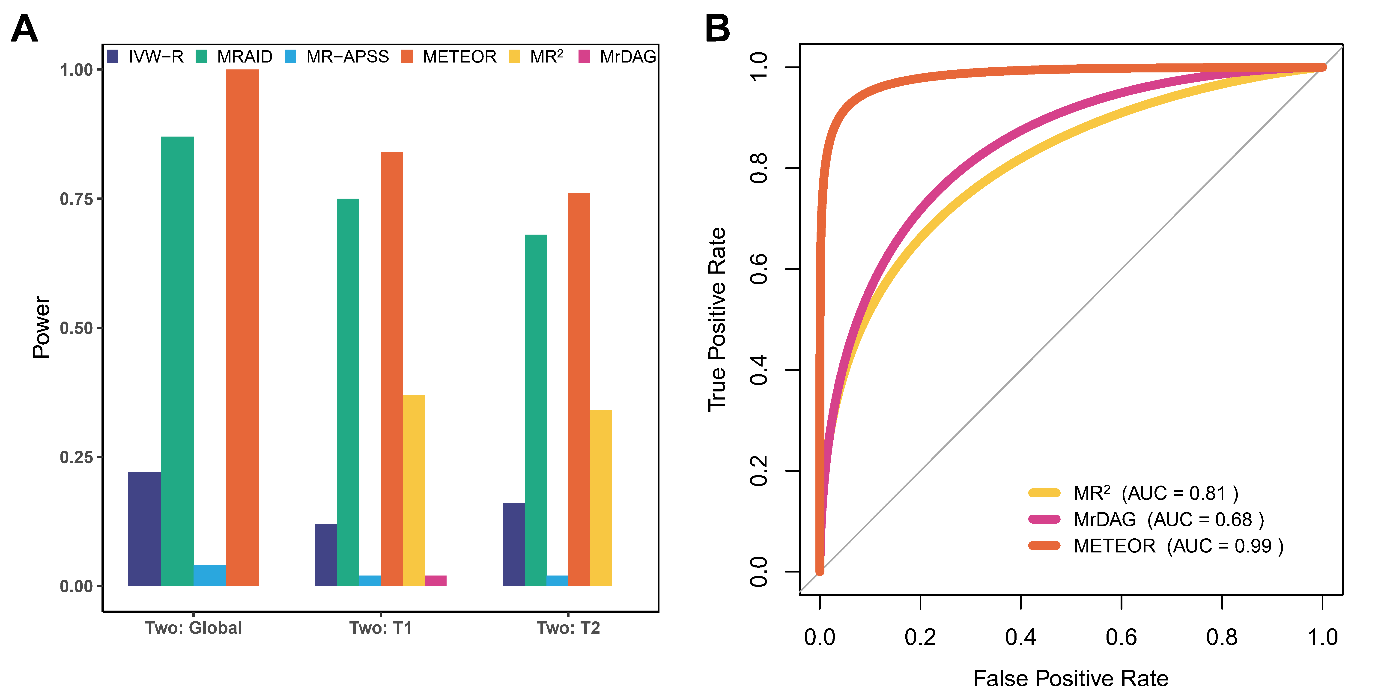


Supplementary Figure 21 Simulation that the exposure causally affects both traits with opposite effect sizes. The scenario involves one exposure and two outcomes (T1 and T2), with the following parameters: $PVE_{\tilde{G}_{1}}=10\%$, $K=100$, $\pi_{1k}=20\%$, $PVE_{hk}=5\%$, $n_{1}=n_{2k}=50,000$ ($k=1,2$), $\tilde{\rho}_{y_{1},y_{2}}=0.5$ and $\tilde{\rho}_{x,y_{1}}=\tilde{\rho}_{x,y_{2}}=0$. (A) The results are plotted for setting where the exposure causally affects both traits with effect sizes being 0.087 and -0.087. (B) Receiver operating characteristic (ROC) curves for MR^2^ , MrDAG and METEOR.

*
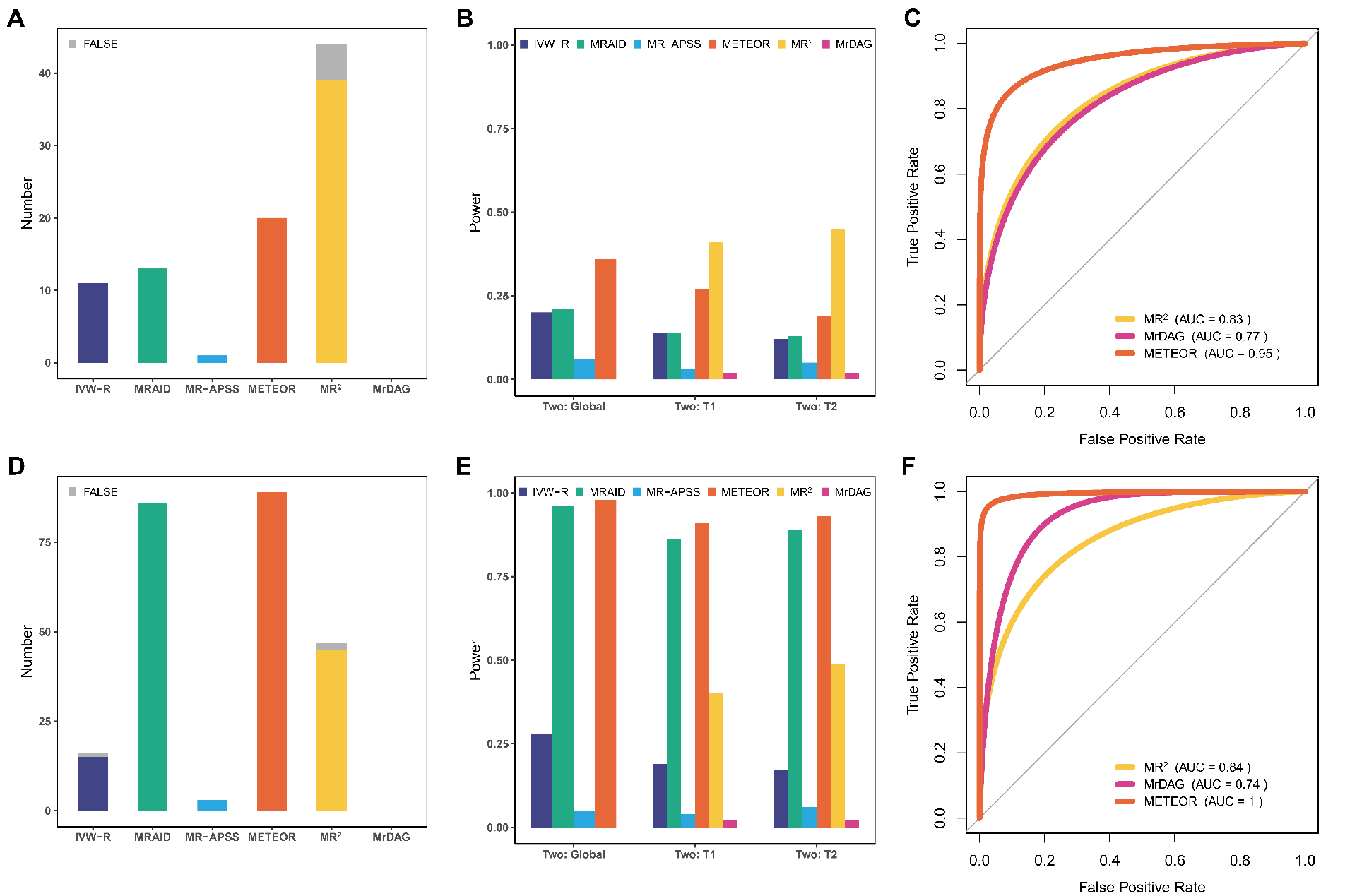
*

Supplementary Figure 22 Power from simulations with various sample sizes for both exposure and outcomes when $PVE_{\alpha k}$ is $0.1\%$. The scenario involves one exposure and two outcomes (T1 and T2), with the following parameters: $PVE_{\tilde{G}_{1}}=10\%$, $K=100$, $\pi_{1k}=20\%$, $PVE_{hk}=5\%$ ($k=1,2$), $\tilde{\rho}_{y_{1},y_{2}}=0.5$ and $\tilde{\rho}_{x,y_{1}}=\tilde{\rho}_{x,y_{2}}=0$. Two sample sizes are considered: (A-C) 20,000, and (D-F) 50,000. (A, D) Numbers of true discovery and false discovery (grey) for all methods in the baseline setting with $PVE_{\alpha}=\left( 0.1\%,0 \right)^{T}$. (B, E) The results are plotted for one alternative scenario: $PVE_{\alpha}=\left( 0.1\%,0.1\% \right)^{T}$. (C, F) Receiver operating characteristic (ROC) curves for IVW-R, MRAID and METEOR, plotting the true positive rate (TPR) against the false positive rate (FPR).


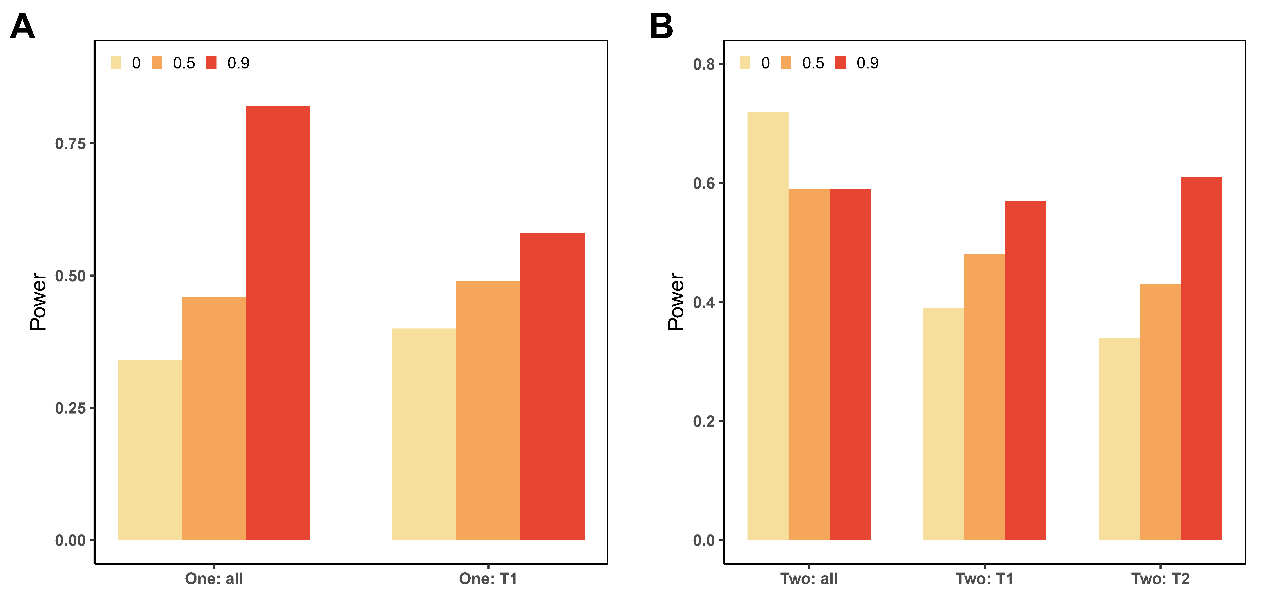


Supplementary Figure 23 Power from simulations with various correlations when sample sizes for both exposure and outcomes are 20,000. The scenario involves one exposure and two outcomes (T1 and T2), with the following parameters: $PVE_{\tilde{G}_{1}}=10\%$, $K=100$, $\pi_{1k}=20\%$, $PVE_{hk}=5\%$ ($k=1,2$), and $\tilde{\rho}_{x,y_{1}}=\tilde{\rho}_{x,y_{2}}=0$. Three correlation values between any two outcomes are considered, including 0, 0.5 and 0.9. Power performance under Bonferroni adjusted $p$-value threshold of $5\times{10}^{-4}$ for global and single tests. Results from METEOR are plotted for two alternative settings: (A) $\boldsymbol{PV}\boldsymbol{E}_{\alpha}=\left( 0.15\%,0 \right)^{T}$ and (B) $\boldsymbol{PV}\boldsymbol{E}_{\alpha}=\left( 0.15\%,0.15\% \right)^{T}$


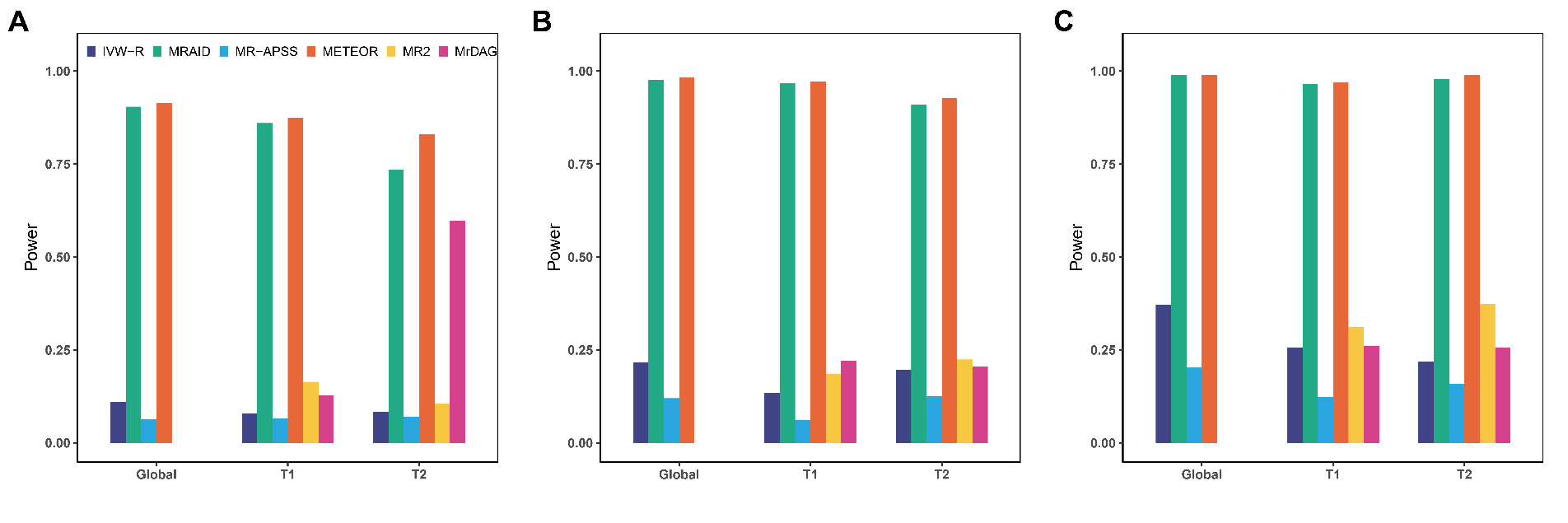


Supplementary Figure 24 Power at an FDR of 0.05 of different methods for global and single tests in baseline setting. The scenario involves one exposure and two outcomes (T1 and T2), with the following parameters: $PVE_{\tilde{G}_{1}}=10\%$, $K=100$, $\pi_{1k}=20\%$, $PVE_{hk}=5\%$ ($k=1,2$), $\tilde{\rho}_{y_{1},y_{2}}=0.5$ and $\tilde{\rho}_{x,y_{1}}=\tilde{\rho}_{x,y_{2}}=0$. The results are plotted forone alternative setting where the exposure causally affects both traits (Two: $\boldsymbol{PV}\boldsymbol{E}_{\alpha}=(PVE_{\alpha1},PVE_{\alpha2})$ and $PVE_{\alpha1}=PVE_{\alpha2}$). Three non-zero $PVE_{\alpha k}$ are considered: (A) $0.05\%$, (B) $0.075\%$ and (C) $0.1\%$.


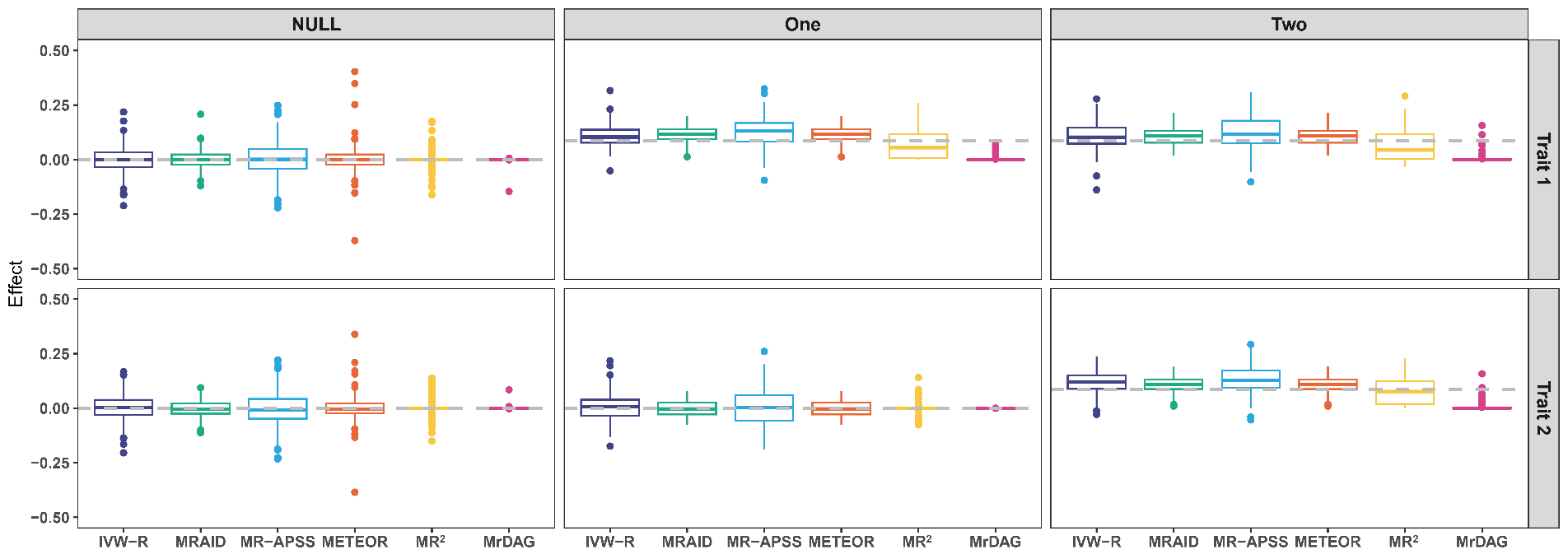


Supplementary Figure 25 Estimates of causal effects from five MR methods in simulations with sample sizes of 20,000 for both exposure and outcomes. The scenario involves one exposure and two outcomes, with the following parameters: $PVE_{\tilde{G}_{1}}=10\%$, $K=100$, $\pi_{1k}=20\%$, $PVE_{hk}=5\%$ ($k=1,2$), $\tilde{\rho}_{y_{1},y_{2}}=0.5$ and $\tilde{\rho}_{x,y_{1}}=\tilde{\rho}_{x,y_{2}}=0$. Three causal effect settings are considered, listed from left to right: (1) a null setting where the exposure has no effect on any outcome with $\boldsymbol{PV}\boldsymbol{E}_{\alpha}=\left( 0,0 \right)^{T}$; (2) an alternative setting where the exposure causally affects one outcome with $\boldsymbol{PV}\boldsymbol{E}_{\alpha}=\left( 0.15\%,0 \right)^{T}$ and (3) a alternative setting where the exposure causally affects both outcomes with $\boldsymbol{PV}\boldsymbol{E}_{\alpha}=\left( 0.15\%,0.15\% \right)^{T}$. The causal effect estimates for the first and second outcomes are listed from top to bottom.


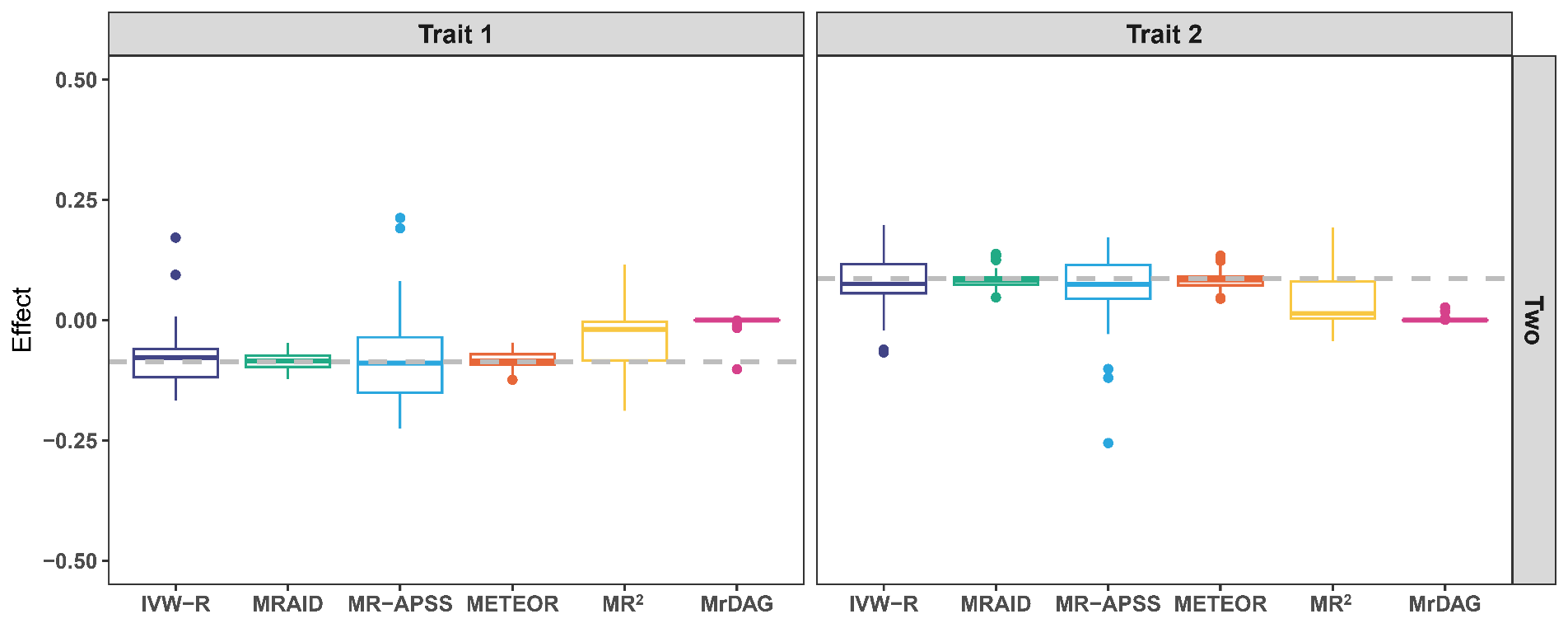


Supplementary Figure 26 Estimates of causal effects from six MR methods in simulations with sample sizes of 50,000 for both exposure and outcomes with different causal effects. The scenario involves one exposure and two outcomes, with the following parameters: $PVE_{\tilde{G}_{1}}=10\%$, $K=100$, $\pi_{1k}=20\%$, $PVE_{hk}=5\%$ ($k=1,2$), $\tilde{\rho}_{y_{1},y_{2}}=0.5$, $\tilde{\rho}_{x,y_{1}}=\tilde{\rho}_{x,y_{2}}=0$, $\boldsymbol{PV}\boldsymbol{E}_{\alpha}=\left( 0.075\%,0.075\% \right)^{T}$, $\alpha_{1}=-0.087$ (left) and $\alpha_{2}=0.087$ (right).


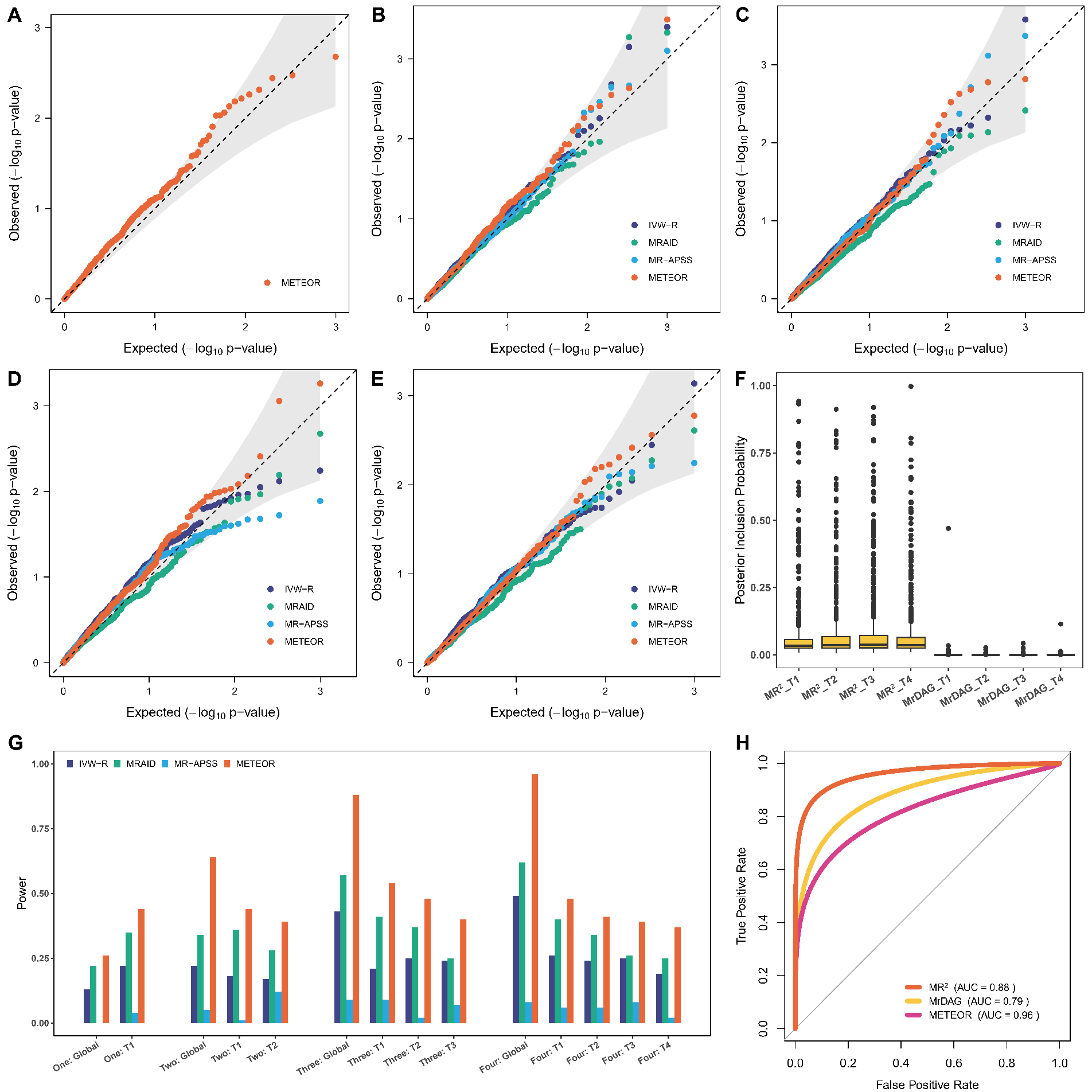


Supplementary Figure 27 Results from a scenario with no sample overlap and no correlation. The scenario includes one exposure and four outcomes, each drawn from entirely distinct datasets with no sample overlap, and the following parameters: $PVE_{\tilde{G}_{1}}=10\%$, $K=100$, $\pi_{1k}=20\%$, $PVE_{hk}=5\%$, $n_{1}=n_{2k}=20,000 (k=1,\cdots,4)$, with a correlation of 0 between any two traits. In alternative simulations, $PVE_{\alpha k}=0.15\%$. Type I error control is evaluated using quantile-quantile (QQ) plots of $-\log_{10} p$ values in null simulations. (A) QQ plot from global test of METEOR. QQ plots from IVW-R, MRAID, MR-APSS and METEOR in testing the causal effects of exposure on (B) the first, (C) second, (D) third and (E) fourth outcome. (F) Posterior inclusion probabilities (PIPs) from MR^2^ and MrDAG for the two outcomes (T1 and T2) in the baseline setting. (G) Power performance under Bonferroni adjusted $p$-value threshold of $5\times{10}^{-4}$ for global and single tests. The results from IVW-R, MRAID, MR-APSS and METEOR are plotted for four alternative scenarios: ranging from a setting where the exposure causally affects one trait to a setting where the exposure causally affects four traits. (H) Receiver operating characteristic (ROC) curves for MR^2^ , MrDAG and METEOR by plotting the true positive rate (TPR) against the false positive rate (FPR).


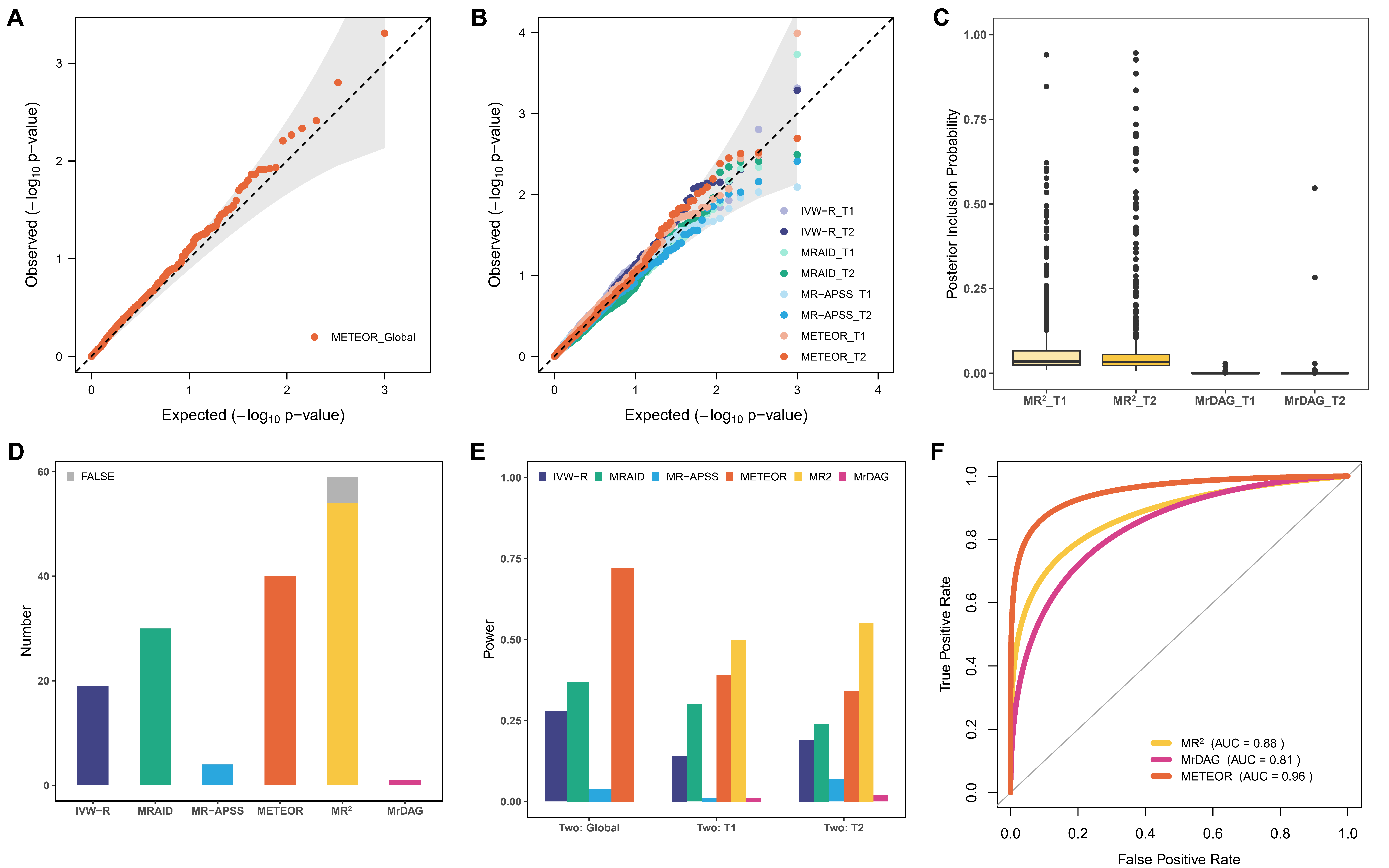


Supplementary Figure 28 Results from a scenario with sample overlap but no correlation in two sample MR setting. The scenario involves one exposure drawn from one dataset and two outcomes drawn from another dataset, with the following parameters: $PVE_{\tilde{G}_{1}}=10\%$, $K=100$, $\pi_{1k}=20\%$, $PVE_{hk}=5\%$, $n_{1}=n_{2k}=20,000 (k=1,2)$, with a correlation of 0 between any two traits. Type I error control is evaluated using quantile-quantile (QQ) plots of $-\log_{10} p$ values in null simulations. (A) QQ plot from global test of METEOR. (B) QQ plots from IVW-R, MRAID, and METEOR in testing the causal effects of exposure on the both outcomes. (C) Posterior inclusion probabilities (PIPs) from MR^2^ and MrDAG for the two outcomes (T1 and T2) in the baseline setting. Power performance under Bonferroni adjusted $p$-value threshold of $5\times{10}^{-4}$ for global and single tests. (D) Numbers of true discovery and false discover (grey) for all methods in the baseline setting with $\boldsymbol{PV}\boldsymbol{E}_{\alpha}=\left( 0.15\%,0 \right)^{T}$. (E) The results are plotted for one alternative scenario with $\boldsymbol{PV}\boldsymbol{E}_{\alpha}=\left( 0.15\%,0.15\% \right)^{T}$. (F) Receiver operating characteristic (ROC) curves for MR^2^ , MrDAG and METEOR by plotting the true positive rate (TPR) against the false positive rate (FPR).


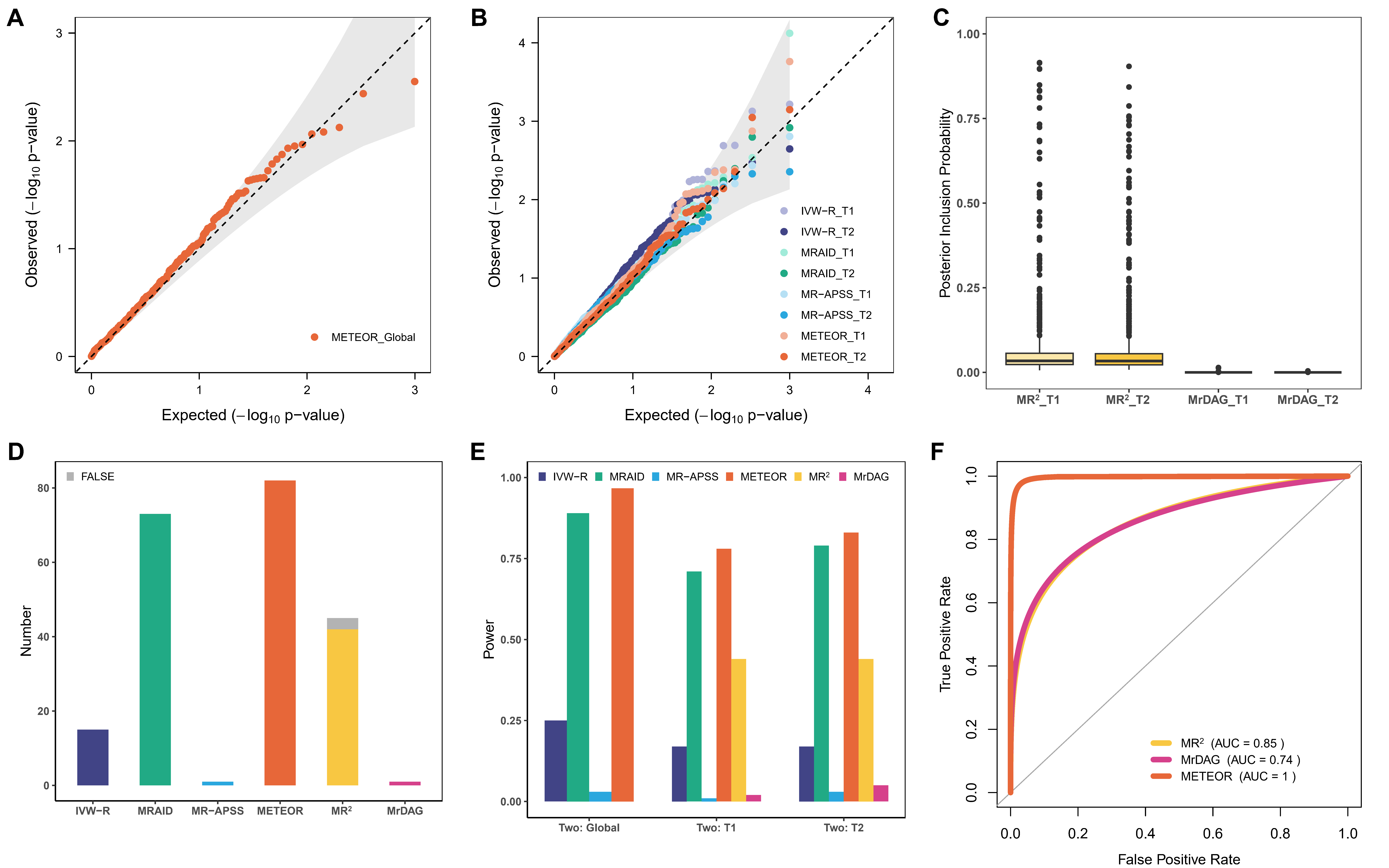


Supplementary Figure 29 Results from a scenario with sample overlap but no correlation in one sample MR setting. The scenario involves one exposure and two outcomes, all drawn from the same dataset, with the following parameters: $PVE_{\tilde{G}_{1}}=10\%$, $K=100$, $\pi_{1k}=20\%$, $PVE_{hk}=5\%$, $n_{1}=n_{2k}=50,000 (k=1,2)$,with a correlation of 0 between any two traits. Type I error control is evaluated using quantile-quantile (QQ) plots of $-\log_{10} p$ values in null simulations. (A) QQ plot from global test of METEOR. (B) QQ plots from IVW-R, MRAID, MR-APSS and METEOR in testing the causal effects of exposure on both outcomes (T1 and T2). (D) Posterior inclusion probabilities (PIPs) from MR^2^ and MrDAG for the two outcomes in the baseline setting. Power performance under Bonferroni adjusted $p$-value threshold of $5\times{10}^{-4}$ for global and single tests. (D) Numbers of true discovery and false discover (grey) for all methods in the baseline setting with $\boldsymbol{PV}\boldsymbol{E}_{\alpha}=\left( 0.075\%,0 \right)^{T}$. (E) The results are plotted for one alternative scenario with $\boldsymbol{PV}\boldsymbol{E}_{\alpha}=\left( 0.075\%,0.075\% \right)^{T}$. (F) Receiver operating characteristic (ROC) curves for MR^2^ , MrDAG and METEOR by plotting the true positive rate (TPR) against the false positive rate (FPR).


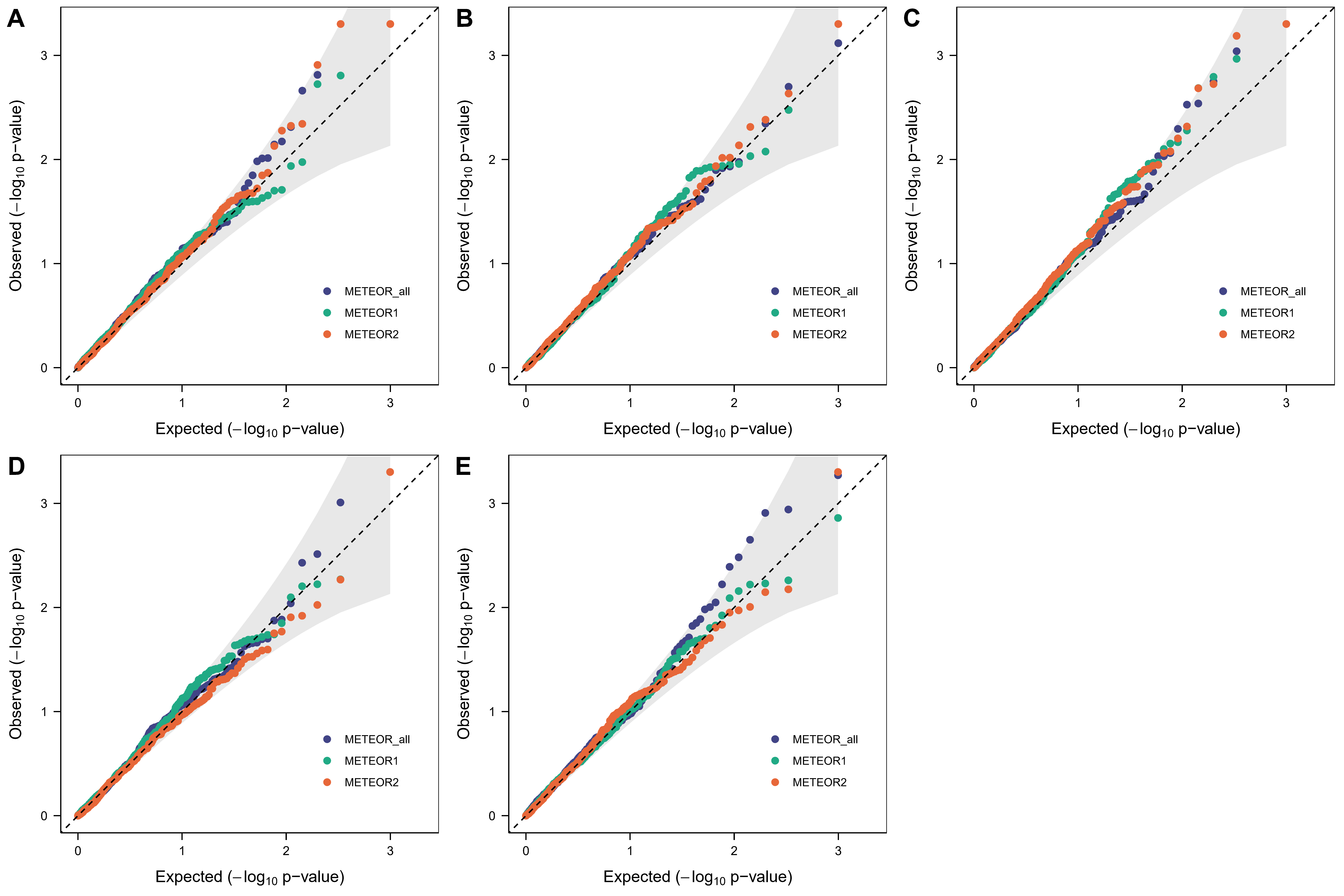


Supplementary Figure 30 Quantile-quantile (QQ) plots of METEOR in scenarios with sample overlap and correlation. The scenario involves one exposure and two outcomes, with the following parameters: $PVE_{\tilde{G}_{1}}=10\%$, $K=100$, $\pi_{1k}=20\%$, $PVE_{hk}=5\%$, $n_{1}=n_{2k}=50,000 (k=1,2)$. Type I error control is evaluated by quantile-quantile (QQ) plots of $-\log_{10} p$ from METEOR in testing overall test (blue), and in testing the single tests on the first (green) and (orange) the second outcome in null simulations. Three scenarios were considered: the exposure and both outcomes are from the same dataset, with $\tilde{\rho}_{u,v}$ being (A) 0.5, (B) 0.7 and (C) 0.9 for $u, v\in\left( x,y_{1},y_{2} \right)$; (D) the exposure and the first outcome are from the same dataset, with $\tilde{\rho}_{x,y_{1}}=0.5$, while the second outcome is from another dataset, with $\tilde{\rho}_{x,y_{2}}=\tilde{\rho}_{y_{1},y_{2}}=0$; (E) the exposure and the second outcome are from the same dataset, with $\tilde{\rho}_{x,y_{2}}=0.5$, while the first outcome is from another dataset, with $\tilde{\rho}_{x,y_{1}}=\tilde{\rho}_{y_{1},y_{2}}=0$.


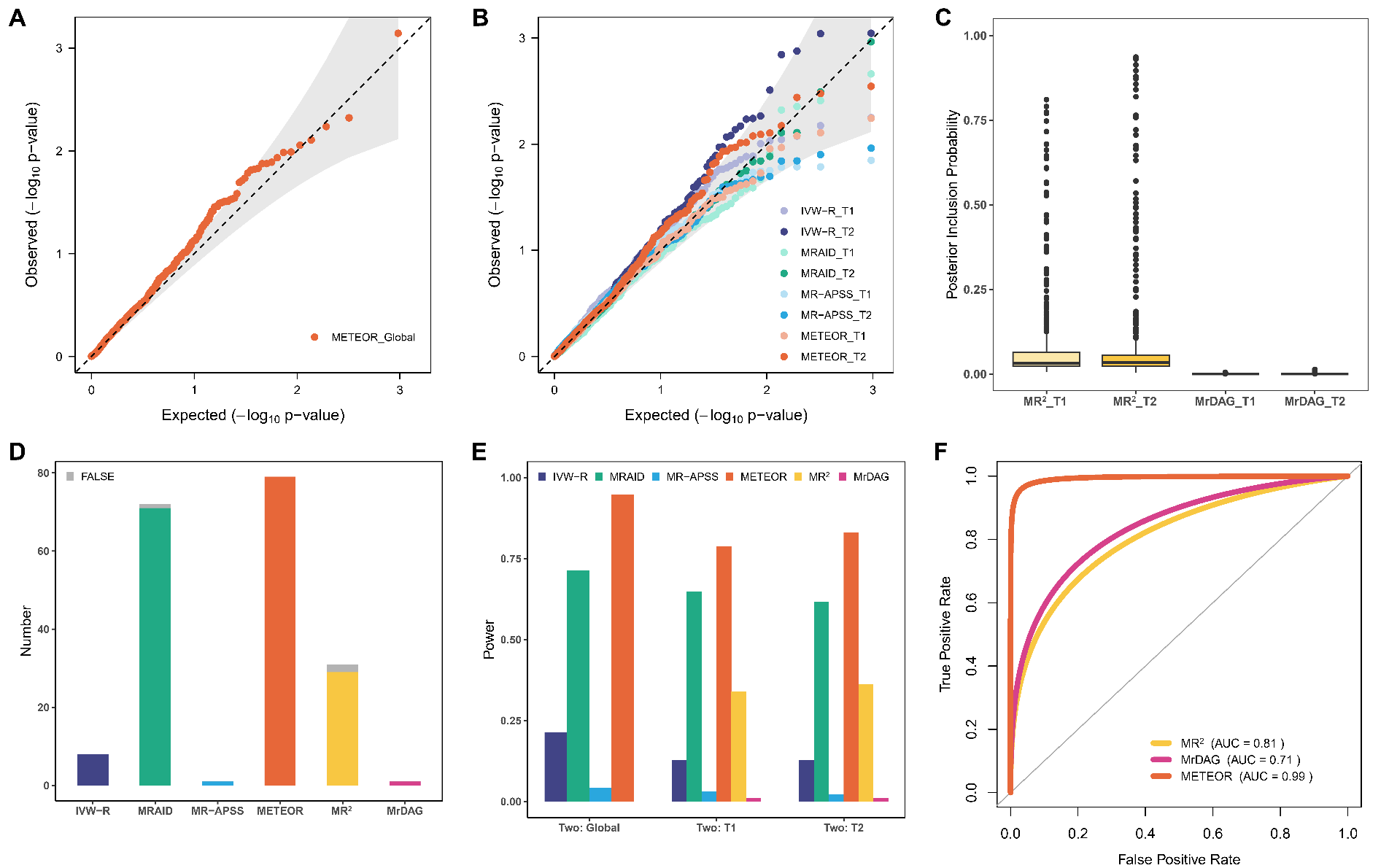


**Supplementary Figure 31** Results under the baseline scenario where exposure residuals follow a t distribution with 30 degrees of freedom. (A) QQ plot from the global test of METEOR in the null simulations. (B) QQ plots from IVW-R, MRAID, MR-APSS and METEOR in testing the causal effects of exposure on the both outcomes (T1 and T2) in null simulations. (C) Posterior inclusion probabilities (PIPs) from MR^2^ and MrDAG for the two outcomes in the null simulations. Power performance under Bonferroni adjusted $p$-value threshold of $5\times{10}^{-4}$ for global and single tests. (D) Numbers of true discovery and false discover (grey) for all methods in the baseline setting with $\boldsymbol{PV}\boldsymbol{E}_{\alpha}=\left( 0.075\%,0 \right)^{T}$. (E) The results are plotted under $\boldsymbol{PV}\boldsymbol{E}_{\alpha}=\left( 0.075\%,0.075\% \right)^{T}$ (‘Two: Global’ for global test; ‘Two: T1’ and ‘Two: T2’ for single tests). (F) Receiver operating characteristic (ROC) curves for MR^2^, MrDAG and METEOR by plotting the true positive rate (TPR) against the false positive rate (FPR).


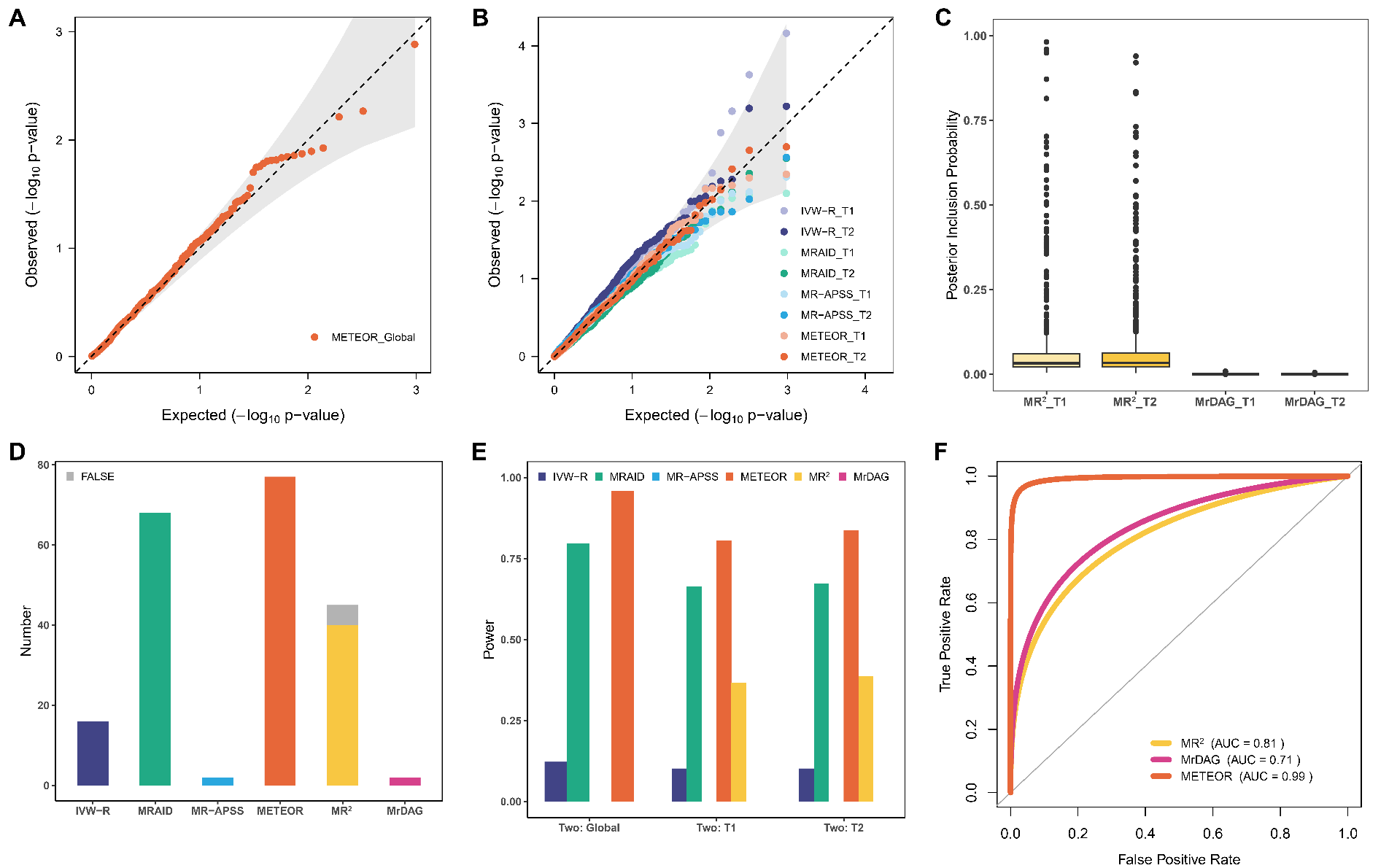


**Supplementary Figure 32** Results under the baseline scenario where exposure residuals follow a t distribution with 10 degrees of freedom. (A) QQ plot from the global test of METEOR in the null simulations. (B) QQ plots from IVW-R, MRAID, MR-APSS and METEOR in testing the causal effects of exposure on the both outcomes (T1 and T2) in null simulations. (C) Posterior inclusion probabilities (PIPs) from MR^2^ and MrDAG for the two outcomes in the null simulations. Power performance under Bonferroni adjusted $p$-value threshold of $5\times{10}^{-4}$ for global and single tests. (D) Numbers of true discovery and false discover (grey) for all methods in the baseline setting with $\boldsymbol{PV}\boldsymbol{E}_{\alpha}=\left( 0.075\%,0 \right)^{T}$. (E) The results are plotted under $\boldsymbol{PV}\boldsymbol{E}_{\alpha}=\left( 0.075\%,0.075\% \right)^{T}$ (‘Two: Global’ for global test; ‘Two: T1’ and ‘Two: T2’ for single tests). (F) Receiver operating characteristic (ROC) curves for MR^2^, MrDAG and METEOR by plotting the true positive rate (TPR) against the false positive rate (FPR).


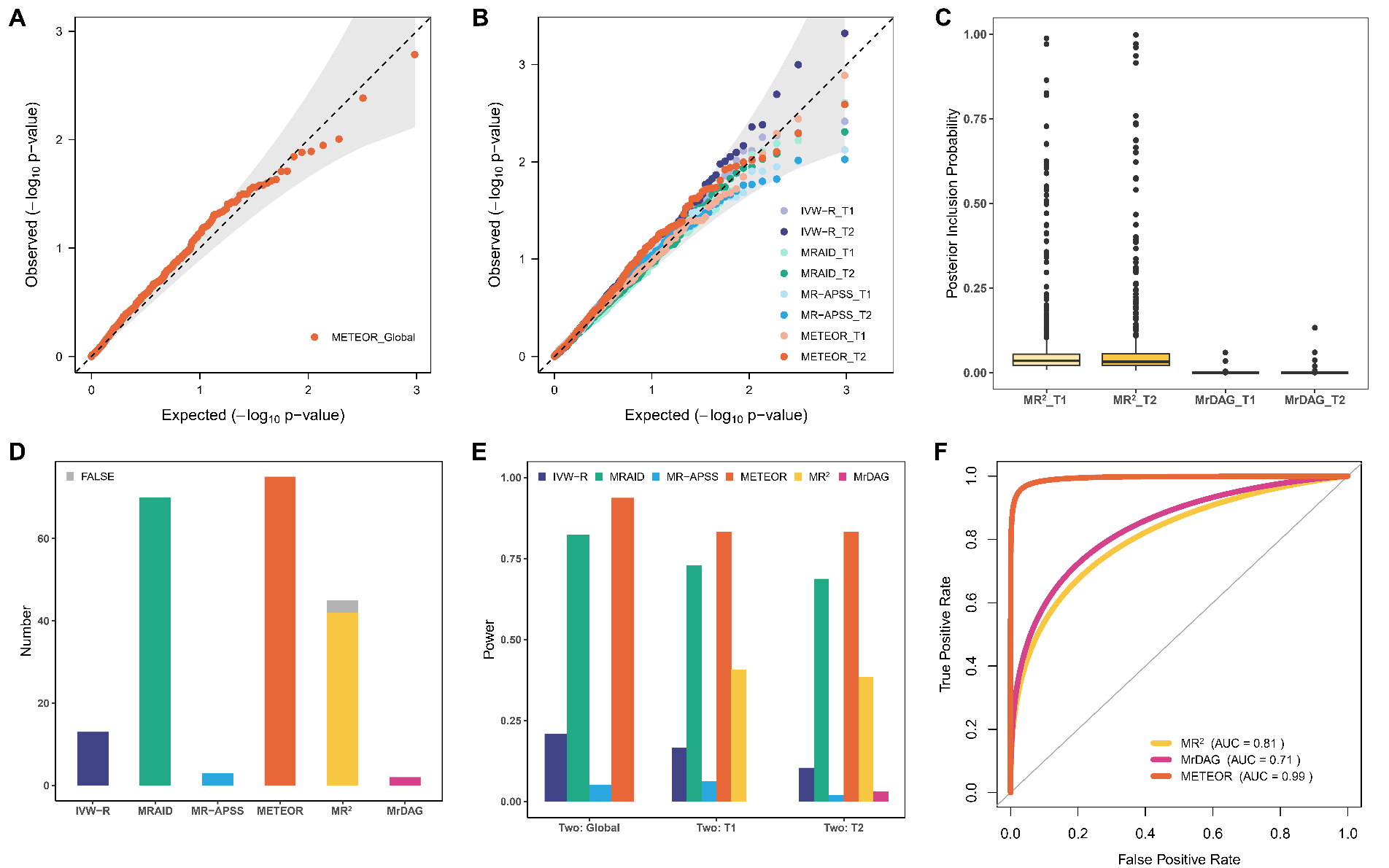


**Supplementary Figure 33** Results under the baseline scenario where exposure residuals follow a t distribution with 5 degrees of freedom. (A) QQ plot from the global test of METEOR in the null simulations. (B) QQ plots from IVW-R, MRAID, MR-APSS and METEOR in testing the causal effects of exposure on the both outcomes (T1 and T2) in null simulations. (C) Posterior inclusion probabilities (PIPs) from MR^2^ and MrDAG for the two outcomes in the null simulations. Power performance under Bonferroni adjusted $p$-value threshold of $5\times{10}^{-4}$ for global and single tests. (D) Numbers of true discovery and false discover (grey) for all methods in the baseline setting with $\boldsymbol{PV}\boldsymbol{E}_{\alpha}=\left( 0.075\%,0 \right)^{T}$. (E) The results are plotted under $\boldsymbol{PV}\boldsymbol{E}_{\alpha}=\left( 0.075\%,0.075\% \right)^{T}$ (‘Two: Global’ for global test; ‘Two: T1’ and ‘Two: T2’ for single tests). (F) Receiver operating characteristic (ROC) curves for MR^2^, MrDAG and METEOR by plotting the true positive rate (TPR) against the false positive rate (FPR).


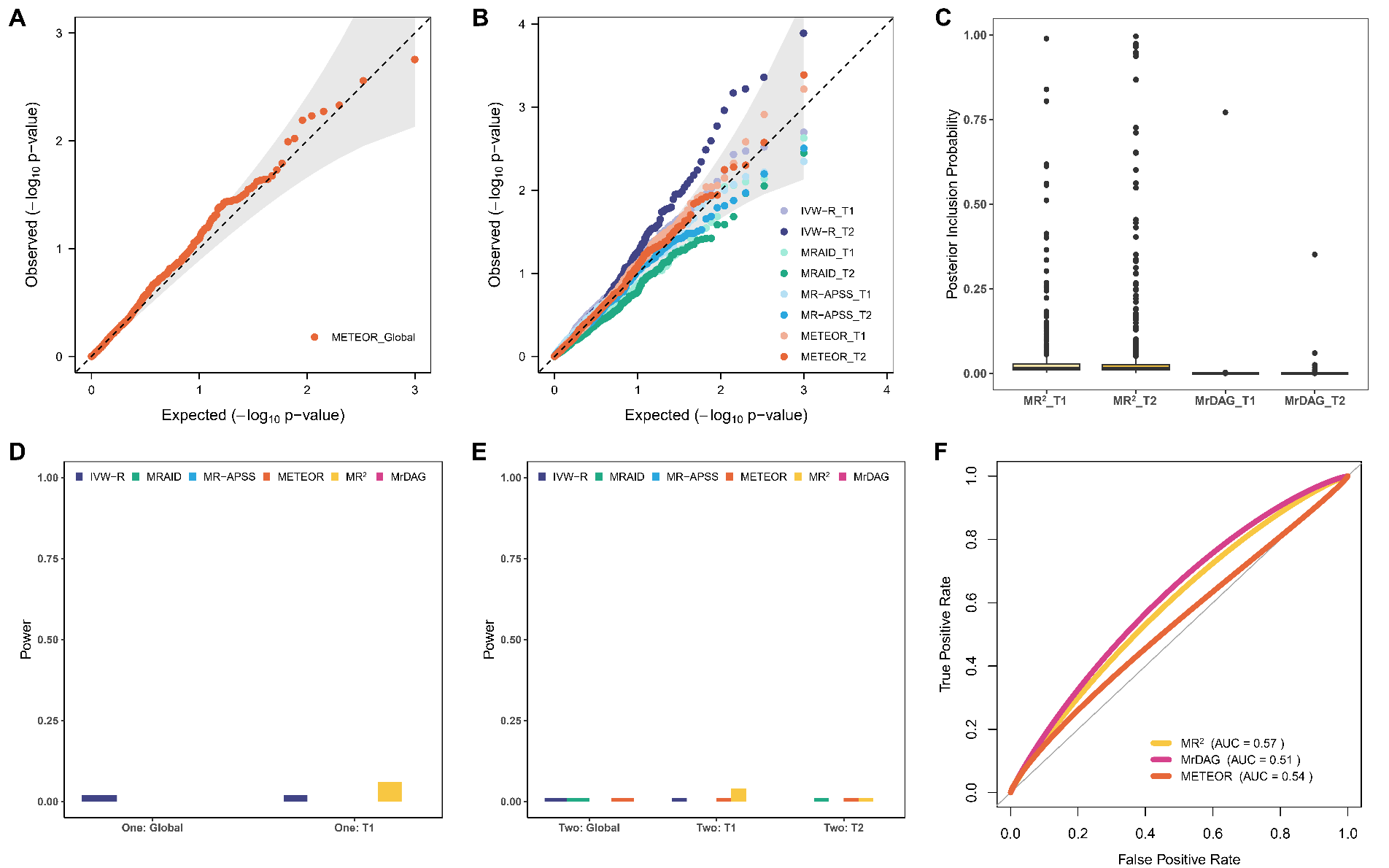


**Supplementary Figure 34** Results under the baseline scenario in which a quadratic term is included in the relationship between genetic variants and the exposure. (A) QQ plot from the global test of METEOR in the null simulations. (B) QQ plots from IVW-R, MRAID, MR-APSS and METEOR in testing the causal effects of exposure on the both outcomes (T1 and T2) in null simulations. (C) Posterior inclusion probabilities (PIPs) from MR^2^ and MrDAG for the two outcomes in the null simulations. Power performance under Bonferroni adjusted $p$-value threshold of $5\times{10}^{-4}$ for global and single tests. The results are plotted under (D) $\boldsymbol{PV}\boldsymbol{E}_{\alpha}=\left( 0.075\%,0 \right)^{T}$ and (E) $\boldsymbol{PV}\boldsymbol{E}_{\alpha}=\left( 0.075\%,0.075\% \right)^{T}$ (‘One: Global’ and ‘Two: Global’ for global tests; ‘One: T1’, ‘Two: T1’ and ‘Two: T2’ for single tests). (F) Receiver operating characteristic (ROC) curves for MR^2^, MrDAG and METEOR by plotting the TPR against the FPR.


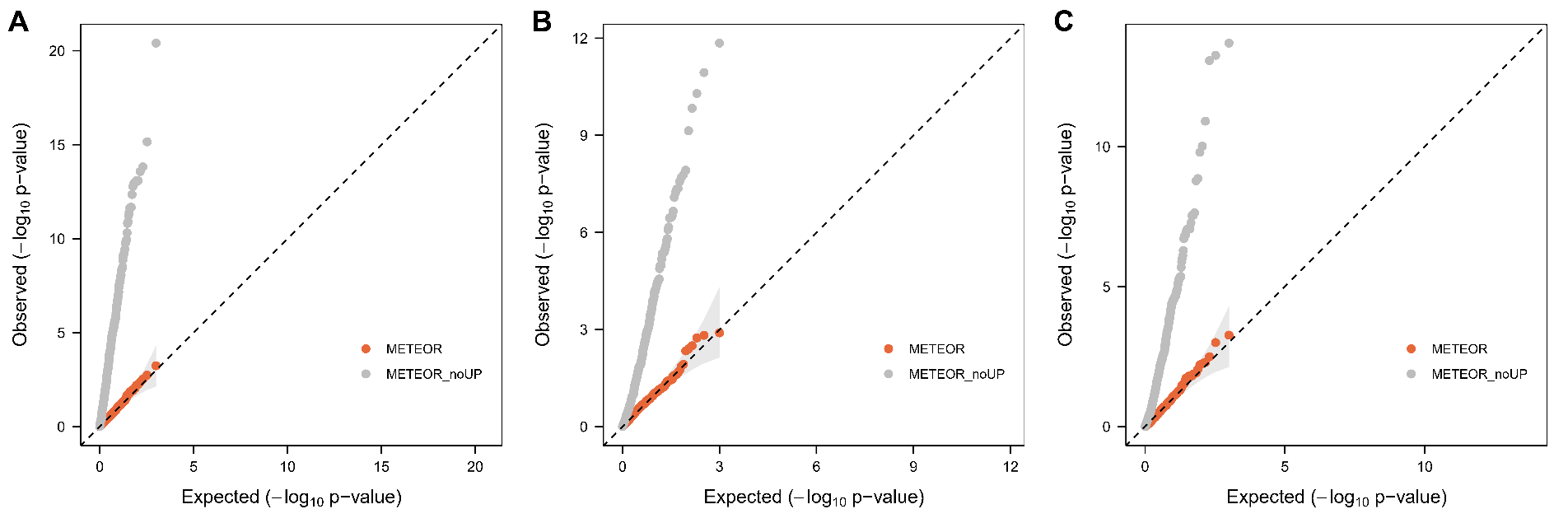


Supplementary Figure 35 Quantile-quantile (QQ) plots of METEOR in correcting horizontal pleiotropy in the baseline scenario. The scenario involves one exposure and two outcomes, with the following parameters: $PVE_{\tilde{G}_{1}}=10\%$, $K=100$, $\pi_{1k}=20\%$, $PVE_{hk}=5\%$, $n_{1}=n_{2k}=50,000 (k=1,2)$, $\tilde{\rho}_{y_{1},y_{2}}=0.5$ and $\tilde{\rho}_{x,y_{1}}=\tilde{\rho}_{x,y_{2}}=0$. Type I error control is evaluated using quantile-quantile (QQ) plots of $-\log_{10} p$ values in null simulations. (A) QQ plots from global tests of METEOR (orange) and METEOR with $\boldsymbol{\eta}_{k}$ constrained to zero (grey: METEOR_noUP). QQ plots from METEOR and METEOR_noUP in testing the causal effects of exposure on (B) the first and (C) the second outcome.


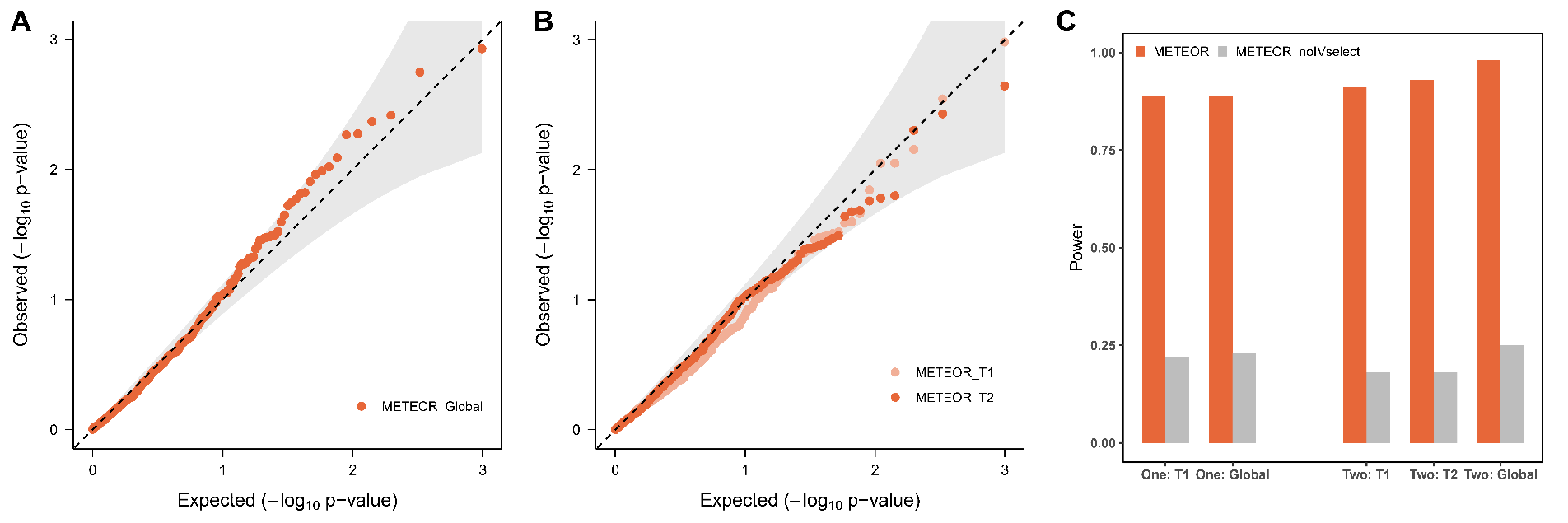


Supplementary Figure 36 Result of METEOR in self-determining the instrumental SNPs in the baseline scenario. The scenario involves one exposure and two outcomes (T1 and T2), with the following parameters: $PVE_{\tilde{G}_{1}}=10\%$, $K=100$, $\pi_{1k}=20\%$, $PVE_{hk}=5\%$ ($k=1,2$), $n_{1}=n_{2k}=50,000$, $\tilde{\rho}_{y_{1},y_{2}}=0.5$ and $\tilde{\rho}_{x,y_{1}}=\tilde{\rho}_{x,y_{2}}=0$. (A-B) QQ plots for METEOR with pre-specified independent SNPs through LD clumping as IVs in global and single tests. (C) Power performance under Bonferroni adjusted $p$-value threshold of $5\times{10}^{-4}$ for global and single tests. The results from METEOR (orange), and METEOR with pre-specified independent SNPs through LD clumping as IVs (grey, METEOR_indLD) are plotted for two alternative scenarios: $\boldsymbol{PV}\boldsymbol{E}_{\alpha}=\left( 0.075\%,0 \right)^{T}$ (left) and $\boldsymbol{PV}\boldsymbol{E}_{\alpha}=\left( 0.075\%,0.075\% \right)^{T}$ (right).


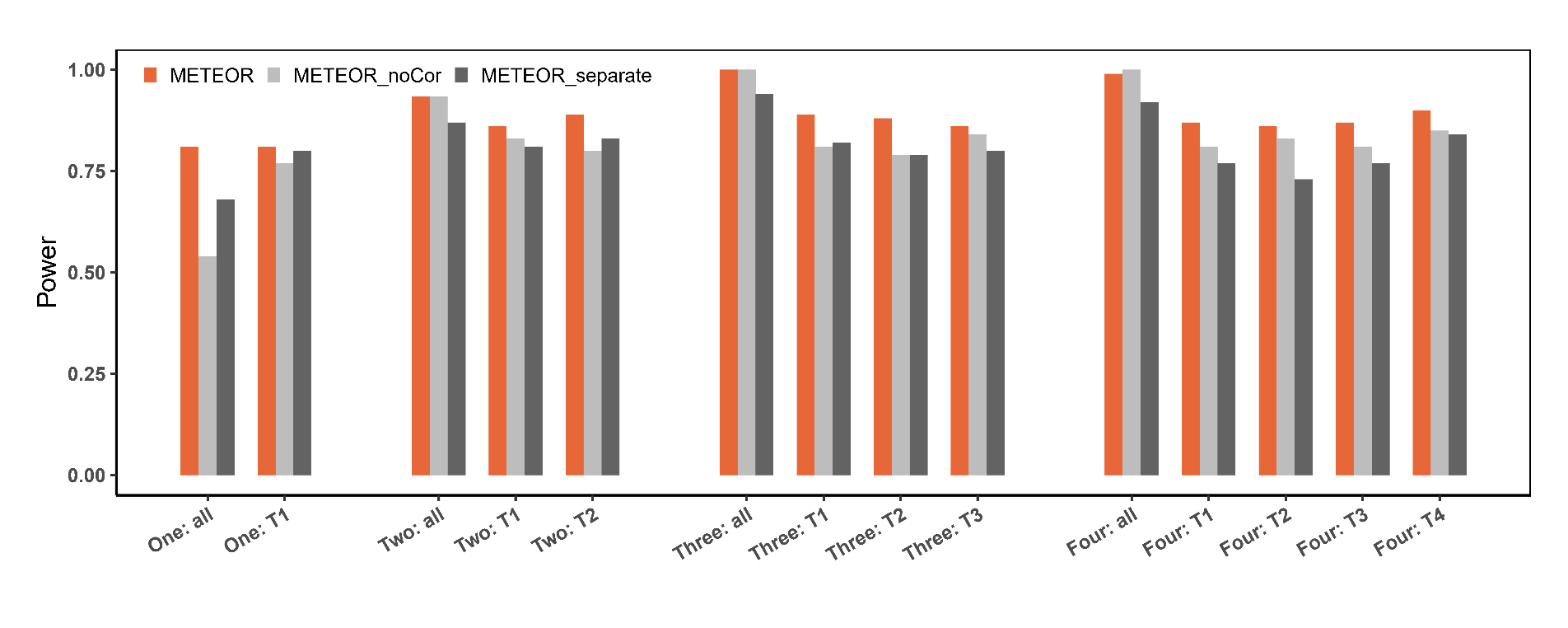


Supplementary Figure 37 Power of METEOR in correcting sample overlap. The scenario involves one exposure and four outcomes (T1, T2, T3 and T4), with the following parameters: $PVE_{\tilde{G}_{1}}=10\%$, $K=100$, $\pi_{1k}=20\%$, $PVE_{hk}=5\%$, $n_{1}=n_{2k}=50,000 (k=1,\cdots,4)$, with a correlation of 0.5 for any two outcomes and a correlation of 0 for exposure and each outcome. In alternative simulations, $PVE_{\alpha k}=0.075\%$. Power performance under Bonferroni adjusted $p$-value threshold of $5\times{10}^{-4}$ for global and single tests. The results from METEOR (orange), METEOR with $\boldsymbol{\Omega}$ constrained to be an identity matrix (light grey: METEOR_noCor), and METEOR with outcomes treated separately (dark grey: METEOR_separate) are plotted across four alternative scenarios: ranging from a setting where the exposure causally affects one trait to a setting where the exposure causally affects four traits.


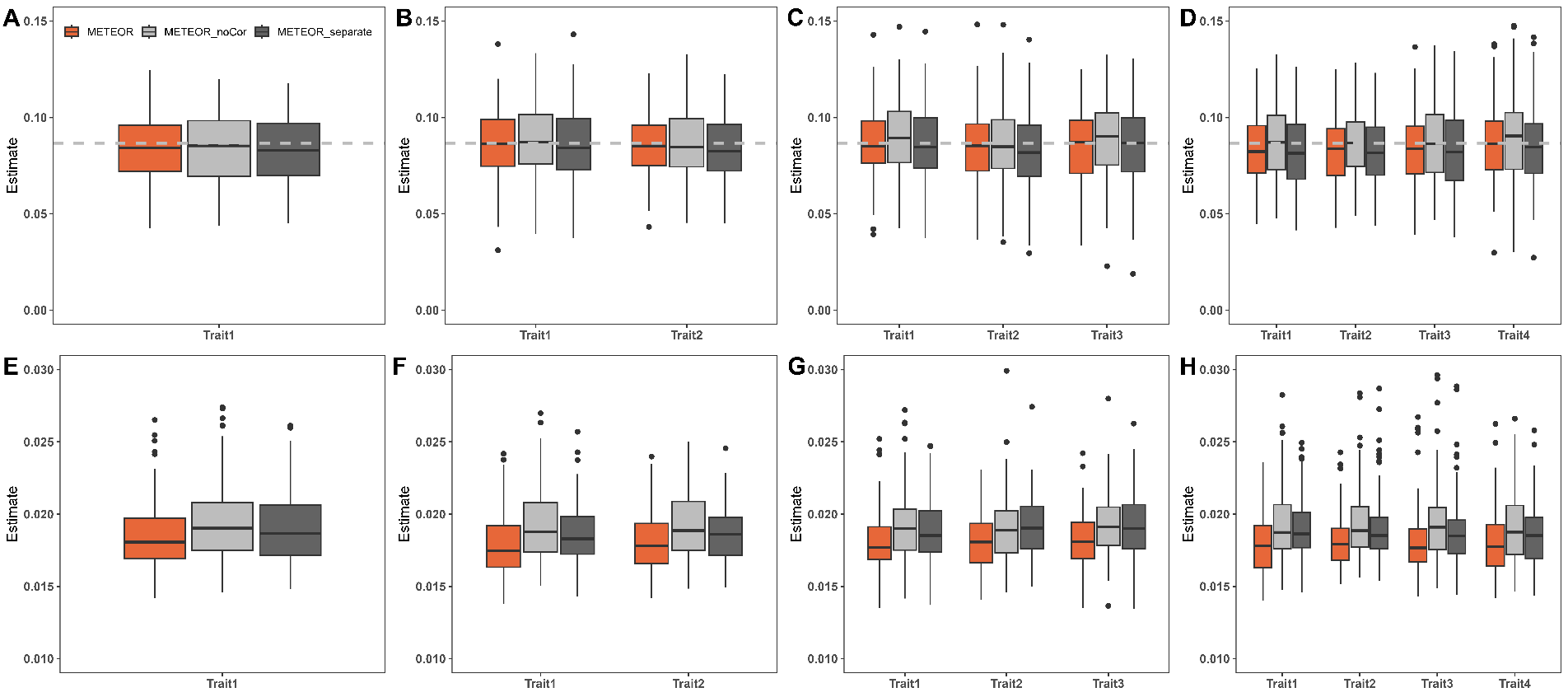


Supplementary Figure 38 Boxplots of estimate results from METEOR in correcting sample overlap. The scenario involves one exposure and four outcomes, with the following parameters: $PVE_{\tilde{G}_{1}}=10\%$, $K=100$, $\pi_{1k}=20\%$, $PVE_{hk}=5\%$, $n_{1}=n_{2k}=50,000 (k=1,\cdots,4)$, with a correlation of 0.5 for any two outcomes and a correlation of 0 for exposure and each outcome. (A-D) Estimates of causal effects and (E-H) estimates of corresponding standard deviations of estimated causal effects are displayed. The results from METEOR (orange), METEOR with $\boldsymbol{\Omega}$ constrained to be an identity matrix (light grey: METEOR_noCor), and METEOR with outcomes treated separately (dark grey: METEOR_separate) are plotted across four alternative scenarios: ranging from a setting where the exposure causally affects one trait to a setting where the exposure causally affects four traits.


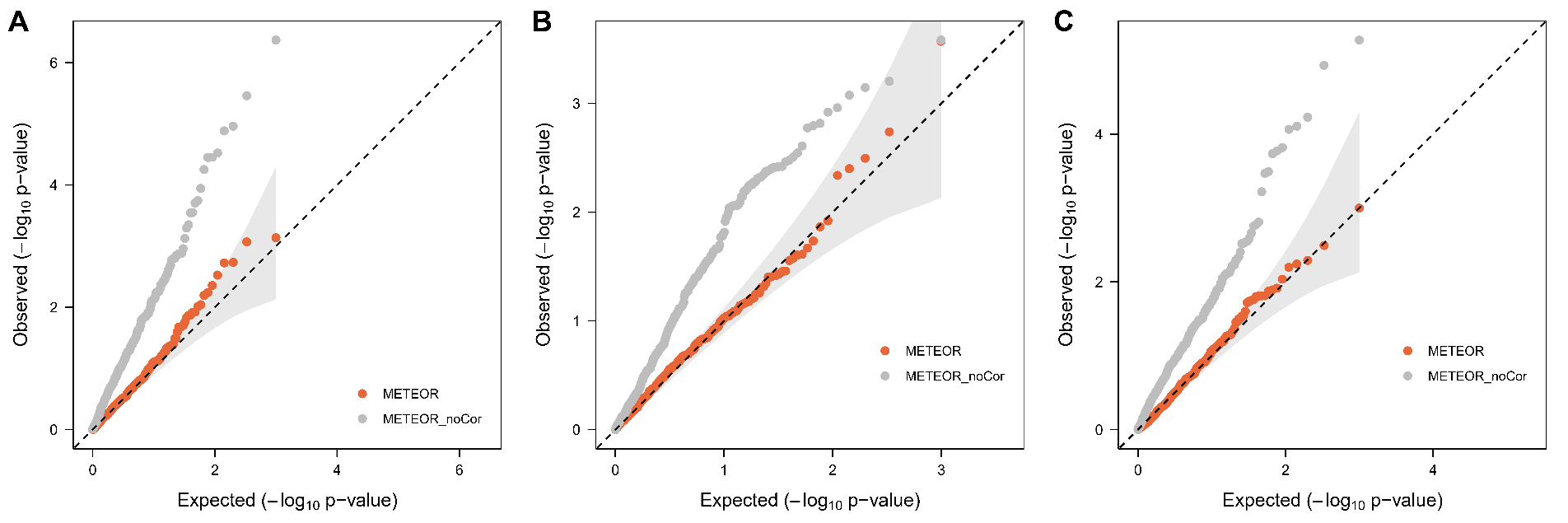


Supplementary Figure 39 Quantile-quantile (QQ) plots of METEOR in correcting sample overlap in one sample MR. The scenario involves one exposure and two outcomes, with the following parameters: $PVE_{\tilde{G}_{1}}=10\%$, $K=100$, $\pi_{1k}=20\%$, $PVE_{hk}=5\%$, $n_{1}=n_{2k}=50,000 (k=1,2)$ and $\tilde{\rho}_{u,v}=0.5$ for $u, v\in(x, y_{1},y_{2})$. Type I error control is evaluated by quantile-quantile (QQ) plots of $-\log_{10} p$ values in null simulations. (A) QQ plots from global tests of METEOR (orange) and METEOR with $\boldsymbol{\Omega}$ constrained to be an identity matrix (grey: METEOR_noCor) testing. QQ plots from METEOR and METEOR_noCor in testing the causal effects of exposure on (B) the first and (C) the second outcomes.


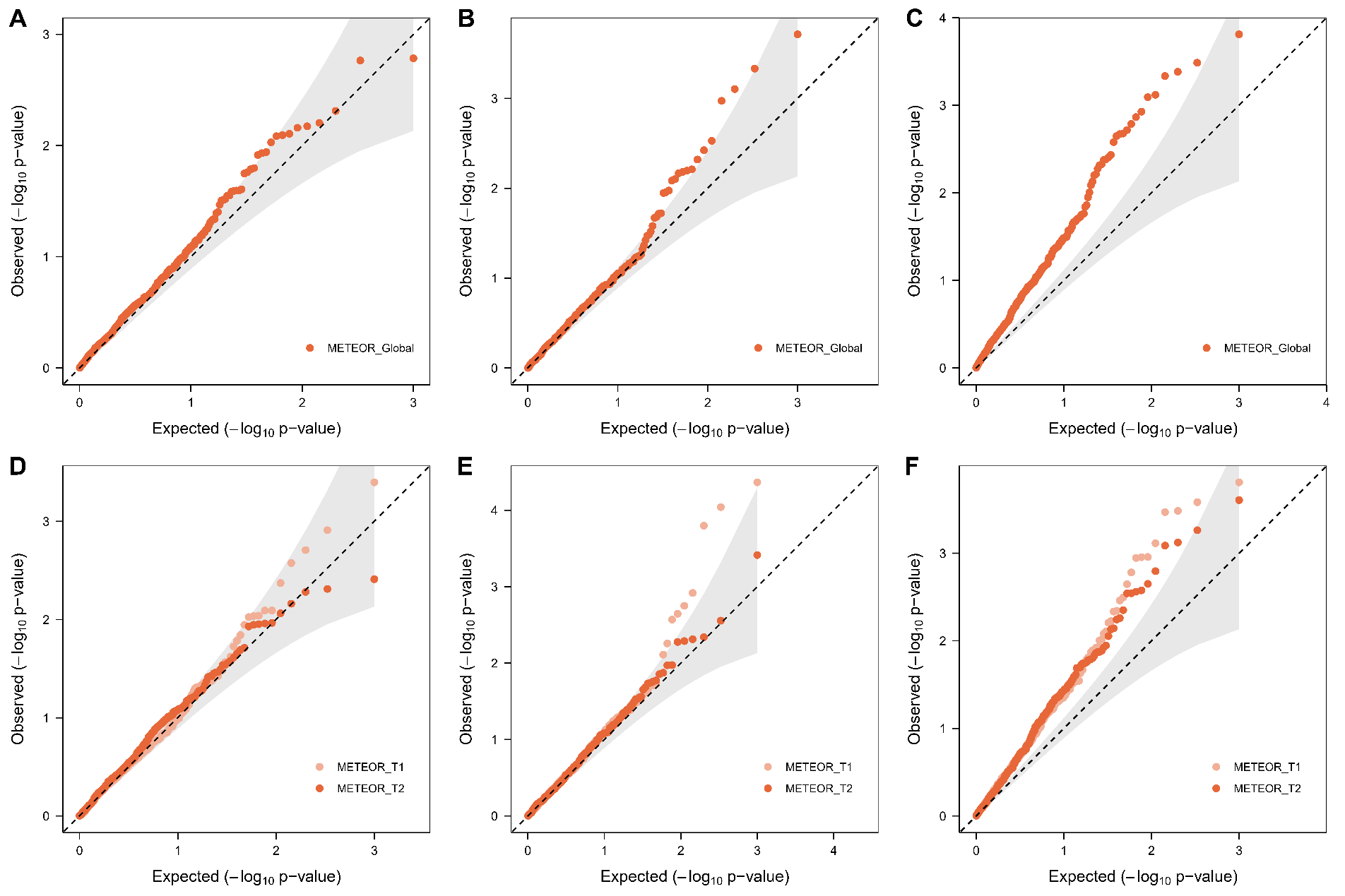


Supplementary Figure 40 Results from simulations with correlated and uncorrelated horizontal pleiotropy. Sample sizes for both exposure and outcomes are 50,000. The scenario involves one exposure and two outcomes (T1 and T2), with the following parameters: $PVE_{\tilde{G}_{1}}=10\%$, $K=100$, $\pi_{1k}=0.2$, $PVE_{hk}=5\%$ ($k=1,2$), $\tilde{\rho}_{y_{1},y_{2}}=0.5$ and $\tilde{\rho}_{x,y_{1}}=\tilde{\rho}_{x,y_{2}}=0$. Three proportions of selected SNPs with correlated horizontal pleiotropy are considered, including $\pi_{ck}=0.02$,$0.05$ and $0.1$, and the effect of confounder on $k$-th outcome is $\omega=\sqrt{0.05}$. Type I error control, evaluated using quantile-quantile (QQ) plots of $-\log_{10} p$ values in null simulations. (A-C) QQ plot from global tests of METEOR. (D-F) QQ plots from METEOR in testing the causal effects of exposure on both outcomes.

# Supplementary Tables

## Supplementary Table 1 Summary of multi-outcome MR methods

| Method | MR^2^ | MrDAG | MR-AHC | MRMO | BMRMO | METEOR |
| --- | --- | --- | --- | --- | --- | --- |
| Input | summary | summary | summary | individual | individual | summary |
| SNPs | independent | independent | independent | weakly corr./indep. | moderately corr./indep. | correlated |
| E-O  overlap | ✗ | ✓ | ✗ | ✗ | ✗ | ✓ |
| O-O  overlap | ✓ | ✓ | ✓ | 100% | ✓ | ✓ |
| HP | global/ SNP-spec. | globa/ SNP-spec. | ✗ | ✗ | ✗ | outcome- /SNP-spec. |
| Output | PIP | PIP | $p$-value* | $p$-value | credible interval/  $p$-value/  bayes factor | $p$-value |
| Global test | ✗ | ✗ | ✗ | ✓ | ✓ | ✓ |
| Single test | ✓ | ✓ | ✗ | ✓ | ✓ | ✓ |
| Code availability | ✓ | ✓ | ✗* | ✓ | ✗ | ✓ |

Multi-outcome MR methods are categorized based on the input data type (individual-level or summary-level), characteristic of selected SNPs (independent or correlated), modeling of sample overlap (between exposure and outcome, among outcomes), modeling of horizontal pleiotropy (global, SNP-specific or outcome-specific), output (posterior inclusion probability [PIP], $p$-value, or others), global test to examine whether the exposure affects at least one outcome, single test to evaluate whether the exposure affects a specific outcome, and code availability. ‘✗’ indicates the method does not take the corresponding item into account. For example, MR^2^ is unable to account for the sample overlap between exposure and outcome. ‘100%’ indicates that the method is applicable only when the outcomes are derived from the same dataset. ‘global pleiotropy’ refers to pleiotropic effects shared across at least two outcomes, which are typically estimated using all SNPs, without differentiation according to outcomes and SNPs. ‘SNP-specific pleiotropy’ indicates the pleiotropic effects that differ across SNPs, while ‘outcome-specific pleiotropy’ denotes the pleiotropic effects that differ across outcomes. ‘$p$-value*’ represents that MR-AHC is a cluster-based MR method, which first identifies variant clusters, subsequently carries causal inference for each cluster, and then gets multiple causal effects and corresponding $p$-values. ‘✗*’ indicates that the available code for MR-AHC is restricted to analysis with at most two outcomes. E-O overlap: the sample overlap exists between exposure and outcome; O-O overlap: the sample overlap exists among outcomes; HP: horizontal pleiotropy.

## Supplementary Table 2 The parameter settings in the simulations

| Parameter | Setting |
| --- | --- |
| Proportion of variance in the exposure explained by instrumental SNPs ($PVE_{\tilde{G}_{x}}$) | 10%, 15% |
| Proportion of variance in the $k$-th outcome explained by the causal effect term $PVE_{\alpha k}$ | 0.05%, 0.075%, 0.1%, 0.15%, 0.2%, 0.25% |
| Proportion of phenotypic variance in the $k$-th outcome explained by horizontal pleiotropy ($PVE_{hk}$) | 5%, 10% |
| Proportion of instrumental SNPs with horizontal pleiotropy for selected SNPs ($\pi_{1k}$) | 0.1, 0.2, 0.3, 0.4 |
| Sample sizes for exposure and $k$-th outcome ($n_{1}$, $n_{2k}$) | 20,000, 50,000, 10,000 |
| Number of outcomes | 2, 4 |
| Correlation between different traits | From 0 to 0.9 |
| Proportion of sample overlap | 0%, 100% |
| Various priors for $\pi_{\beta}$ distributions | Beta (0.5, 49.5), Beta (0.5, 4.5), Beta (0.5, 2.0) |
| Various priors for $\pi_{1k}$ distributions | Beta (0.5, 2.0), Beta (0.5, 1.5) and Beta (0.5, 1.0) |
| Various priors for $\pi_{0k}$ distributions | Beta (0.05, 49.95), Beta (0.05, 9.95) and Beta (0.05, 4.95) |

## Supplementary Table 3 Individual data used in this study

| Trait  (Abbreviation) | Database | Field ID/Link |
| --- | --- | --- |
| Total cholesterol (TC) | UK Biobank | 30690 |
| High density cholesterol (HDL) |  | 30760 |
| Low density cholesterol (LDL) |  | 30780 |
| Triglycerides (TG) |  | 30870 |
| Skin color (SC, the color of skin without tanning) |  | 1717 |
| Hair color (HC, nature, before greying) |  | 1747 |

## Supplementary Table 4 Summary data used in this study

| Trait  (Abbreviation) | Field ID/Link | Number  (case/control) |
| --- | --- | --- |
| Body mass index (BMI) | http://www.nealelab.is/uk-biobank | / |
| Type 2 diabetes (T2D) | http://www.nealelab.is/uk-biobank |  |
| Systolic blood pressure (SBP) | http://www.nealelab.is/uk-biobank |  |
| Diastolic blood pressure (DBP) | http://www.nealelab.is/uk-biobank |  |
| High density cholesterol (HDL) | http://www.nealelab.is/uk-biobank |  |
| Low density cholesterol (LDL) | http://www.nealelab.is/uk-biobank |  |
| Triglycerides (TG) | http://www.nealelab.is/uk-biobank |  |
| Atrial fibrillation (AF) | https://www.finngen.fi/en | 63,532/252,810 |
| Angina pectoris (AP) | https://www.finngen.fi/en | 44,588/416,171 |
| Coronary heart disease (CHD) | https://www.finngen.fi/en | 56,650/443,698 |
| Heart failure (HF) | https://www.finngen.fi/en | 37,653/462,695 |
| Myocardial infarction (MI) | https://www.finngen.fi/en | 31,666/416,171 |
| Depression (DP) | https://www.finngen.fi/en | 59,333/434,831 |
| Anxiety disorders (AN) | https://www.finngen.fi/en | 56,552/362,304 |
| Inflammatory bowel disease (IBD) | https://www.finngen.fi/en | 10960/489388 |
| Irritable bowel syndrome (IBS) | https://www.finngen.fi/en | 13268/394725 |
| Gastroduodenal ulcer (GDU) | https://www.finngen.fi/en | 12074/423785 |
| Gastroesophageal reflux disease (GERD) | https://www.finngen.fi/en | 36025/423785 |

## Supplementary Table 5 CPU time (seconds) for different methods

| $K$ | #SNPs | IVW-R | MRAID | MR-APSS | METEOR | MR^2^ | MrDAG |
| --- | --- | --- | --- | --- | --- | --- | --- |
| 2 | 50 | 0.009 | 0.308 | 7.394 | 0.344 | 79.037 | 54.224 |
|  | 100 | 0.009 | 0.896 | 24.954 | 0.832 | 82.684 | 56.853 |
|  | 500 | 0.010 | 18.520 | 58.788 | 11.539 | 107.493 | 72.519 |
| 4 | 50 | 0.012 | 0.561 | 8.434 | 0.824 | 141.561 | 88.433 |
|  | 100 | 0.013 | 1.625 | 25.585 | 2.139 | 147.431 | 99.586 |
|  | 500 | 0.013 | 33.382 | 71.685 | 32.527 | 189.504 | 136.713 |

Computation was carried out on a single thread of an Intel Xeon Gold E5-2697 v3 CPU. The computation time was averaged across 500 replicates. $K$ is the number of outcomes in analysis. #SNP denotes the number of instrumental variables included in the model. The computation times for all methods are based on the baseline scenario with two or four outcomes in null simulations.

## Supplementary Table 6 Six MR methods and the corresponding software

| Method | R package | Version | Function | Tuning parameters |
| --- | --- | --- | --- | --- |
| IVW-R | MendelianRandomization | 0.6.0 | mr_ivw | Default settings |
| MRAID | MRAID | 1.0 | MRAID | Default settings |
| MR-APSS | MRAPSS | 0.2.0 | MRAPSS | Default settings |
| METEOR | METEOR | 1.0 | METEOR | Default settings |
| MR^2^ | MR2 | 0.1.1 | MR2 | (beta_Y, beta_X, EVgamma = 0.5, niter = 7500, burnin = 2500, thin = 5, monitor = 500, seed = 28061971+i) |
| MrDAG | MrDAG | 0.1.1 | MrDAG | Real datat: (data =dat,niter = 110000, burnin = 10000, thin = 100, tempMax = 10, pp = 0.05, MrDAGcheck = MrDAGcheck, fileName = NULL)  Simulation: (data =dat,niter = 75000, burnin = 25000, thin = 10, tempMax = 10, pp = 0.05, MrDAGcheck = MrDAGcheck, fileName = NULL) |

## Supplementary Table 7 Simulation scenarios in this study

| **Scenario** | **Figure** |
| --- | --- |
| Baseline setting: in null simulation | Fig. 2A-C |
| Baseline setting: in alternative simulation | Fig. 2G-H, Fig. 16F |
| Estimates of the causal effects | Fig. 3, Supplementary Figs. 13, 25-26 |
| Different sample sizes | Fig. 2, Supplementary Figs. 2, 22 |
| Different numbers of outcomes | Fig. 2, Supplementary Fig. 3 |
| Different proportions of instrumental SNPs having horizontal pleiotropy on $k$-th outcome for selected SNPs ($\pi_{1k}$) | Supplementary Figs. 4, 16 |
| Different variances of the exposure explained by instrumental SNPs ($PVE_{\tilde{G}_{1}}$) | Supplementary Figs. 5, 17 |
| Different proportions of phenotypic variance in the $k$-th outcome explained by horizontal pleiotropy ($PVE_{hk}$) | Supplementary Figs. 6. 18 |
| Different correlations among outcomes | Supplementary Figs.7-8, 23 |
| Scenario without horizontal pleiotropy | Supplementary Figs. 9, 14 |
| Different priors for $\pi_{\beta}$ distribution | Supplementary Fig. 10 |
| Different priors for $\pi_{1k}$ distribution | Supplementary Fig. 11 |
| Different priors for $\pi_{0k}$ distribution | Supplementary Fig. 12 |
| Scenario in one sample MR setting | Fig. 2D-F and Supplementary Figs. 14-15 |
| Different variance in the $k$-th outcome explained by the causal term ($PVE_{\alpha k}$) | Supplementary Figs. 19-20 |
| Opposite causal effects for both outcomes ($\boldsymbol{\alpha}^{\boldsymbol{*}}$) | Supplementary Fig. 21 |
| Evaluating the power based on an unified FDR of 0.05 | Supplementary Fig. 24 |
| Different combinations of sample overlap and correlation | Supplementary Figs. 27-30 |
| Assess the robustness against the non-normality | Supplementary Figs. 31-33 |
| Investigate the impact of nonlinear genetic effects | Supplementary Fig. 34 |
| Test the influence of $\boldsymbol{\eta}_{k}$ | Supplementary Fig. 35 |
| Test the influence of correlated SNPs | Supplementary Fig. 36 |
| Test the influence of correlations among exposure and outcomes | Supplementary Figs. 37-39 |
| Scenarios with correlated and uncorrelated horizontal pleiotropy | Supplementary Fig. 40 |

## Supplementary Table 8 Estimates of causal effect and 95% confidence intervals from different MR methods in the positive control analysis

| Sample overlap (%) | Trait | IVW-R | MRAID | MR-APSS | METEOR | MR^2^ | MrDAG |
| --- | --- | --- | --- | --- | --- | --- | --- |
| 0 | TC | **0.944**  **(0.919, 0.969)** | 0.997  (0.961, 1.033) | 0.952  (0.861,1.043) | 1.015  (0.963, 1.068) | **0.001**  **(0,0.016)** | **0.944**  **(0.921,0.968)** |
|  | HDL | **0.934**  **(0.912, 0.957)** | 0.984  (0.957, 1.011) | 0.953  (0.877,1.028) | 0.992  (0.953, 1.032) | **0.047**  **(0,0.058)** | **0.936**  **(0.913,0.958)** |
|  | LDL | **0.962**  **(0.937, 0.988)** | 1.032  (0.830, 1.234) | 0.964  (0.865,1.063) | 1.051  (0.956, 1.145) | **0**  **(0,0)** | **0.962**  **(0.939,0.987)** |
|  | TG | **0.950**  **(0.926, 0.973)** | 0.979  (0.935, 1.023) | 0.941  (0.857,1.024) | 1.006  (0.944, 1.068) | **0.057**  **(0.056,0.058)** | **0.950**  **(0.926,0.975)** |
| 100 | TC | 1 (0.985,1.015) | 1.043 (1.002,1.083) | 1.001 (0.922,1.081) | 0.948 (0.746,1.150) | **0.003 (0,0.02)** | 0.9999992  (0.9998,1.0002) |
|  | HDL | 1 (0.987,1.013) | 0.992 (0.930,1.055) | 1.003 (0.938,1.068) | 1.023 (0.992,1.053) | **0.039 (0,0.045)** | 0.9999985 (0.9999,1.0001) |
|  | LDL | 1 (0.985,1.015) | 1.141 (1.039,1.243) | 1.001 (0.917,1.085) | 0.994 (0.861,1.127) | **0 (0,0)** | 1.0000026 (0.9998,1.0002) |
|  | TG | 1 (0.984,1.016) | 1.001 (0.974,1.029) | 1.002 (0.926,1.078) | 0.974 (0.932,1.015) | **0.508 (0.508,0.508)** | 1.00000163 (0.9998,1.0002) |

Values are bold if the interval does not cover the true causal effect.

## Supplementary Table 9 $\boldsymbol{p}$-values or PIP of positive control analysis for all MR methods in two sample MR setting

| Exposure | Outcome | IVW-R | | MRAID | | MR-APSS | | METEOR | | MR^2^ | MrDAG |
| --- | --- | --- | --- | --- | --- | --- | --- | --- | --- | --- | --- |
|  |  | P  (single) | P  (Global) | P  (single) | P  (Global) | P  (single) | P  (Global) | P  (single) | P  (Global) | PIP | PIP |
| TC | TC | 0 | 0 | 0 | 0 | 3.46e-93 | 1.38e-92 | 6.06e-311 | 0 | 0.064 | 1 |
|  | HDL | 0.010 |  | 0.035 |  | 4.50e-07 |  | 0.362 |  | 0.028 | 0 |
|  | LDL | 2.62e-235 |  | 0 |  | 2.87e-43 |  | 8.65e-185 |  | 0.014 | 0 |
|  | TG | 5.00e-4 |  | 0.734 |  | 0.005 |  | 8.63e-07 |  | 0.026 | 0 |
| HDL | TC | 9.47e-07 | 0 | 0.029 | 0 | 1.83e-12 | 8.24e-134 | 4.86e-07 | 0 | 0.012 | 0.019 |
|  | HDL | 0 |  | 0 |  | 2.06e-134 |  | 0 |  | 0.944 | 1 |
|  | LDL | 0.322 |  | 0.014 |  | 0.722 |  | 3.06e-46 |  | 0.006 | 0 |
|  | TG | 1.58e-16 |  | 3.21e-07 |  | 1.19e-19 |  | 2.16e-04 |  | 0.028 | 0 |
| LDL | TC | 0 | 0 | 0 | 0 | 3.97e-42 | 3.26e-80 | 3.36e-16 | 0 | 0.005 | 0 |
|  | HDL | 0.061 |  | 1.05e-4 |  | 0.846 |  | 0.0142 |  | 0.05 | 0 |
|  | LDL | 0 |  | 0 |  | 8.15e-81 |  | 8.56e-105 |  | 0.013 | 1 |
|  | TG | 0.009 |  | 0.056 |  | 0.001 |  | 0.434 |  | 0.204 | 0 |
| TG | TC | 1.75e-06 | 0 | 0.625 | 0 | 0.006 | 8.12e-108 | 0.647 | 0 | 0.013 | 0 |
|  | HDL | 3.96e-15 |  | 0 |  | 7.57e-21 |  | 5.10e-29 |  | 0.021 | 0 |
|  | LDL | 1.24e-07 |  | 0.081 |  | 5.62e-06 |  | 0.395 |  | 0.007 | 0.217 |
|  | TG | 0 |  | 0 |  | 2.03e-108 |  | 5.82e-225 |  | 0.999 | 1 |

Bonferroni adjusted $p$-value threshold is $3.125\times{10}^{-3}$ and $1.25\times{10}^{-2}$ for single and global test, respectively. Posterior inclusion probability: PIP.

## Supplementary Table 10 $\boldsymbol{p}$-values or PIP of positive control analysis for all MR methods in one sample MR setting

| Exposure | Outcome | IVW-R | MRAID | MR-APSS | METEOR | MR^2^ | MrDAG |
| --- | --- | --- | --- | --- | --- | --- | --- |
|  |  | P | P | P | P | PIP | PIP |
| TC | TC | 0 | 0 | 3.13e-134 | 3.27e-20 | 0.159 | 1 |
| HDL | HDL | 0 | 0 | 1.81e-200 | 0 | 0.964 | 1 |
| LDL | LDL | 0 | 0 | 5.46e-121 | 1.53e-48 | 0.022 | 1 |
| TG | TG | 0 | 0 | 3.35e-147 | 0 | 1 | 1 |

Bonferroni adjusted $p$-value threshold is $3.125\times{10}^{-3}$ for single test. Posterior inclusion probability: PIP.

## Supplementary Table 11 $\boldsymbol{p}$-values or PIP of negative control analysis for all MR methods

| Sample overlap (%) | Exposure | Outcome | IVW-R | | MRAID | | MR-APSS | | METEOR | | MR^2^ | MrDAG |
| --- | --- | --- | --- | --- | --- | --- | --- | --- | --- | --- | --- | --- |
|  |  |  | P  (single) | P  (global) | P  (single) | P  (global) | P  (single) | P  (global) | P  (single) | P  (global) | PIP | PIP |
| 0 | TC | SC | 0.560 | 0.894 | 0.099 | 0.198 | 0.183 | 0.366 | 0.849 | 0.221 | 0.015 | 0 |
|  |  | HC | 0.447 |  | 0.212 |  | 0.24 |  | 0.086 |  | 0.007 | 0 |
|  | HDL | SC | 0.975 | 1 | 0.162 | 0.324 | 0.444 | 0.888 | 0.732 | 0.935 | 0.008 | 0 |
|  |  | HC | 0.526 |  | 0.585 |  | 0.537 |  | 0.895 |  | 0.005 | 0 |
|  | LDL | SC | 0.872 | 0.470 | 0.009 | 0.018 | 0.824 | 0.872 | 0.386 | 0.054 | 0.013 | 0 |
|  |  | HC | 0.235 |  | 0.115 |  | 0.436 |  | 0.024 |  | 0.011 | 0 |
|  | TG | SC | **1.73e-04** | **3.46e-04** | 0.709 | 1 | 0.754 | 1 | 0.089 | 0.232 | **0.962** | 0.001 |
|  |  | HC | 0.59 |  | 0.517 |  | 0.519 |  | 0.989 |  | 0.006 | 0 |
| 100 | TC | SC | 0.215 | 0.430 | 0.142 | 0.284 | 0.327 | 0.654 | 0.639 | 0.036 | 0.023 | 0 |
|  |  | HC | 0.278 |  | 0.276 |  | 0.947 |  | 0.011 |  | 0.013 | 0 |
|  | HDL | SC | 0.460 | 0.920 | 0.502 | 0.946 | 0.304 | 0.608 | 0.236 | 0.426 | 0.01 | 0 |
|  |  | HC | 0.743 |  | 0.473 |  | 0.971 |  | 0.626 |  | 0.007 | 0 |
|  | LDL | SC | 0.433 | 0.278 | **1.16e-05** | **2.32e-05** | 0.734 | 0.928 | 0.239 | 0.033 | 0.021 | 0 |
|  |  | HC | 0.139 |  | 0.089 |  | 0.464 |  | 0.016 |  | 0.017 | 0 |
|  | TG | SC | **5.96e-06** | **1.19e-05** | 0.026 | 0.052 | 0.706 | 0.248 | 0.013 | 0.041 | **0.999** | 0.021 |
|  |  | HC | 0.028 |  | 0.507 |  | 0.124 |  | 0.780 |  | 0.073 | 0 |

Bonferroni adjusted $p$-value threshold is $6.25\times{10}^{-3}$ and $1.25\times{10}^{-2}$ for single and global test, respectively. Values are bold if $p$-values less than Bonferroni adjusted $p$-values or PIPs larger than 0.5. Posterior inclusion probability: PIP.

## Supplementary Table 12 Results of shared exposure detection analysis based on brain-heart axis

| Exposure | Outcome | IVW-R |  |  | MRAID |  |  | MR-APSS | | |  | | METEOR | |  | |  | | MR^2^ | | MrDAG | | |  |  |
| --- | --- | --- | --- | --- | --- | --- | --- | --- | --- | --- | --- | --- | --- | --- | --- | --- | --- | --- | --- | --- | --- | --- | --- | --- | --- |
|  |  | Effect | P (single) | P (global) | Effect | P (single) | P (global) | Effect | P (single) | P (global) | | Effect | | P (single) | | P (global) | | Effect | | PIP | | Effect | PIP | |  |
| BMI | AF | 0.463 | **8.92e-42** | **3.37e-62** | 0.057 | **3.00e-07** | **0** | 0.174 | **4.69e-26** | **4.73e-30** | | 0.128 | | **6.86e-88** | | **7.44e-210** | | 0.105 | | **1** | | 0.011 | 0.038 | | |
|  | AP | 0.297 | **1.00e-24** |  | 0.059 | **0** |  | 0.063 | **3.37e-06** |  |  | 0.094 | | **1.44e-98** | |  |  | 0.036 | | 0.47 | | 0 | 0 | | |
|  | CHD | 0.277 | **7.96e-26** |  | 0.023 | **1.06e-03** |  | 0.069 | **9.81e-07** |  |  | 0.089 | | **2.38e-91** | |  |  | 0.018 | | 0.443 | | 0 | 0 | | |
|  | HF | 0.446 | **4.81e-63** |  | 0.057 | **1.40e-08** |  | 0.123 | **6.75e-31** |  |  | 0.115 | | **1.1e-137** | |  |  | 0.184 | | **1** | | 0.44 | **1** | | |
|  | MI | 0.346 | **8.48e-27** |  | 0.016 | 0.039016 |  | 0.07 | **1.22e-07** |  |  | 0.090 | | **1.77e-88** | |  |  | 0.033 | | 0.466 | | 0.016 | 0.061 | | |
|  | DP | 0.101 | **6.02e-05** |  | -0.035 | **4.60e-06** |  | 0.014 | 0.221 |  |  | 0.003 | | 0.481 | |  |  | 0.078 | | **0.958** | | 0.011 | 0.125 | | |
|  | AN | 0.024 | 0.383 |  | -0.038 | **6.66e-07** |  | -0.004 | 0.775 |  |  | 0.000 | | 0.950 | |  |  | -0.004 | | 0.064 | | 0 | 0 | | |
| T2D | AF | 0.088 | 0.581 | 0.154 | 0.094 | 0.372 | 0.238 | 0.858 | 0.151 |  | | 0.083 | | 0.276 | | 0.192 | | 0 | | **0.677** | | 0.015 | 0.027 | | |
|  | AP | 0.339 | 0.022 |  | 0.149 | 0.333 |  | 0.19 | 0.675 |  | | 0.163 | | 0.011 | |  |  | 0.184 | | **0.646** | | -8.75e-04 | 0.032 | | |
|  | CHD | 0.307 | 0.099 |  | 0.012 | 0.927 |  | 0.035 | 0.931 |  | | 0.143 | | 0.016 | |  |  | 0.12 | | **0.673** | | -7.35e-03 | 0.023 | | |
|  | HF | 0.171 | 0.141 |  | 0.123 | 0.034 |  | 0.173 | 0.508 | 1 | | 0.125 | | 0.027 | |  |  | 0.008 | | **0.669** | | 5.11e-03 | 0.052 | | |
|  | MI | 0.312 | 0.183 |  | -0.099 | 0.192 |  | -0.264 | 0.509 |  | | 0.142 | | 0.027 | |  |  | -0.067 | | **0.664** | | -0.019 | 0.01 | | |
|  | DP | 0.078 | 0.294 |  | 0.047 | 0.401 |  | 0.259 | 0.459 |  | | 0.051 | | 0.389 | |  |  | 0.192 | | **0.671** | | -5.09e-04 | 0.051 | | |
|  | AN | -0.071 | 0.452 |  | 0.018 | 0.775 |  | 0.011 | 0.959 |  | | 0.015 | | 0.812 | |  |  | 0.009 | | **0.67** | | -0.013 | 0.025 | | |
| SBP | AF | 0.876 | **6.25e-38** | **4.37e-37** | 0.109 | **7.20e-09** | **1.55e-15** | 0.324 | **1.50e-30** | **1.05e-29** | | 0.212 | | **6.23e-36** | | **1.82e-121** | | 0.501 | | **1** | | 0.815 | **1** | | |
|  | AP | 0.615 | **1.28e-25** |  | 0.025 | 0.062 |  | 0.195 | **1.75e-22** |  |  | 0.136 | | **2.1e-103** | |  |  | 0.069 | | **0.998** | | 0.193 | 0.401 | | |
|  | CHD | 0.516 | **2.54e-18** |  | 0.081 | **2.22e-16** |  | 0.174 | **2.49e-16** |  |  | 0.118 | | **1.77e-70** | |  |  | 0 | | 0.02 | | 0 | 0 | | |
|  | HF | 0.367 | **7.56e-17** |  | -0.006 | 0.671 |  | 0.102 | **2.91e-15** |  |  | 0.090 | | **5.17e-23** | |  |  | 0.33 | | **1** | | 0 | 0 | | |
|  | MI | 0.622 | **1.59e-19** |  | 0.028 | 0.111 |  | 0.162 | **1.23e-16** |  |  | 0.107 | | **5.44e-57** | |  |  | 0.007 | | 0.133 | | 0 | 0 | | |
|  | DP | 0.028 | 0.429 |  | -0.002 | 0.898 |  | 0.005 | 0.671 |  |  | 0.021 | | 0.015 | |  |  | 0 | | 0.025 | | 0 | 0 | | |
|  | AN | -0.009 | 0.817 |  | -0.018 | 0.087 |  | 0.009 | 0.474 |  |  | 0.042 | | **1.83e-06** | |  |  | -0.002 | | 0.05 | | 0 | 0 | | |
| DBP | AF | 0.791 | **1.22e-33** | **8.51e-33** | -0.091 | 0.019 | **9.09e-12** | 0.265 | **9.07e-15** | **6.35e-14** | | 0.194 | | **1.25e-48** | | **3.44e-161** | | 0.341 | | **1** | | 0.789 | **1** | | |
|  | AP | 0.398 | **8.95e-10** |  | 0.051 | **4.74e-05** |  | 0.124 | **6.73e-08** |  |  | 0.128 | | **5.3e-102** | |  |  | 0 | | 0.011 | | 0 | 0 | | |
|  | CHD | 0.393 | **5.52e-11** |  | 0.076 | **1.30e-12** |  | 0.134 | **2.69e-08** |  |  | 0.124 | | **6.75e-95** | |  |  | 0 | | 0.019 | | 0 | 0 | | |
|  | HF | 0.283 | **5.86e-11** |  | 0.008 | 0.421 |  | 0.072 | **1.94e-06** |  |  | 0.067 | | **1.78e-18** | |  |  | 0.097 | | **0.859** | | 0 | 0 | | |
|  | MI | 0.463 | **1.08e-10** |  | 0.053 | **2.16e-06** |  | 0.126 | **6.85e-09** |  |  | 0.113 | | **1.75e-85** | |  |  | 0 | | 0.025 | | 0 | 0 | | |
|  | DP | 0.102 | 0.003 |  | -0.036 | 0.001 |  | 0.02 | 0.119 |  |  | 0.026 | | **1.81e-05** | |  |  | 0.005 | | 0.08 | | 0 | 0 | | |
|  | AN | 0.097 | 0.005 |  | 0.015 | 0.156 |  | 0.028 | 0.04 |  |  | 0.060 | | **3.94e-22** | |  |  | 0.004 | | 0.071 | | 0 | 0 | | |
| HDL | AF | -0.077 | 0.004 | **9.75e-16** | -0.125 | **1.04e-08** | **0** | -0.03 | 0.021 | **9.36e-12** | | -0.014 | | 0.009 | | **2.49e-55** | | 0.001 | | 0.025 | | 0 | 0 | | |
|  | AP | -0.231 | **1.83e-16** |  | -0.237 | **0** |  | -0.078 | **1.72e-12** |  |  | -0.057 | | **3.83e-54** | |  |  | -0.001 | | 0.032 | | 0 | 0 | | |
|  | CHD | -0.218 | **1.39e-16** |  | -0.220 | **1.12e-13** |  | -0.078 | **1.34e-12** |  |  | -0.051 | | **6.91e-42** | |  |  | 0 | | 0.01 | | -0.218 | **1** | | |
|  | HF | -0.053 | 0.008 |  | -0.135 | **9.21e-14** |  | -0.018 | 0.022 |  |  | -0.010 | | 0.002 | |  |  | -0.001 | | 0.029 | | 0 | 0 | | |
|  | MI | -0.249 | **1.15e-15** |  | -0.276 | **0** |  | -0.069 | **6.11e-11** |  |  | -0.052 | | **5.11e-38** | |  |  | 0 | | 0.014 | | 0 | 0 | | |
|  | DP | -0.031 | 0.071 |  | -0.029 | **9.53e-08** |  | -0.002 | 0.753 |  |  | -0.007 | | 0.075 | |  |  | -0.001 | | 0.052 | | 0 | 0 | | |
|  | AN | -0.008 | 0.653 |  | -0.057 | **9.10e-15** |  | -0.002 | 0.823 |  |  | -0.020 | | **5.72e-06** | |  |  | 0 | | 0.01 | | 0 | 0 | | |
| LDL | AF | 0.089 | 0.015 | **3.51e-19** | -0.094 | 0.004 | **3.54e-04** | 0.019 | 0.273 | **1.51e-08** | | 0.020 | | 0.006 | | **5.62e-114** | | -0.049 | | **0.517** | | 0 | 0 | | |
|  | AP | 0.421 | **8.24e-20** |  | 0.020 | 0.129 |  | 0.106 | **2.15e-09** |  |  | 0.121 | | **1.11e-79** | |  |  | 0.023 | | 0.366 | | 0 | 0 | | |
|  | CHD | 0.397 | **5.02e-20** |  | -0.008 | 0.515 |  | 0.102 | **1.05e-08** |  |  | 0.123 | | **1.90e-101** | |  |  | 0.019 | | 0.351 | | 0 | 0 | | |
|  | HF | 0.010 | 0.702 |  | -0.079 | **5.05e-05** |  | 0.02 | 0.048 |  |  | 0.005 | | 0.362 | |  |  | -0.075 | | **0.833** | | 0 | 0 | | |
|  | MI | 0.442 | **2.22e-18** |  | -0.032 | 0.038 |  | 0.094 | **1.18e-07** |  |  | 0.105 | | **2.30e-68** | |  |  | 0.02 | | 0.324 | | 0.441 | **1** | | |
|  | DP | -0.026 | 0.162 |  | -0.021 | **6.86e-04** |  | -0.021 | 0.017 |  |  | -0.001 | | 0.737 | |  |  | -0.01 | | 0.268 | | 0 | 0 | | |
|  | AN | 0.017 | 0.380 |  | -0.007 | 0.362 |  | -0.012 | 0.177 |  |  | 0.014 | | 0.005 | |  |  | 0.002 | | 0.07 | | 0 | 0 | | |
| TG | AF | 0.067 | 0.016 | **5.87e-23** | -0.115 | **1.20e-14** | **8.39e-14** | 0.013 | 0.385 | **1.56e-17** | | 0.005 | | 0.418 | | **5.31e-114** | | 0 | | 0.008 | | 0 | 0 | | |
|  | AP | 0.301 | **8.39e-24** |  | 0.102 | **6.93e-14** |  | 0.11 | **2.22e-18** |  |  | 0.095 | | **1.39e-76** | |  |  | 0.055 | | **0.997** | | 0.305 | **1** | | |
|  | CHD | 0.261 | **2.01e-21** |  | 0.105 | **5.48e-14** |  | 0.101 | **1.03e-15** |  |  | 0.092 | | **1.00e-99** | |  |  | 0.037 | | **0.981** | | 0 | 0 | | |
|  | HF | 0.019 | 0.377 |  | -0.072 | **7.69e-04** |  | 0.004 | 0.612 |  |  | 0.001 | | 0.819 | |  |  | 0 | | 0.018 | | 0 | 0 | | |
|  | MI | 0.275 | **2.11e-16** |  | 0.071 | **1.23e-10** |  | 0.081 | **7.66e-12** |  |  | 0.074 | | **3.09e-63** | |  |  | 0.003 | | 0.088 | | 0 | 0 | | |
|  | DP | 0.033 | 0.057 |  | -0.019 | 0.005 |  | 0.015 | 0.073 |  |  | 0.010 | | 0.044 | |  |  | 0.019 | | **0.552** | | 0 | 0 | | |
|  | AN | -0.001 | 0.947 |  | -0.113 | **4.48e-13** |  | 0.016 | 0.082 |  |  | 0.002 | | 0.708 | |  |  | -0.001 | | 0.037 | | 0 | 0 | | |

Bonferroni adjusted $p$-value threshold is $1.02\times{10}^{-3}$ and $7.14\times{10}^{-3}$ for single and global test, respectively. Values are bold if $p$-values less than Bonferroni adjusted $p$-values or PIPs larger than 0.5. Posterior inclusion probability: PIP.

## Supplementary Table 13 Results of shared exposure detection analysis based on brain-gut axis

| Exposure | Outcome | IVW-R |  |  | MRAID |  |  | MR-APSS | | |  | | METEOR | |  | |  | | MR^2^ | | MrDAG | | |  |  |
| --- | --- | --- | --- | --- | --- | --- | --- | --- | --- | --- | --- | --- | --- | --- | --- | --- | --- | --- | --- | --- | --- | --- | --- | --- | --- |
|  |  | Effect | P (single) | P (global) | Effect | P (single) | P (global) | Effect | P (single) | P (global) | | Effect | | P (single) | | P (global) | | 0.209 | | **1** | | 0.005 | 0.03 | |  |
| BMI | GDU | 0.191 | **1.22e-06** | **1.63e-06** | 0.008 | 0.198 | **2.85e-09** | 0.034 | **3.373-04** | 0.002 | | 0.046 | | **2.00e-22** | | **1.35e-32** | | 0.153 | | **1** | | 0.06 | 0.454 | | |
|  | GERD | 0.137 | **2.712e-07** |  | -0.022 | 0.002 |  | 0.034 | 0.0012 |  |  | 0.048 | | **4.61e-20** | |  |  | -0.001 | | 0.034 | | 0 | 0 | | |
|  | IBD | -0.043 | 0.374 |  | -0.04 | **5.11e-06** |  | -0.006 | 0.554 |  |  | -0.009 | | 0.044 | |  |  | 0.001 | | 0.028 | | 0 | 0 | | |
|  | IBS | 0.028 | 0.447 |  | -0.066 | **4.75e-10** |  | -0.005 | 0.593 |  |  | -0.001 | | 0.833 | |  |  | 0.107 | | **1** | | 0.023 | 0.268 | | |
|  | DP | 0.101 | **6.02e-05** |  | -0.031 | **8.14e-06** |  | 0.014 | 0.221 |  |  | 0.006 | | 0.111 | |  |  | 0.001 | | 0.03 | | 0 | 0 | | |
|  | AN | 0.024 | 0.383 |  | -0.037 | **3.61e-07** |  | -0.004 | 0.775 |  |  | 0.005 | | 0.305 | |  |  | 0.091 | | **0.701** | | -0.007 | 0.056 | | |
| T2D | GDU | 0.041 | 0.579 | 1 | -0.012 | 0.833 | 0.384 | 0.129 | 0.473 |  | | -0.012 | | 0.837 | | 0.367 | | -0.113 | | **0.661** | | 0.013 | 0.032 | | |
|  | GERD | -0.045 | 0.525 |  | -0.121 | 0.064 |  | 0.105 | 0.708 |  | | -0.125 | | 0.029 | |  |  | 0.054 | | **0.719** | | 0.002 | 0.005 | | |
|  | IBD | -0.308 | 0.342 |  | -0.026 | 0.65 |  | 0.248 | 0.308 |  | | -0.03 | | 0.607 | |  |  | -0.24 | | **0.683** | | -0.016 | 0.06 | | |
|  | IBS | -0.070 | 0.357 |  | -0.044 | 0.472 |  | -0.067 | 0.794 |  | | -0.051 | | 0.384 | |  |  | 0.223 | | **0.706** | | 0.002 | 0.05 | | |
|  | DP | 0.067 | 0.331 |  | 0.05 | 0.38 |  | 0.259 | 0.459 | 1 | | 0.044 | | 0.427 | |  |  | -0.09 | | **0.686** | | -0.007 | 0.058 | | |
|  | AN | -0.066 | 0.452 |  | 0.029 | 0.66 |  | 0.011 | 0.959 |  | | 0.017 | | 0.779 | |  |  | 0.005 | | 0.068 | | 0 | 0 | | |
| SBP | GDU | 0.054 | 0.35 | 1 | -0.002 | 0.742 | **1.17e-13** | 0.016 | 0.145 | 0.426 | | 0.039 | | **4.87e-10** | | **3.10e-24** | | -0.003 | | 0.063 | | 0 | 0 | | |
|  | GERD | -0.038 | 0.308 |  | -0.053 | **1.95e-14** |  | -0.01 | 0.379 |  |  | -0.021 | | 0.002 | |  |  | 0.014 | | 0.134 | | 0 | 0 | | |
|  | IBD | 0.1 | 0.192 |  | -0.026 | 0.036 |  | 0.024 | 0.071 |  |  | 0.027 | | **2.33e-05** | |  |  | 0.001 | | 0.033 | | 0 | 0 | | |
|  | IBS | 0.038 | 0.479 |  | -0.056 | 0.175 |  | 0.004 | 0.695 |  |  | 0.028 | | **2.63e-06** | |  |  | 0.001 | | 0.026 | | 0 | 0 | | |
|  | DP | 0.028 | 0.429 |  | -0.042 | 0.005 |  | 0.005 | 0.671 |  |  | 0.015 | | 0.068 | |  |  | -0.001 | | 0.023 | | 0 | 0 | | |
|  | AN | -0.009 | 0.817 |  | -0.012 | 0.286 |  | 0.009 | 0.474 |  |  | 0.042 | | **1.60e-06** | |  |  | -0.001 | | 0.03 | | 0 | 0 | | |
| DBP | GDU | -0.013 | 0.819 | 0.018 | -0.001 | 0.892 | **3.45e-08** | -0.005 | 0.684 | 0.240 | | 0.024 | | **8.31e-04** | | **3.63e-24** | | 0 | | 0.019 | | 0 | 0 | | |
|  | GERD | 0.018 | 0.613 |  | -0.031 | **3.01e-05** |  | 0.004 | 0.709 |  |  | 0.006 | | 0.435 | |  |  | 0.001 | | 0.034 | | 0 | 0 | | |
|  | IBD | 0.038 | 0.607 |  | -0.6 | 0.006 |  | 0.02 | 0.15 |  |  | -0.023 | | 0.04 | |  |  | -0.004 | | 0.06 | | 0 | 0 | | |
|  | IBS | -0.026 | 0.64 |  | -0.11 | **5.75e-09** |  | -0.006 | 0.596 |  |  | 0.027 | | **1.22e-06** | |  |  | 0.018 | | 0.194 | | 0 | 0 | | |
|  | DP | 0.102 | 0.003 |  | -0.035 | 0.0013 |  | 0.02 | 0.119 |  |  | 0.026 | | **2.72e-06** | |  |  | 0.019 | | 0.178 | | 0 | 0 | | |
|  | AN | 0.097 | 0.005 |  | 0.016 | 0.091 |  | 0.028 | 0.040 |  |  | 0.057 | | **5.81e-19** | |  |  | 0.004 | | 0.075 | | 0 | 0 | | |
| HDL | GDU | 0.047 | 0.093 | 0.126 | 9.46E-04 | 0.846 | **1.09e-13** | -0.005 | 0.42 | 0.456 | | 0.009 | | 0.018 | | **3.20e-12** | | -0.003 | | 0.079 | | 0 | 0 | | |
|  | GERD | -0.038 | 0.021 |  | -0.077 | 0.011 |  | -0.012 | 0.078 |  |  | -0.018 | | **2.58e-07** | |  |  | -0.031 | | 0.373 | | 0 | 0 | | |
|  | IBD | -0.084 | 0.029 |  | -0.295 | 0.034 |  | -0.015 | 0.076 |  |  | -0.009 | | 0.09 | |  |  | -0.001 | | 0.025 | | 0 | 0 | | |
|  | IBS | -0.043 | 0.1 |  | -0.08 | 0.07 |  | -0.009 | 0.16 |  |  | -0.014 | | **1.39e-04** | |  |  | -0.001 | | 0.047 | | 0 | 0 | | |
|  | DP | -0.031 | 0.071 |  | -0.032 | **3.38e-07** |  | -0.002 | 0.753 |  |  | -0.007 | | 0.069 | |  |  | 0 | | 0.009 | | 0 | 0 | | |
|  | AN | -0.008 | 0.653 |  | -0.057 | **1.82e-14** |  | -0.001 | 0.823 |  |  | -0.02 | | **8.09e-06** | |  |  | 0.001 | | 0.027 | | 0 | 0 | | |
| LDL | GDU | 0.014 | 0.691 | 0.204 | -0.025 | **4.97e-04** | **3.48e-04** | -0.003 | 0.677 |  | | -0.001 | | 0.763 | | **2.77e-05** | | -0.003 | | 0.084 | | 0 | 0 | | |
|  | GERD | -0.044 | 0.034 |  | -0.05 | **5.80e-05** |  | -0.017 | 0.047 |  |  | -0.018 | | **1.48e-04** | |  |  | 0 | | 0.026 | | 0 | 0 | | |
|  | IBD | -0.025 | 0.591 |  | -0.484 | 0.003 |  | -0.008 | 0.481 |  |  | 0.003 | | 0.613 | |  |  | 0 | | 0.019 | | 0 | 0 | | |
|  | IBS | -0.02 | 0.517 |  | -0.017 | 0.004 |  | -0.01 | 0.214 |  |  | 1.55E-04 | | 0.968 | |  |  | -0.004 | | 0.103 | | 0 | 0 | | |
|  | DP | -0.026 | 0.162 |  | -0.022 | 0.003 |  | -0.021 | 0.017 |  |  | -0.002 | | 0.642 | |  |  | 0.003 | | 0.096 | | 0 | 0 | | |
|  | AN | 0.017 | 0.38 |  | -0.004 | 0.551 |  | -0.012 | 0.177 |  |  | 0.014 | | 0.004 | |  |  | 0.002 | | 0.05 | | 0 | 0 | | |
| TG | GDU | 0.048 | 0.089 | 0.264 | -0.001 | 0.846 | **1.55e-13** | 0.01 | 0.174 | 0.438 | | 0.016 | | **4.69e-05** | | **5.84e-06** | | 0 | | 0.016 | | 0 | 0 | | |
|  | GERD | 0.008 | 0.659 |  | -0.045 | 0.025 |  | 0.009 | 0.206 |  |  | 0.005 | | 0.21 | |  |  | 0.019 | | 0.245 | | 0 | 0 | | |
|  | IBD | 0.078 | 0.044 |  | -0.914 | 0.057 |  | 0.006 | 0.498 |  |  | 0.002 | | 0.748 | |  |  | 0.001 | | 0.02 | | 0 | 0 | | |
|  | IBS | 0.02 | 0.476 |  | -0.023 | **9.50e-05** |  | 0.007 | 0.308 |  |  | 0.010 | | 0.02 | |  |  | 0.019 | | **0.545** | | 0 | 0 | | |
|  | DP | 0.033 | 0.057 |  | -0.021 | 0.004 |  | 0.015 | 0.073 |  |  | 0.016 | | **3.89e-05** | |  |  | -0.001 | | 0.049 | | 0 | 0 | | |
|  | AN | -0.001 | 0.947 |  | -0.114 | **2.58e-14** |  | 0.016 | 0.082 |  |  | 0.010 | | 0.027 | |  |  | 0.209 | | **1** | | 0.005 | 0.03 | | |

Bonferroni adjusted $p$-value threshold is $1.19\times{10}^{-3}$ and $7.14\times{10}^{-3}$ for single and global test, respectively. Values are bold if $p$-values less than Bonferroni adjusted $p$-values or PIPs larger than 0.5. Posterior inclusion probability: PIP.

# Supplementary References

1. Siva N. 1000 Genomes project. *Nat. Biotechnol.* **26**, 256 (2008).

2. Bulik-Sullivan BK, et al. LD Score regression distinguishes confounding from polygenicity in genome-wide association studies. *Nat. Genet.* **47**, 291-295 (2015).

3. Bulik-Sullivan B, et al. An atlas of genetic correlations across human diseases and traits. *Nat. Genet.* **47**, 1236-1241 (2015).

4. Yengo L, Yang J&Visscher PM. Expectation of the intercept from bivariate LD score regression in the presence of population stratification. *BioRxiv*, 310565 (2018).

5. Lea AJ, Tung J&Zhou X. A Flexible, Efficient Binomial Mixed Model for Identifying Differential DNA Methylation in Bisulfite Sequencing Data. *PLoS Genet.* **11**, e1005650 (2015).

6. Sun S, et al. Differential expression analysis for RNAseq using Poisson mixed models. *Nucleic. Acids. Res.* **45**, e106 (2017).

7. Berzuini C, Guo H, Burgess S&Bernardinelli L. A Bayesian approach to Mendelian randomization with multiple pleiotropic variants. *Biostatistics* **21**, 86-101 (2020).

8. Dawid AP. Causal inference without counterfactuals. *J. Am. Stat. Assoc.* **95**, 407-424 (2000).

9. Dawid AP. Statistical causality from a decision-theoretic perspective. *Annu. Rev. Stat. Appl.* **2**, 273-303 (2015).

10. Berzuini C, Dawid P&Bernardinell L. *Causality: Statistical perspectives and applications*. John Wiley & Sons (2012).

11. Bowden J, et al. A framework for the investigation of pleiotropy in two‐sample summary data Mendelian randomization. *Stat. Med.* **36**, 1783-1802 (2017).

12. Hu X, et al. Mendelian randomization for causal inference accounting for pleiotropy and sample structure using genome-wide summary statistics. *Proc. Natl. Acad. Sci. U. S. A.* **119**, e2106858119 (2022).

13. Yuan Z, et al. Likelihood-based Mendelian randomization analysis with automated instrument selection and horizontal pleiotropic modeling. *Sci. Adv.* **8**, eabl5744 (2022).

14. Zuber V, et al. Multi-response Mendelian randomization: Identification of shared and distinct exposures for multimorbidity and multiple related disease outcomes. *Am. J. Hum. Genet.* **110**, 1177-1199 (2023).

15. Zuber V, Cronjé T, Cai N, Gill D&Bottolo L. Bayesian causal graphical model for joint Mendelian randomization analysis of multiple exposures and outcomes. *Am. J. Hum. Genet.* **112**, 1173-1198 (2025).

16. Deng Y, et al. A Bayesian approach for two‐stage multivariate Mendelian randomization with mixed outcomes. *Stat. Med.* **42**, 2241-2256 (2023).

17. Deng Y, Tu D, O'Callaghan CJ, Liu G&Xu W. Two-stage multivariate Mendelian randomization on multiple outcomes with mixed distributions. *Stat. Methods Med. Res.* **32**, 1543-1558 (2023).

18. Bycroft C, et al. The UK Biobank resource with deep phenotyping and genomic data. *Nature* **562**, 203-209 (2018).

19. Kurki MI, et al. FinnGen provides genetic insights from a well-phenotyped isolated population. *Nature* **613**, 508-518 (2023).

20. Ference BA, et al. Low-density lipoproteins cause atherosclerotic cardiovascular disease. 1. Evidence from genetic, epidemiologic, and clinical studies. A consensus statement from the European Atherosclerosis Society Consensus Panel. *Eur. Heart J.* **38**, 2459-2472 (2017).

21. Lyall DM, et al. Association of Body Mass Index With Cardiometabolic Disease in the UK Biobank: A Mendelian Randomization Study. *JAMA Cardiol* **2**, 882-889 (2017).

22. Dale CE, et al. Causal Associations of Adiposity and Body Fat Distribution With Coronary Heart Disease, Stroke Subtypes, and Type 2 Diabetes Mellitus: A Mendelian Randomization Analysis. *Circulation* **135**, 2373-2388 (2017).

23. Mancia G&Grassi G. The Autonomic Nervous System and Hypertension. *Circ. Res.* **114**, 1804-1814 (2014).

24. Malpas SC. Sympathetic nervous system overactivity and its role in the development of cardiovascular disease. *Physiol. Rev.* **90**, 513-557 (2010).

25. Alvares GA, Quintana DS, Hickie IB&Guastella AJ. Autonomic nervous system dysfunction in psychiatric disorders and the impact of psychotropic medications: a systematic review and meta-analysis. *J. Neuropsychiatry Clin. Neurosci.* **41**, 89 (2016).

26. Aboukhater D, et al. Inflammation and hypertension: Underlying mechanisms and emerging understandings. *J. Cell. Physiol.* **238**, 1148-1159 (2023).

27. Wirtz PH, et al. Evidence for altered hypothalamus-pituitary-adrenal axis functioning in systemic hypertension: blunted cortisol response to awakening and lower negative feedback sensitivity. *Psychoneuroendocrinology* **32**, 430-436 (2007).

28. Drożdż D, Drożdż M&Wójcik M. Endothelial dysfunction as a factor leading to arterial hypertension. *Pediatr. Nephrol.* **38**, 2973-2985 (2023).

29. Harrison NA, Cooper E, Voon V, Miles K&Critchley HD. Central autonomic network mediates cardiovascular responses to acute inflammation: relevance to increased cardiovascular risk in depression? *Brain, Behav., Immun.* **31**, 189-196 (2013).

30. Hansson GK. Inflammation, atherosclerosis, and coronary artery disease. *N. Engl. J. Med.* **352**, 1685-1695 (2005).

31. Dantzer R, O'Connor JC, Freund GG, Johnson RW&Kelley KW. From inflammation to sickness and depression: when the immune system subjugates the brain. *Nat. Rev. Neurosci.* **9**, 46-56 (2008).

32. Maqoud F, et al. Role of increasing body mass index in gut barrier dysfunction, systemic inflammation, and metabolic dysregulation in obesity. **17**, 72 (2024).

33. He B, Ji D&Zhang BJBP. Hypertension and its correlation with autonomic nervous system dysfunction, heart rate variability and chronic inflammation. **33**, 2405156 (2024).

34. Khlevner J, Park Y&Margolis KG. Brain-Gut Axis: Clinical Implications. *Gastroenterology clinics of North America* **47**, 727-739 (2018).

35. van der Graaf A, et al. Mendelian randomization while jointly modeling cis genetics identifies causal relationships between gene expression and lipids. *Nat. Commun.* **11**, 4930 (2020).
